# High-Entropy Hexagonal-Phase Oxide Hollow Polyhedrons for Highly Efficient Electrocatalytic Reduction of Low-Concentration NO

**DOI:** 10.1021/jacs.5c20787

**Published:** 2026-02-20

**Authors:** Dongdong Wang, Yan Guo, Deyan Luan, Xiaojun Gu, Xiong Wen David Lou

**Affiliations:** † Department of Chemistry, 53025 City University of Hong Kong, 83 Tat Chee Avenue, Kowloon 999077, Hong Kong, China; ‡ School of Chemistry and Chemical Engineering, 12576 Inner Mongolia University, Hohhot 010021, China

## Abstract

The electrochemical nitric oxide (NO) valorization strategy reconciles industrial emission mitigation with distributed ammonia (NH_3_) production, offering a dual solution for deteriorating urban air quality and fertilizer-deprived agricultural regions. Rational engineering of active sites constitutes the cornerstone for overcoming this catalytic bottleneck. Herein, we report a chemical etching-coordination strategy that enables the precise construction of hollow-architected high-entropy oxides (HEOs) with a nanoporous shell and customizable multimetallic compositions spanning quinary to decenary systems. Employing RuFeCoNiCuZnO as the first HEO catalyst for electrocatalytic low-concentration NO (1 vol %) reduction delivers record-breaking Faraday efficiency of 99.08% and 104.03 μg h^–1^ mg_cat_^–1^ production rate for NH_3_ synthesis, outperforming FeCoNiCuZnO and some reported catalysts. The Zn–NO battery with RuFeCoNiCuZnO achieves a power density of 1.18 mW cm^–2^ and an NH_3_ yield of 69.87 μg h^–1^ mg_cat_^–1^. Experimental results demonstrate that the incorporation of Ru modifies the electronic structure and enhances NO adsorption capacity of FeCoNiCuZnO, thereby promoting NO electroreduction. This work establishes a general method to engineer HEO nanostructures, whose unique configuration offers new possibilities in catalysis and energy conversion.

## Graphical abstract

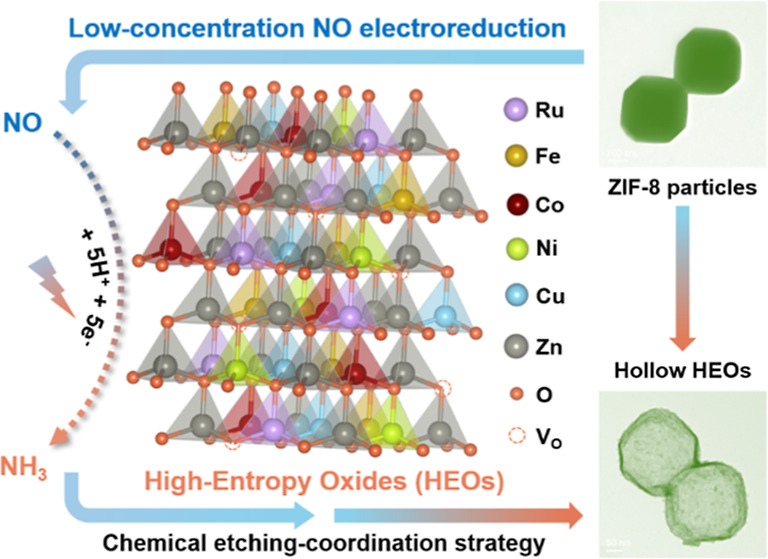

## Introduction

Nitric oxide (NO), a typical oxidized nitrogen atmospheric pollutant, originates primarily from anthropogenic activities including combustion processes in transportation, energy generation, and industrial operations.^1,2^ While NO has relatively low inherent toxicity, its atmospheric conversion products—particularly nitrogen dioxide—serve as crucial precursors for photochemical smog and acidic deposition.^3,4^ These secondary pollutants inflict substantial ecological damage, including soil acidification, aquatic eutrophication, and vegetation deterioration.^5^ From a public health perspective, NO and its derivatives provoke respiratory tract irritation, exacerbating pre-existing conditions such as asthma and chronic bronchitis.^6^ These multidimensional effects highlight the critical importance of implementing stringent NO emission and conversion regulations within integrated atmospheric protection strategies. Consequently, mitigating NO emissions represents a critical imperative for both environmental preservation and human welfare. Researchers across various fields are actively pursuing innovative solutions to address this challenge. Among existing technologies, selective catalytic reduction has gained widespread adoption due to its ability to convert NO emissions into harmless nitrogen gas.^7^ However, this approach presents notable limitations: (1) it requires sacrificial use of high-value reductants such as hydrogen or ammonia (NH_3_), and (2) it operates under elevated temperature conditions (250–400 °C).^8^ These constraints have stimulated growing interest in developing alternative strategies that can achieve NO abatement under milder conditions while utilizing more sustainable reducing agents.^9^ The electrons derived from renewable electricity in electrolytic systems represent the ideal reducing agents for NO conversion, offering both atom economy and carbon-neutral operation.^10,11^ Since the pioneering work by Xiao’s group in 2020, which first demonstrated electrochemical NO reduction to NH_3_, this field has rapidly evolved into both a major research frontier and a crucial component in modern nitrogen cycle management.^12,13^ NH_3_ is a cornerstone of modern agriculture and industry, serving as the primary feedstock for nitrogen-based fertilizers that support over 50% of global food production.^14,15^ The Haber-Bosch process remains the dominant yet problematic pillar of global ammonia production, requiring extreme operating conditions (400–500 °C, 15–25 MPa) with an iron-based catalyst system that consumes approximately 2% of the world’s energy output while generating substantial CO_2_ emissions.^16,17^ Therefore, this emerging electrocatalytic architecture enables simultaneous NO abatement and value-added N-product synthesis under mild environmental conditions.

Current research focuses on the rational design of metal catalysts with precisely engineered coordination environments to drive selective electrochemical NO-to-NH_3_ conversion.^18–21^ A critical examination of current electrocatalytic NO reduction research reveals a pervasive concentration gap.^22,23^ While catalyst development has achieved extraordinary success with pure or high-concentration NO streams (e.g., Cu electrodes demonstrating nearly 100% NH_3_ Faraday efficiency at >99.99 vol %), these advances become virtually inoperative at environmentally and industrially relevant levels (<5 vol %), evidenced by the same Cu catalysts’ performance plummeting to <10% NH_3_ Faraday efficiency under diluted NO conditions.^12,24,25^ This substantial performance gap between idealized laboratory conditions and industrial/environmental realities demands a paradigm shift toward concentration-robust catalyst design strategies that address three fundamental challenges: (1) mass-transfer limitations at low concentrations, (2) competitive adsorption against bulk electrolyte components, and (3) activation barriers specific to low-coverage surface reactions.^26,27^ The emergence of high-entropy oxides (HEOs) has revolutionized catalyst design by enabling precise electronic modulation of active centers through their unique multication composition.^28^ These complex oxides integrate five or more distinct metal species within a homogeneous crystalline framework, generating pronounced lattice strain and unconventional coordination geometries that often serve as pivotal factors in enhancing catalytic activity.^29,30^ The inherent compositional adaptability of HEOs establishes an unprecedented materials platform for orchestrating complex multistep electrocatalytic systems like nitric oxide reduction reaction (NORR) which necessitates precise stabilization of diverse reactive intermediates across successive proton–electron transfer steps. At present, high-temperature synthesis (>900 °C) routes continue to serve as the predominant techniques for producing HEOs (Figure 1a), and such elevated thermal conditions usually produce nanocrystals with substantial dimensions and broad size distributions.^31^ Since we first reported the low-temperature (400 °C) synthesis strategy for HEOs in 2019, researchers have explored a wide range of low-temperature approaches.^32^ However, the synthesis of hollow-structured HEOs presents a significant challenge in achieving uniform phase distribution within a multicomponent system, while simultaneously precisely controlling their morphology and maintaining structural stability. The low-temperature synthesis presents unique challenges, primarily kinetic limitations in precursor diffusion that require precise coordination control to achieve uniform cation incorporation, and thermodynamic competition between oxide nucleation and phase segregation.^33,34^

**Figure 1:**
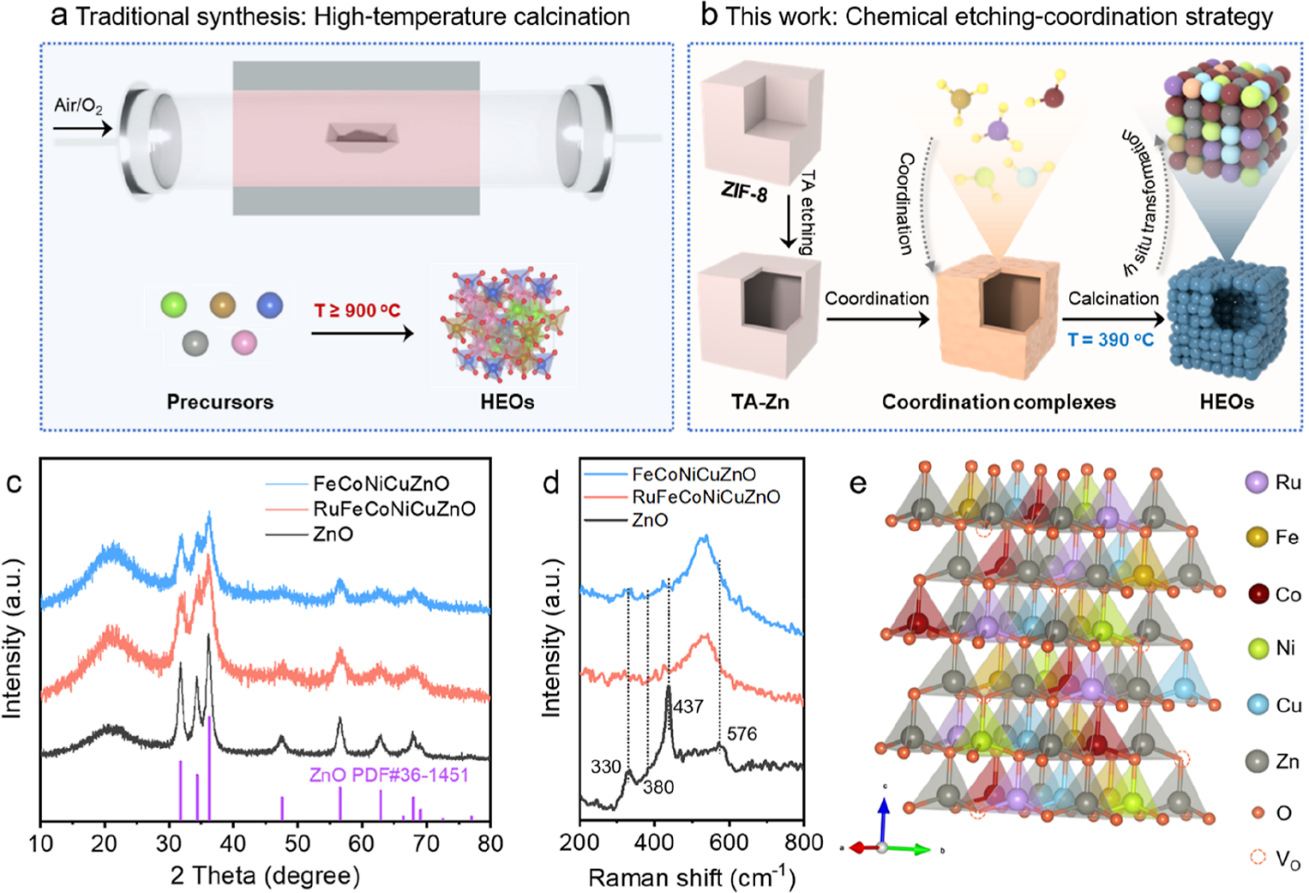
(a) The traditional synthesis method and (b) chemical etching-coordination strategy for HEOs. (c) XRD patterns and (d) Raman spectra of FeCoNiCuZnO, RuFeCoNiCuZnO, and ZnO. (e) Crystal structure model of the hexagonal phase RuFeCoNiCuZnO.

In this study, we demonstrate a universal chemical etching-coordination approach that deterministically constructs hollow HEOs with nanoporous surfaces and programmable multimetallic compositions across five to ten distinct elements. As a proof of concept, we use the representative RuFeCoNiCuZnO HEOs as electrocatalysts for NO electroreduction. Electrochemical tests reveal superior NORR activity with a high Faraday efficiency of 99.08% and an NH_3_ yield rate of 104.03 μg h^–1^ mg_cat_^–1^ under 1 vol % NO feed concentration (1% thereafter), surpassing FeCoNiCuZnO HEOs and some reported literature values. The developed Zn–NO battery integrated with the RuFeCoNiCuZnO demonstrates notable performance, delivering a power density of 1.18 mW cm^–2^ and an NH_3_ yield of 69.87 μg h^–1^ mg_cat_^–1^. NO temperature-programmed desorption (NO-TPD) experiments and X-ray absorption spectroscopy reveal that Ru incorporation in FeCoNiCuZnO HEOs enhances NO adsorption while modulating the coordination environment of active sites, thereby improving NORR. This study establishes a materials platform for efficient ammonia synthesis from dilute NO sources, broadening the utility of high-entropy oxides in electrocatalysis.

## Results and Discussion

### Material Synthesis and Characterizations

Building on these fundamental insights, the hollow high-entropy oxide architecture with precisely controlled compositions, as characterized in Figure 1b, was fabricated through a chemical etching-coordination strategy. We first engineered monodisperse zeolitic imidazolate framework-8 (ZIF-8) particles with exceptional morphological uniformity by optimizing our reported approach,^35^ as confirmed by field-emission scanning electron microscopy (FESEM) and transmission electron microscopy (TEM) analysis revealing a narrow size distribution centered at 430 nm (Figures S1 and S2, Supporting Information). Controlled tannic acid (TA) etching of ZIF-8 yielded structurally intact hollow TA-Zn, as unambiguously demonstrated through multimodal characterization. X-ray diffraction (XRD) analysis confirms the crystallographic transformation during the etching process, while FESEM and TEM reveal the resulting architectures exhibit exceptional surface uniformity and precisely defined cavity structures (Figures S3 and S4, Supporting Information). TA, a natural polyphenol, exhibits exceptional metal ion complexing properties, enabling the spontaneous formation of coordination-driven architectures via TA-metal ion coordination process.^36^ These coordination complexes combine structural stability with dynamic adaptability, making them ideal for materials design platform. The TA-mediated etching process achieves an optimal balance between ligand removal and framework reorganization, producing TA-based coordination networks for further functionalization. In this study, we strategically engineer two distinct polynuclear coordination complexes, TA-FeCoNiCuZn and TA-RuFeCoNiCuZn, through the simultaneous introduction of multiple metal ions (Fe^3+^, Co^2+^, Ni^2+^, Cu^2+^, Ru^3+^). The strong metal–ligand coordination ensures uniform dispersion of metal ions within the coordination complex, which effectively stabilizes the growth of inorganic metal sources and prevents phase separation during the calcination process.^37^ Subsequent controlled calcination at 390 °C in air transforms these precursors into hollow-structured FeCoNiCuZnO and RuFeCoNiCuZnO high-entropy oxides (Figures S5 and S6, Supporting Information).

In XRD patterns, the all peaks of the FeCoNiCuZnO and RuFeCoNiCuZnO are well indexed to the typical hexagonal ZnO, with no detectable secondary phase reflections (Figure 1c). This unequivocally confirms the successful incorporation of all metal species into the wurtzite lattice, forming a single-phase high-entropy oxide solid solution.^38^ The changes in XRD peak intensity and width can be attributed to the lattice distortion caused by the incorporation of various metals (Fe, Co, Ni, Cu, and Ru).^39^ Raman spectroscopy offers unique insights into not only lattice vibrations but also defect states in solid-state materials. As shown in Figure 1d, the Raman spectrum exhibits characteristic vibrational modes at 330, 380, 437, and 576 cm^–1^, which are assigned to E_2_(high)-E_2_(low), A_1_(TO), E_2_(high), A_1_(LO) phonon modes of wurtzite-structured ZnO, respectively.^40^ Notably, the A_1_(LO) phonon mode at 576 cm^–1^ shows enhanced intensity after implantation of other metal species, which is characteristically associated with oxygen vacancies in the wurtzite lattice.^41^ The observed low-frequency shift of the E_2_(high) and A_1_(LO) mode arise from two synergistic effects of reduced average atomic mass and increased mass fluctuations derived from oxygen vacancy generation.^42^ These characterizations reveal that metal incorporation induced substantial oxygen vacancy formation in both FeCoNiCuZnO and RuFeCoNiCuZnO high-entropy oxides. The attenuation and broadening of the E_2_(high) mode at 437 cm^–1^ primarily originate from the introduction of multiple metal species and the enhancement of oxygen vacancy defect centers, which collectively induce structural disorder in the host lattice of FeCoNiCuZnO and RuFeCoNiCuZnO high-entropy oxides.^43^ Based on these analyses, as illustrated in Figure 1e, the atomic model of RuFeCoNiCuZnO high-entropy oxides has been relatively clearly identified. Based on previous reports and our recent research, the Cu–Co/Fe dual sites enhance NORR efficiency through an optimized electronic configuration, while Ru and Ni improve catalytic performance by enhancing NO adsorption. Meanwhile, Ru plays a particularly crucial role in supplying active hydrogen species to drive the protonation process. Zn acts as a structural stabilizer for the hexagonal phase structure of the high-entropy oxides.

FESEM image reveals that the integrity of the FeCoNiCuZnO hollow structure remains intact, showing no signs of significant structural damage, as clearly observed in an unobstructed polyhedron (Figure 2a). TEM image provides direct evidence of the hollow configuration with a wall thickness of approximately 10 nm (Figure 2b). Especially notable is the surface composition of the polyhedrons, characterized by exceedingly fine oxide nanoparticles that intricately form a porous network, thereby facilitating access to active sites and intensifying the diffusion of substances (Figure 2c). Following the incorporation of Ru elements, as depicted in Figure 2d,e, the RuFeCoNiCuZnO preserves appearance and structure of the hollow polyhedrons effectively. Some uneven high-entropy oxide nanoparticles are observed in the TEM images, which is a common characteristic in the synthesis of high-entropy materials due to kinetic challenges in simultaneous multielement nucleation. Subsequent electrochemical tests will demonstrate that the consistent bulk properties and highly reproducible catalytic performance across batches confirm that the electrochemical behavior is robust and not adversely affected by this microscale variation. An image captured through high-resolution TEM (HRTEM) displays a lattice spacing of 0.26 nm, aligning with the (002) crystal plane (Figure 2f). This structural information is consistent with that of FeCoNiCuZnO high-entropy oxides (Figure S7, Supporting Information). Additionally, the high-angle annular dark-field scanning transmission electron microscopy (HAADF-STEM) image, coupled with elemental mapping images, reveal the uniform distribution of Fe, Co, Ni, Cu, Zn, and O elements within the FeCoNiCuZnO (Figure 2g). Similarly, the HAADF-STEM image and elemental mapping images illustrate the even dispersion of Ru, Fe, Co, Ni, Cu, Zn, and O elements across the RuFeCoNiCuZnO (Figure 2h). The corresponding energy-dispersive X-ray spectroscopy (EDS) spectra and inductively coupled plasma optical emission spectroscopy (ICP-OES) measurements provide further confirmation of the presence of these elements (Figure S8, Tables S1, and S2, Supporting Information). Based on these characterizations and analyses, this work establishes a facile and time-efficient strategy for synthesizing high-entropy oxides with hexagonal phase structure, achieving well-defined hollow nanostructures (approximately 250 nm) under mild calcination conditions.

**Figure 2:**
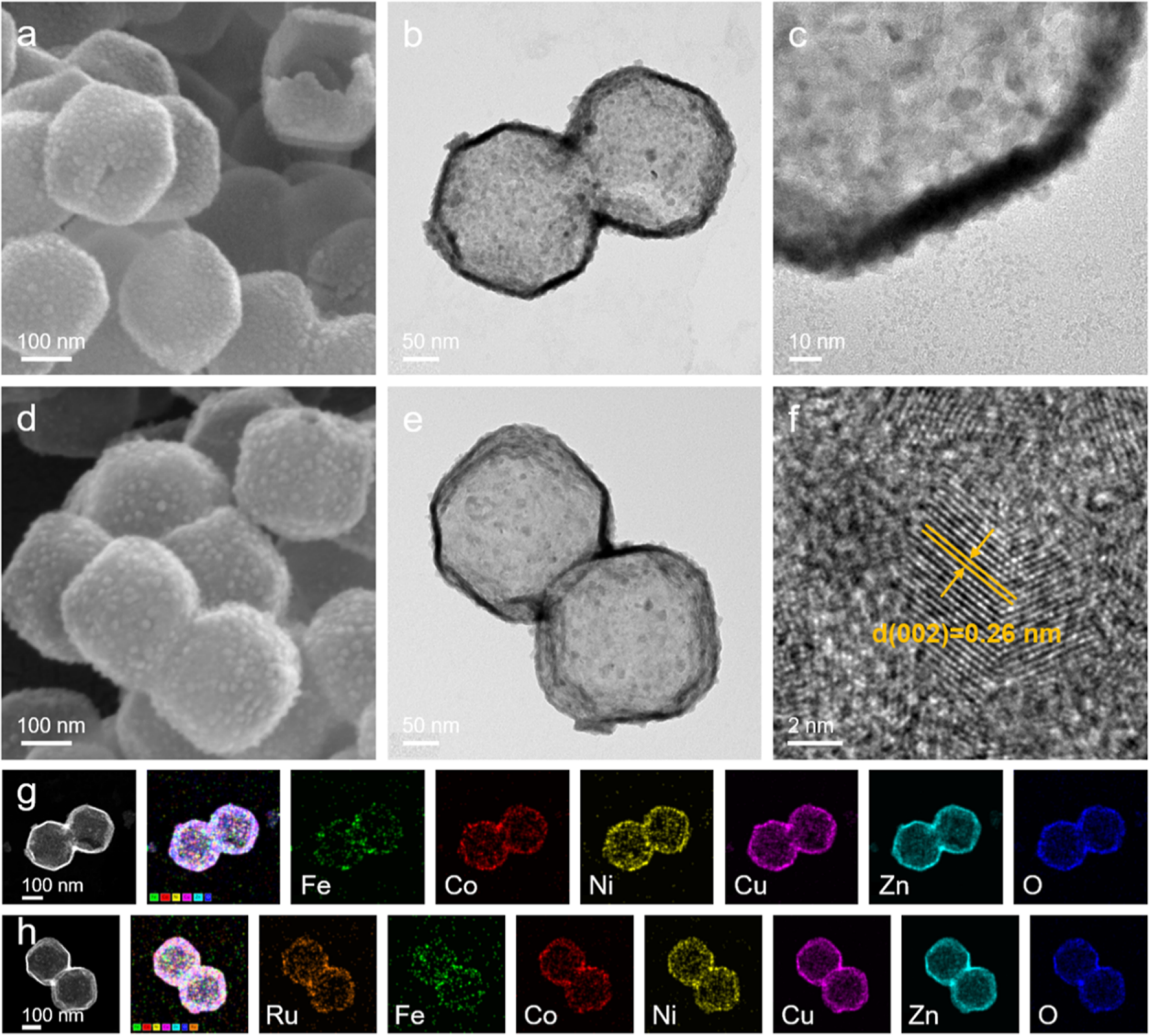
(a) FESEM image, (b) TEM image and (c) partially enlarged TEM image of the FeCoNiCuZnO. (d) FESEM image, (e) TEM image and (f) HRTEM image of the RuFeCoNiCuZnO. (g) HAADF-STEM image and the corresponding elemental mapping images of FeCoNiCuZnO. (h) HAADF-STEM image and the corresponding elemental mapping images of RuFeCoNiCuZnO.

The X-ray photoelectron spectroscopy (XPS) was utilized to explore the surface chemical state of FeCoNiCuZnO and RuFeCoNiCuZnO. XPS survey spectra demonstrates the existence of Ru, Fe, Co, Ni, Cu, Zn, and O elements on the surface of the corresponding high-entropy oxides (Figure S9, Supporting Information). For the FeCoNiCuZnO HEOs (Figure S10, Supporting Information), the high-resolution XPS of Zn 2p exhibits distinct peaks at 1044.24 and 1021.19 eV, assigned to Zn 2p_1/2_ and Zn 2p_3/2_ of Zn^2+^, respectively. The Fe 2p signals at 712.83 and 725.93 eV accompanied by a satellite (Sat.) peak at 718.50 eV indicate the presence of Fe^3+^, while the peaks at 710.30 and 723.40 eV with a satellite signal at 716.34 eV are characteristic of Fe^2+^. In the fine-scan Co 2p XPS spectra, the peaks at 779.60, 781.10, and 787.22 eV are associated with Co^3+^, Co^2+^, and satellite peaks, respectively. Similarly, the peaks centered at 854.25, 856.07, and 861.64 eV are ascribed to Ni^2+^, Ni^3+^, and satellite peaks, respectively. Distinct signals from satellite peaks are detected in the Cu 2p XPS spectra, showcasing the typical features of Cu^2+^. The O 1s XPS spectrum is analyzed to reveal three distinct peaks at 529.75, 531.40, and 532.80 eV, representing metal–oxygen bonds (O1), oxygen vacancies (O2), and surface hydroxyl groups (O3), respectively.^44^ After the integration of the Ru element into FeCoNiCuZnO, subsequent analysis reveals no notable changes in the XPS profiles of these constituents (Figure S11, Supporting Information). It is worth noting that in the detailed scan of the Ru 3p XPS spectrum, besides the peaks corresponding to the oxidation states of Ru at 463.70 and 486.0 eV, an additional peak appears at 474.25 eV, which is attributed to the Zn LM1 signal (Figure 3a). As a result of the pyrolysis process in an air atmosphere, the metal components within FeCoNiCuZnO and RuFeCoNiCuZnO primarily exhibit high oxidation states. The Ru-incorporated system exhibits a slight increase in oxygen vacancy concentration, as evidenced by enhanced intensity of the defect-associated O2 component at 531.40 eV compared to the FeCoNiCuZnO (Figure 3b). This difference provides direct spectroscopic evidence for Ru-induced oxygen vacancy formation through charge redistribution effects. The atomic radius and electronic structure of Ru differ significantly from those of typical transition metals, and its introduction induces substantial lattice distortion in the host material.^45^ Consequently, when Ru atoms are incorporated into the oxide lattice, the resulting alteration of metal–oxygen bond lengths disrupts the lattice integrity and facilitates the formation of oxygen vacancies.^46^ Notably, both FeCoNiCuZnO and RuFeCoNiCuZnO demonstrate substantially higher oxygen vacancy concentrations than ZnO synthesized via the identical method (Figure S12, Supporting Information), with these findings showing excellent consistency with Raman spectroscopic analysis.

**Figure 3:**
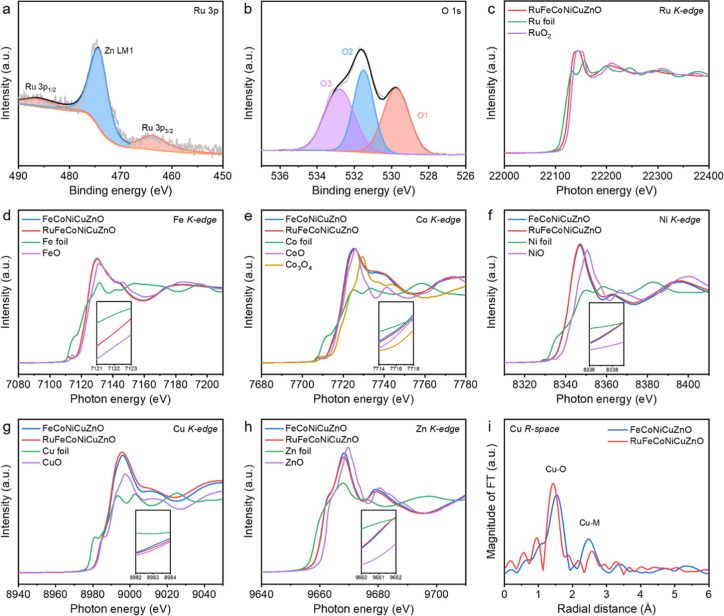
(a) Ru 3p and (b) O 1s XPS spectra of the RuFeCoNiCuZnO. Normalized K-edge XANES spectra of (c) Ru, (d) Fe, (e) Co, (f) Ni, (g) Cu, and (h) Zn for the FeCoNiCuZnO and RuFeCoNiCuZnO, along with the corresponding metal foils and standard oxide samples. (i) The corresponding Fourier transform EXAFS spectra of the FeCoNiCuZnO and RuFeCoNiCuZnO at the Cu K-edge.

For a deeper understanding of the local coordination environment and oxidation state of all metal elements in the FeCoNiCuZnO and RuFeCoNiCuZnO, characterizations by X-ray absorption near-edge structure (XANES) and extended X-ray absorption fine structure (EXAFS) with phase correction were conducted. Figure 3c illustrates the Ru K-edge XANES spectra of RuFeCoNiCuZnO, Ru foil, and RuO_2_. The absorption edge of RuFeCoNiCuZnO is situated between those of Ru foil and RuO_2_, indicating a Ru-oxidation state lower than +4. This unambiguously confirms that Ru occupies metallic lattice sites within the hexagonal HEO phase rather than forming a separate RuO_2_ phase. In the K-edge XANES spectra of transition metals in the FeCoNiCuZnO and RuFeCoNiCuZnO, their absorption edges are systematically higher in energy than the corresponding metal foils, approaching but not exceeding those of FeO, CoO, NiO, CuO, and ZnO references (Figure 3d–h). This demonstrates that their valence states are uniformly close to +2. The observed difference in the absorption edge position of the HEOs relative to reference materials arises from the complex interplay of multimetallic orbital hybridization and configurational disorder-induced electronic structure modulation unique to high-entropy systems.^47^ The corresponding EXAFS spectra clearly display two dominant peaks at 1.50 Å and 2.60 Å, attributed to metal–oxygen (M-O) coordination and metal–metal (M–M) correlations, respectively (Figure S13, Supporting Information). The coordination environments of Fe, Co, and Zn atoms remain essentially unchanged before and after Ru incorporation. The Cu sites, identified as the most active centers for electrocatalytic NH_3_ generation via NORR, display coordination characteristics that play a pivotal role in determining the reaction efficiency. Specifically, the incorporation of Ru induces a reduction of the Cu–O bond length, revealing subtle but significant modifications in the Cu coordination sphere between these two HEOs (Figures 3i; and S14 and Table S3, Supporting Information). The metal–oxygen bond lengths of other elements remain largely unchanged before and after the Ru incorporation, suggesting that the incorporated Ru atoms are likely positioned within the local structural environment of the Cu sites in the oxide lattice.^46^ The contracted Cu–O bond distance likely enhances charge transfer kinetics, thereby boosting catalytic performance.^48,49^ The peak intensity of M–M scattering in Co K-edge, Ni K-edge, and Cu K-edge EXAFS for RuFeCoNiCuZnO is lower than that of FeCoNiCuZnO, implying a potential slight lattice distortion after the incorporation of Ru atoms, resulting in vacancies at both metal and oxygen sites.^50^ These observations align with the results from Raman and XPS analyses.

Our approach enables the synthesis of various HEOs ranging from five to ten elements at low temperatures, thereby underscoring the robustness and adaptability of the chemical etching-coordination strategy. The XRD analyses of the synthesized HEOs reveal a single-phase hexagonal structure (Figure S15, Supporting Information). Furthermore, FESEM and TEM images confirm the characteristic hollow polyhedral structure, featuring a porous network architecture woven from interconnected nanoparticles (Figures S16–S23, Supporting Information). The HAADF-STEM images, together with the corresponding elemental mapping images, illustrate the uniform distribution of each constituent within these HEOs, indicating absence of phase segregation (Figure 4a–h), in agreement with EDS results (Figure S24, Supporting Information). These findings underscore the ability of our method to expand toward more intricate HEOs with hollow polyhedral structure by carefully tailoring the elemental composition.

**Figure 4:**
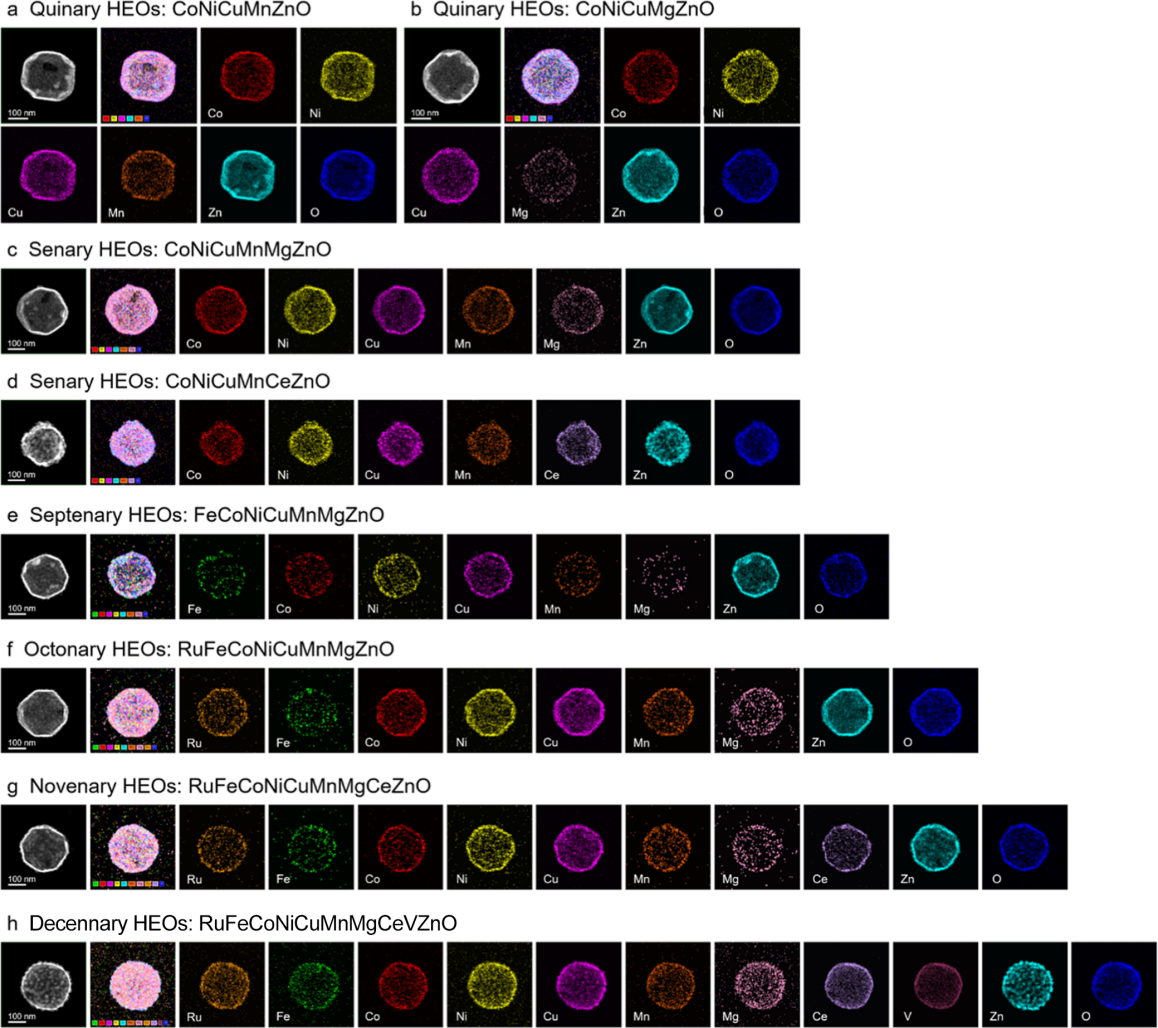
HAADF-STEM image and the corresponding elemental mapping images of HEOs: (a) CoNiCuMnZnO, (b) CoNiCuMgZnO, (c) CoNiCuMnMgZnO, (d)­CoNiCuMnCeZnO, (e) FeCoNiCuMnMgZnO, (f) RuFeCoNiCuMnMgZnO, (g) RuFeCoNiCuMnMgCeZnO, and (h) RuFeCoNiCuMnMgCeVZnO.

### Electrochemical NO Reduction Performance

As an illustration of our concept, we utilized RuFeCoNiCuZnO HEOs on a carbon paper (CP, 1 cm^2^) electrode to catalyze the reduction of 1% NO in a 0.5 M Na_2_SO_4_ electrolyte. A specialized H-cell configuration with a standard three-electrode system was employed to evaluate the electrochemical performance in synthesizing NH_3_ via NORR. Ultrahigh purity helium (He) was continuously purged through the electrochemical cell before all electrochemical tests to eliminate interference from residual gaseous impurities. In the linear sweep voltammetry (LSV) test, a notable increase in current is observed after replacing the He purge gas with 1% NO, suggesting a transition from the initial hydrogen evolution reaction (HER) to NORR (Figure 5a). Under applied potentials more negative than −0.8 V vs RHE, the LSV curves obtained in high-purity He and 1% NO exhibited near-identical characteristics, indicating dominant competition from the HER under low-concentration NO conditions. A colorimetric method is employed to quantitatively assess the produced ammonia in the range of −0.4–0.8 V versus reversible hydrogen electrode (vs RHE) for all catalysts (Figure S25, Supporting Information). The results of the potentiostatic test demonstrate that RuFeCoNiCuZnO exhibits a Faraday efficiency of 99.08% and 104.03 μg h^–1^ mg_cat_^–1^ ammonia yield, surpassing many catalysts under similar test conditions (Figures 5b and S26 and Table S4, Supporting Information). To emphasize the benefits of the Ru incorporation, an evaluation of the NORR performance of pristine FeCoNiCuZnO demonstrate a decreased Faraday efficiency of 71.29% and an ammonia generation rate of 64.97 μg h^–1^ mg_cat_^–1^ (Figures S27 and S28, Supporting Information). Product analysis confirmed the absence of byproducts including hydrazine (Figures S29 and S30, Supporting Information) and hydroxylamine (Figures S31 and S32, Supporting Information) in the electrolyte, demonstrating exceptional selectivity toward ammonia synthesis. The electrochemical surface area (ECSA) was evaluated through the double-layer capacitance (Figure S33, Supporting Information). As expected, RuFeCoNiCuZnO delivers a larger ECSA of 0.48 cm^2^ than that of FeCoNiCuZnO (0.31 cm^2^), which is consistent with the NORR performance. In the subsequent investigation, a detailed exploration is undertaken to examine influence of different Ru contents within the RuFeCoNiCuZnO on the NORR activity. Notably, as the Ru content is augmented, the material gradually transforms into a heterogeneous phase structure between RuFeCoNiCuZnO and RuO_2_ (Figure S34, Supporting Information). FESEM and TEM images further reveal that the morphologies of the hollow polyhedra are effectively preserved (Figures S35–S37, Supporting Information). The NORR performance exhibits an initial rise followed by a decline, indicating that the appropriate Ru content is crucial for enhancing the efficiency of ammonia electrosynthesis (Figure S38, Supporting Information). Furthermore, the electrocatalytic NORR activity of all synthesized high-entropy oxides are also systematically evaluated. The results demonstrate that although several catalysts exhibit notable NORR activity, their performance levels remain consistently below that of RuFeCoNiCuZnO (Figures S39 and S40, Supporting Information). The operational durability of RuFeCoNiCuZnO is rigorously assessed through successive NORR cycling tests at an applied potential of −0.4 V vs RHE, with periodic electrolyte renewal between cycles (Figures 5c; S41 and S42, Supporting Information). These experimental results demonstrate robust operational stability of RuFeCoNiCuZnO under NORR conditions. Structural analysis after the stability test reveals complete preservation of the hexagonal crystal phase, composition, and hollow polyhedral morphology in RuFeCoNiCuZnO, demonstrating exceptional structural durability under operational conditions (Figures S43 and S44, Supporting Information). Furthermore, an extended stability test is conducted at −0.4 V vs RHE for 27 h. The results demonstrate that RuFeCoNiCuZnO maintains a Faraday efficiency of 71.37% and an ammonia production rate of 70.05 μg h^–1^ mg_cat_^–1^ (Figures S45 and S46, Supporting Information). To definitively establish that the produced NH_3_ originated exclusively from the electrochemical reduction of 1% NO rather than external contaminants, comprehensive control experiments are performed (Figure S47, Supporting Information). No detectable NH_3_ is observed under no applied potential, pure He atmosphere, and bare CP working electrodes (Figure 5d). The spontaneous oxidation of NO by residual oxygen presents a fundamental challenge in NORR studies, as the resulting nitrate (NO_3_^–^) species exhibit significantly higher aqueous solubility than gaseous NO, leading to artificial inflation of apparent catalytic activity. This interference stems from nitrate’s competing reduction pathways under cathodic potentials and undetectable NO consumption prior to electrolysis, ultimately compromising catalyst evaluation accuracy. Comparative analysis of open (1% NO + Air) and sealed (1% NO) electrochemical systems reveal significantly elevated NO_3_^–^ concentration (25.55 ppm) and NH_3_ yield (119.49 μg h^–1^ mg_cat_^–1^) in the open configuration, accompanied by a higher reduction current at −0.4 V vs RHE (Figures 5e and S48–S51, Supporting Information). This systematic discrepancy quantitatively confirms our hypothesis regarding oxygen/air interference, while simultaneously demonstrating the robustness of our sealed-system protocol in maintaining NO-specific reduction pathways. These electrochemical results provide initial insights into the electrocatalytic potential of hexagonal-phase high-entropy oxides for NO-to-NH_3_ conversion, significantly expanding their structural and functional diversity. NO temperature-programmed desorption (NO-TPD) tests were utilized to explore the adsorption characteristics of NO on the FeCoNiCuZnO and RuFeCoNiCuZnO, especially crucial for the conversion process of low-concentration NO. As depicted in Figure 5f, FeCoNiCuZnO primarily exhibits some physical adsorption peaks below 200 °C, while after Ru incorporation, RuFeCoNiCuZnO shows a significant chemical adsorption peak around 320 °C, proving that Ru incorporation significantly enhanced NO adsorption capacity of FeCoNiCuZnO.

**Figure 5:**
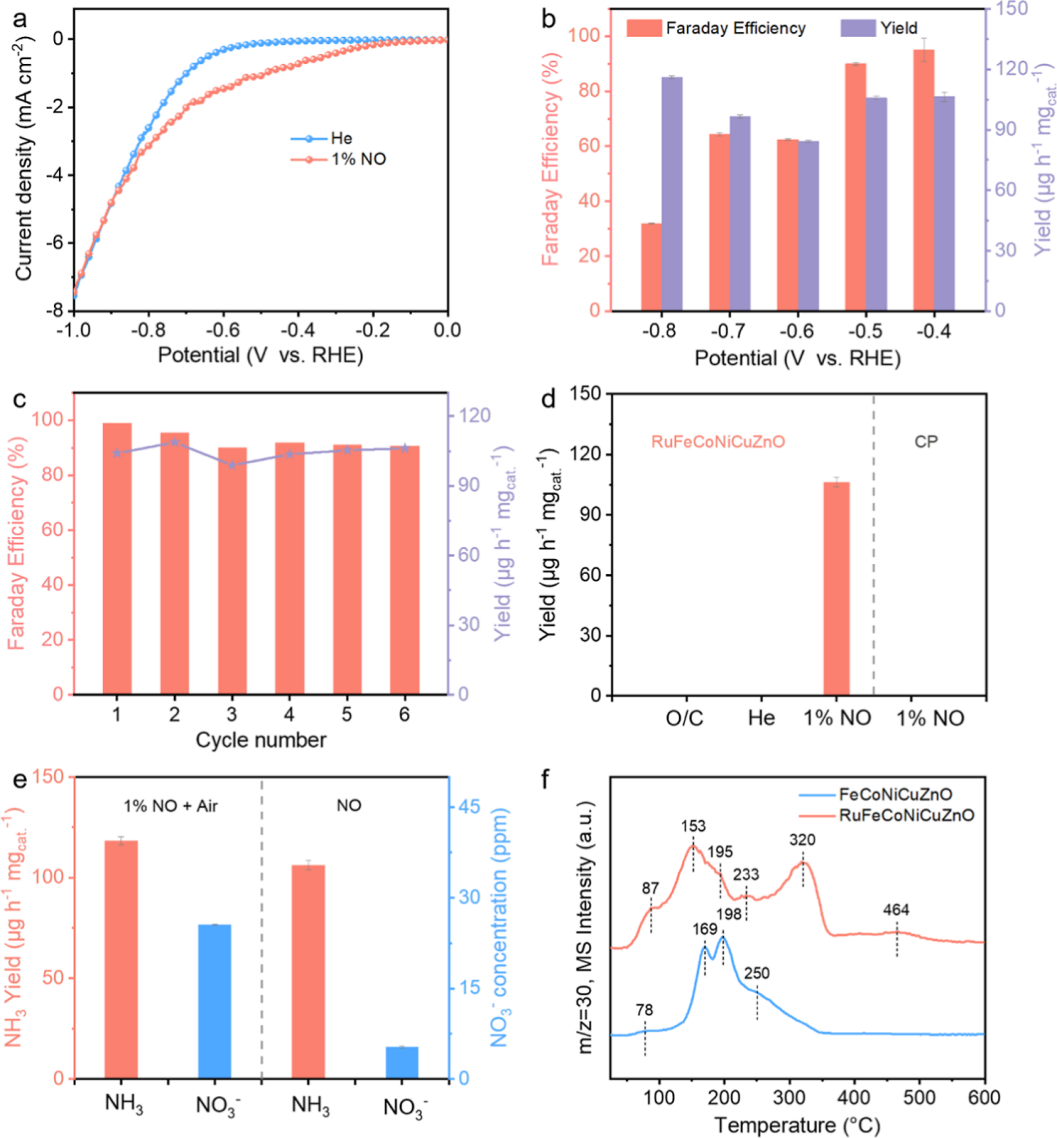
(a) LSV curves of the RuFeCoNiCuZnO in He and 1% NO-saturated 0.5 M Na_2_SO_4_. (b) Faraday efficiency and yield rate of NH_3_ at each given potential over RuFeCoNiCuZnO. (c) Faraday efficiency and yield rate of NH_3_ in six successive cycles over RuFeCoNiCuZnO at −0.4 V vs RHE. (d) Electrochemical NORR activity over RuFeCoNiCuZnO and CP under various conditions (O/C denotes the elimination of the external potential). (e) NH_3_ yield and nitrate concentration in various testing environments over RuFeCoNiCuZnO at −0.4 V vs RHE. (f) NO-TPD spectra of the FeCoNiCuZnO and RuFeCoNiCuZnO.

### Theoretical Calculations

To gain mechanistic insights into the activity-selectivity relationships of the HEOs, we performed density functional theory (DFT) calculations to evaluate the Gibbs free energy profiles of possible NORR pathways. Guided by the HRTEM, ICP-OES, and EXAFS results, the HEO(002) surfaces with random atomic distributions were selected as model systems for theoretical investigation (Figure S52a,b, Supporting Information), with the specific constraint that Ru atoms are positioned adjacent to Cu atoms. Initially, the adsorption energies of NO at various metal atomic sites on the HEO surfaces were systematically evaluated. As shown in Figure 6a, the results indicate that N-end adsorption of NO at Cu site within the HEOs is the most thermodynamically favorable (Figure S53, Supporting Information). Notably, the NO adsorption energies on FeCoNiCuZnO and RuFeCoNiCuZnO were calculated to be −0.75 eV and −1.17 eV, respectively, indicating that Ru incorporation significantly enhances NO adsorption (Figure S52c, Supporting Information). These results are in agreement with NO-TPD measurements. The projected density of states of the catalysts is also analyzed via DFT calculations (Figure S54, Supporting Information). The d-band center position of RuFeCoNiCuZnO (−1.78 eV) upshifts toward the Fermi level, which is higher than FeCoNiCuZnO (−1.99 eV), indicating that the RuFeCoNiCuZnO facilitates the adsorption of substrate or reaction intermediates. The initial protonation of *NO preferentially forms *NOH rather than *HNO, as evidenced by a lower reaction free energy (0.24 eV) for this pathway (Figure 6b). Subsequent protonation of *NOH proceeds through *N and H_2_O formation (0.57 eV), a pathway thermodynamically favored over *NHOH generation (Figure 6c). The reaction then continues through three sequential protonation steps to ultimately yield NH_3_. The NO-to-NH_3_ conversion on FeCoNiCuZnO and RuFeCoNiCuZnO catalysts proceeds through a thermodynamically favorable downhill pathway, interrupted solely by the energetically uphill *NO hydrogenation. This identifies *NO hydrogenation as the potential-determining step across the FeCoNiCuZnO and RuFeCoNiCuZnO catalytic systems. As a result, the energy barrier for RuFeCoNiCuZnO is substantially reduced to 0.24 eV, lower than that of FeCoNiCuZnO (0.43 eV), demonstrating the significant catalytic enhancement achieved through Ru incorporation (Figures 6d and S55, Supporting Information). We further evaluate the active hydrogen generation capacity of both HEO catalysts, with results demonstrating that RuFeCoNiCuZnO possesses superior capability to supply active hydrogen species for the protonation processes, thereby enhancing catalytic efficiency (Figure 6e). These DFT computational results strongly corroborate our experimental observations, providing robust theoretical support for the proposed reaction mechanism.

**Figure 6:**
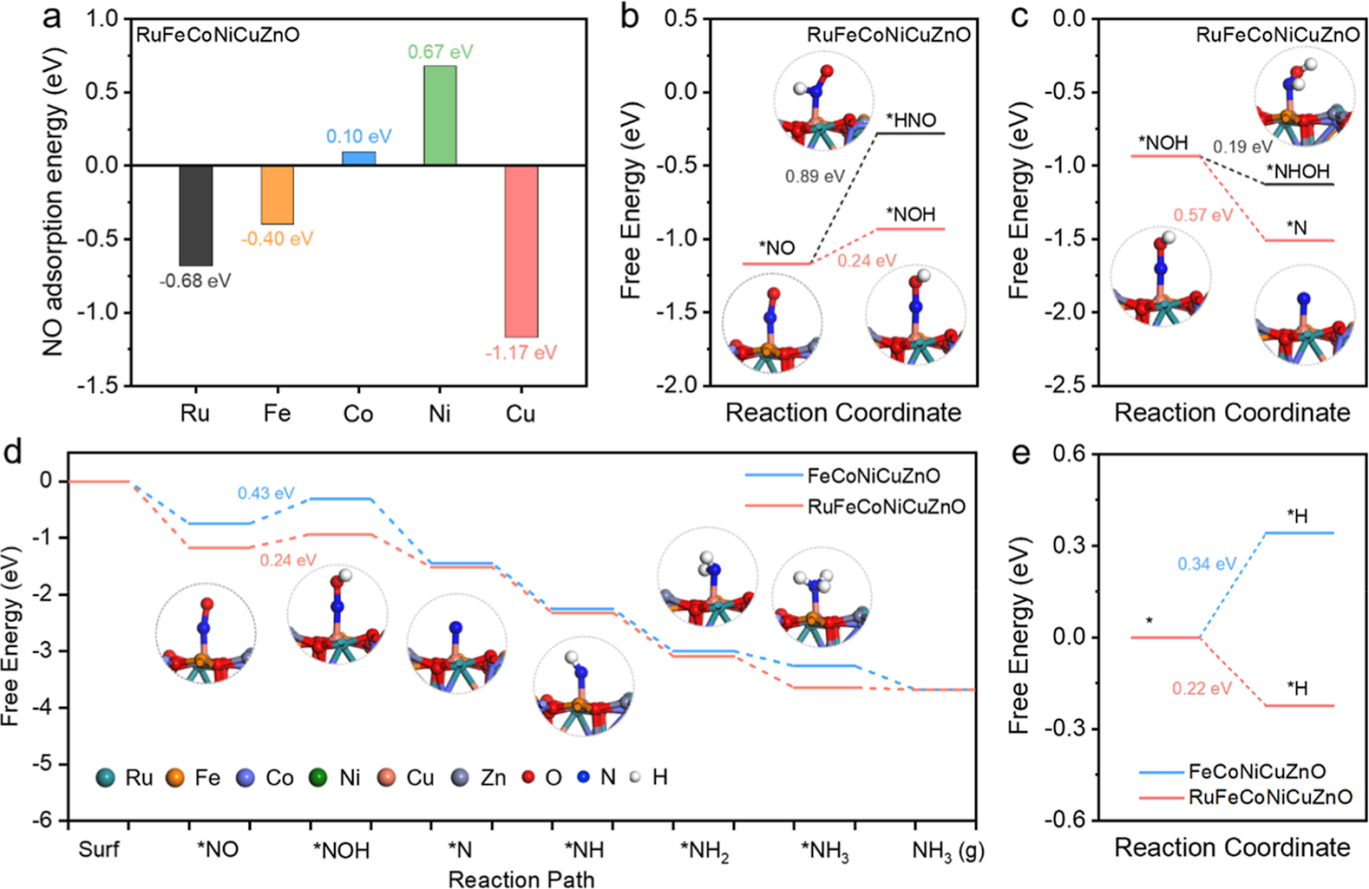
(a) Comparison of NO adsorption energies at various metal sites in RuFeCoNiCuZnO. (b) Free energy diagram for *NO → *NOH/*HNO on RuFeCoNiCuZnO. (c) Free energy diagram for *NOH → *N/*NHOH on RuFeCoNiCuZnO. (d) The free energy diagram of NORR on the FeCoNiCuZnO and RuFeCoNiCuZnO. The inset shows the optimized geometry structures of NORR intermediates over RuFeCoNiCuZnO. (e) Evaluation of active hydrogen supply capacity for FeCoNiCuZnO (Cu site) and RuFeCoNiCuZnO (Ru site).

### Zn–NO Battery Performance

Zn–NO battery with catalytic conversion mechanisms represents an innovative energy conversion technology that synergistically integrates sustainable electricity generation, environmental NO elimination, and electrochemical ammonia synthesis (Figure 7a). This technology opens new possibilities for distributed energy systems in industrial zones where NO-containing flue gases are abundant. As shown in Figure 7b, the constructed Zn–NO battery utilizing RuFeCoNiCuZnO electrodes exhibits a power density of 1.18 mW cm^–2^, which notably surpasses the power densities achieved with the bare CP (0.30 mW cm^–2^), FeCoNiCuZnO (0.64 mW cm^–2^), and some previously reported materials in Zn–NO/N_2_ batteries (Figure 7c; Table S5, Supporting Information). Despite achieving notable power output, Zn-dilute NO batteries still trail conventional Zn-air (>150 mW cm^–2^), Zn–NO_3_^–^ (>5 mW cm^–2^), and Zn-pure NO (>2 mW cm^–2^) systems.^51,52^ Prospective performance improvement may be realized through gas enrichment strategies and three-phase interface optimization. Integration of the RuFeCoNiCuZnO enables the Zn–NO battery to achieve an open-circuit voltage (OCV) of 2.04 V vs Zn, as verified by polarization curve (Figure 7d). When operated at current densities of 1–5 mA cm^–2^, the system maintains stable discharge capacity and achieves an ammonia production rate of 69.87 μg h^–1^ mg_cat_^–1^ at 5 mA cm^–2^ (Figures 7e,f and S56, Supporting Information). The Zn–NO battery also exhibits stable electrochemical behavior during charging at the same current densities, along with an energy efficiency of 19.6% at 1 mA cm^–2^ (Figure S57, Supporting Information). In order to further explore the long-term voltage stability of the Zn–NO battery during discharge, we conduct a galvanostatic test at 5 mA cm^–2^ for 23 h (Figure S58, Supporting Information). The results indicate that the voltage remains relatively stable within the first 12 h. Notably, a gradual decline is observed thereafter, which becomes more pronounced after 20 h. This voltage decay is likely attributed to partial structural collapse of the catalyst and the progressive dissolution of the Zn foil anode (Figure S59, Supporting Information). The operational continuity of Zn–NO battery hinges on NO availability, with system functionality preserved through strategic pathway switching. Upon NH_3_ production cessation, oxygen or air introduction enables transition to Zn-air battery mode. This contingency necessitates specially engineered bifunctional cathodes capable of accommodating both NO reduction and oxygen reduction reaction. The Zn–NO battery system presents distinct operational benefits over conventional electrolytic NO-to-ammonia conversion, notably its self-sustaining operation and dual-output capability for both electricity and ammonia synthesis (Table S6, Supporting Information). This technology demonstrates practical pathways to valorize industrial NO waste streams while supporting clean energy transitions through its hybrid energy-chemical production design.

**Figure 7:**
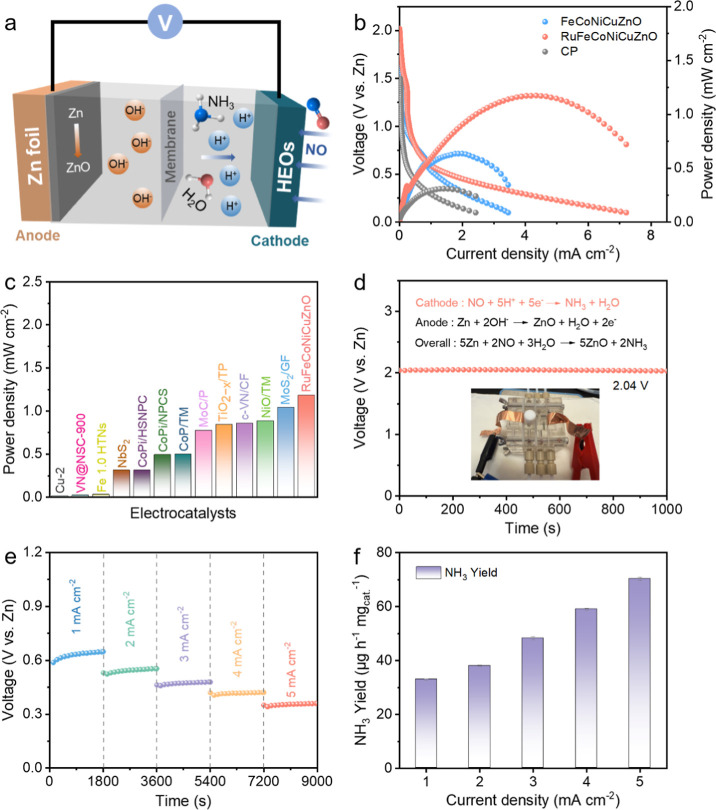
(a) Design principles of the Zn–NO battery. (b) Polarization and power density profiles of FeCoNiCuZnO, RuFeCoNiCuZnO, and CP electrodes. (c) Comparative power density analysis between the RuFeCoNiCuZnO-based system and literature-reported Zn–NO/N_2_ batteries. (d) OCV measurement for the RuFeCoNiCuZnO-based Zn–NO battery, with an inset showing the actual device. (e) Discharge profiles of the RuFeCoNiCuZnO-based Zn–NO battery across multiple current densities. (f) NH_3_ production rate under operational conditions.

## Conclusion

In conclusion, we reported a highly efficient hollow RuFeCoNiCuZnO high-entropy oxide polyhedron for sustainable NH_3_ electrosynthesis from NORR in neutral electrolyte. A series of hollow high-entropy oxide polyhedrons with elemental compositions ranging from quinary to decenary were synthesized via a chemical etching-coordination strategy. Detailed electrocatalytic investigations revealed that, compared to FeCoNiCuZnO, RuFeCoNiCuZnO delivered outstanding low-concentration NO reduction performance, including a Faraday efficiency of 99.08%, 104.03 μg h^–1^ mg_cat_^–1^ of NH_3_ yield, and excellent consecutive cycling stability. The assembled Zn–NO battery using RuFeCoNiCuZnO achieved a peak power density of 1.18 mW cm^–2^ and an NH_3_ production rate of 69.87 μg h^–1^ mg_cat_^–1^, representing a potentially advantageous strategy for environmental remediation and energy conversion. This work not only pioneers a novel low-temperature synthesis strategy for hollow high-entropy oxide polyhedrons, but also significantly expands their potential in electrocatalytic applications, opening new avenues for advanced NORR catalyst design.

## Supplementary Material



## Notes

Experimental and electrochemical details, additional structural and morphological characterizations, including XRD, EDS, FESEM, TEM, and DFT results (PDF)
