# Supplementary material for: High-Entropy Hexagonal-Phase Oxide Hollow Polyhedrons for Highly Efficient Electrocatalytic Reduction of Low-Concentration NO
Source: J Am Chem Soc. 2026 Feb 20;148(8):8711–22. doi: 10.1021/jacs.5c20787 (PMC12964408; doi:10.1021/jacs.5c20787)
Supplement: Supplementary file 1 [file ja5c20787_si_001.pdf]

Supporting Information for

High-Entropy Hexagonal-Phase Oxide Hollow Polyhedrons for Highly Efficient Electrocatalytic Reduction of Low-Concentration NO

Dongdong Wang,[†] Yan Guo,[$] Deyan Luan,[†] Xiaojun Gu,[$]* Xiong Wen (David) Lou[†]*

[†]Department of Chemistry, City University of Hong Kong, 83 Tat Chee Avenue, Kowloon, 999077, Hong Kong, China

[$]School of Chemistry and Chemical Engineering, Inner Mongolia University, Hohhot 010021, China

*Corresponding author. Email: xiaojun.gu@imu.edu.cn (X.G.); david.lou@cityu.edu.hk (X.W.L.)

Experimental Section

Chemicals**:** Cobalt chloride hexahydrate ($CoCl_2·6H_2O$), copper chloride dihydrate ($CuCl_2·2H_2O$), anhydrous ferric chloride ($FeCl_3$), nickel chloride hexahydrate ($NiCl_2·6H_2O$), manganese chloride tetrahydrate ($MnCl_2·4H_2O$), cerium chloride heptahydrate ($CeCl_3·7H_2O$), anhydrous magnesium chloride ($MgCl_2$), vanadium trichloride (VCl_3), zinc acetate dihydrate ($Zn(CH_3COO)_2·2H_2O$), 2-methylimidazole, tannic acid (TA), cetyltrimethylammonium bromide (CTAB), sodium citrate, salicylic acid, sodium hydroxide (NaOH), sodium hypochlorite (NaClO), sodium nitroferricyanide, ammonium chloride (NH_4Cl), sodium sulfate (Na_2SO_4), and sodium nitrate ($NaNO_3$) were purchased from Sigma-Aldrich. Zn foil was purchased from Thermo Fisher Scientific. Ultrapure water (18.25 MΩ/cm) was used for all experimental processes and all chemical regents were used without further purification.

Synthesis of ZIF-8**:** In a typical synthesis, 300 mg of $Zn(CH_3COO)_2·2H_2O$ was dissolved in 5 mL of deionized water to form solution A. 1160 mg of 2-methylimidazole and 0.4 mg of CTAB were dissolved in 5 mL of deionized water to form solution B. Then, solution A was poured into solution B and the mixture was shaken for about 15 s. Afterwards, the white suspension was left undisturbed at room temperature for 2 h. The resulting ZIF-8 were washed with ethanol for further use.

Synthesis of tannic acid (TA)-Zn**:** Typically, 15 mg of ZIF-8 was dispersed into 10 mL of ethanol. This solution was injected into 80 mL of mixture solution of deionized water and ethanol (vol:vol = 1:1) that containing 1 mg mL^{-1} TA. After that, the resultant solution was kept under stirring at room temperature for 14 min. The TA-Zn was collected by centrifugation, washed with ethanol for 5 times.

Synthesis of TA-FeCoNiCuZn**:** 16 mg of TA-Zn was dispersed into 20 mL of ethanol through ultrasonication for 15 min, followed by the addition of 10 mL of the mixed aqueous solution (2.5 mM) of $FeCl_3$, $CuCl_2·2H_2O$, $CoCl_2·6H_2O$, and $NiCl_2·6H_2O$. After that, this resultant solution was stirred at room temperature for 60 min. Finally, the TA-FeCoNiCuZn was collected by centrifugation, washed with ethanol for 5 times.

***Synthesis of TA-RuFeCoNiCuZn*:** The synthesis process is similar to that of TA-FeCoNiCuZn described above, except that a certain amount of $RuCl_3 \cdot xH_2O$ was added to the mixed aqueous solution. Additionally, a series of TA-RuFeCoNiCuZn with varying Ru contents were synthesized to systematically investigate the influence of Ru content (1 wt.%, 2 wt.%, 4 wt.%, and 8 wt.%) in the final oxides on catalytic activity. The resulting samples were systematically labeled as TA-RuFeCoNiCuZn-1, TA-RuFeCoNiCuZn-2, TA-RuFeCoNiCuZn-4, and TA-RuFeCoNiCuZn-8 to denote their compositional variations. Further, TA-RuFeCoNiCuZn-2 was denoted as TA-RuFeCoNiCuZn.

***Synthesis of FeCoNiCuZnO*:** The as-prepared TA-FeCoNiCuZn was first annealed at 200 °C for 30 min and then further annealed at 390 °C for 15 min with a heating rate of 1 °C min^{-1} in air, and cooled down to room temperature naturally.

***Synthesis of RuFeCoNiCuZnO*:** The as-prepared TA-RuFeCoNiCuZn-1, TA-RuFeCoNiCuZn, TA-RuFeCoNiCuZn-4, and TA-RuFeCoNiCuZn-8 were first annealed at 200 °C for 30 min and then further annealed at 390 °C for 15 min with a heating rate of 1 °C min^{-1} in air, and cooled down to room temperature naturally. The samples were named RuFeCoNiCuZnO-1, RuFeCoNiCuZnO-2, RuFeCoNiCuZnO-4, and RuFeCoNiCuZnO-8. Further, RuFeCoNiCuZnO-2 was denoted as RuFeCoNiCuZnO.

***Synthesis of ZnO*:** The as-prepared TA-Zn was first annealed at 200 °C for 30 min and then further annealed at 390 °C for 15 min with a heating rate of 1 °C min^{-1} in air, and cooled down to room temperature naturally.

***Synthesis of other quinary/senary/septenary/octonary/novenary/decernary oxides*:** The synthesis process is similar to that of FeCoNiCuZnO described above, except that a certain amount of corresponding metal chloride salt was added to the mixed aqueous solution. The samples were named CoNiCuMnZnO, CoNiCuMgZnO, CoNiCuMnMgZnO, CoNiCuMnCeZnO, FeCoNiCuMnMgZnO, RuFeCoNiCuMnMgZnO, RuFeCoNiCuMnMgCeZnO, and RuFeCoNiCuMnMgCeVZnO.

***Material characterizations*:** The morphology and structure of the samples were characterized by field-emission scanning electron microscopy (FESEM, QUATTRO S) and transmission electron microscope (TEM, FEI/Philips Tecnai 12 BioTWIN). Elemental mapping images were collected using TEM equipped with energy-dispersive X-ray spectroscopy (EDS, FEI, Tecnai G2 F20). The crystal phases of the samples were analyzed by X-ray diffraction (XRD, Bruker D2 Phaser). X-ray photoelectron spectroscopic (XPS) measurements were carried out with an ESCALAB 250Xi. The actual metal contents in the FeCoNiCuZnO and RuFeCoNiCuZnO were determined by inductively coupled plasma optical emission spectroscopy (ICP-OES, Agilent 5800). The X-ray absorption fine structure (XAFS) spectra of Co K-edge, Fe K-edge, Ni K-edge, Zn K-edge, Ru K-edge, and Cu K-edge of the FeCoNiCuZnO and RuFeCoNiCuZnO were measured at the National Synchrotron Radiation Research Center, Taiwan. The Raman spectroscopy of the FeCoNiCuZnO, RuFeCoNiCuZnO, and ZnO were measured by WITec alpha300R. The NO temperature programmed desorption measurements were performed on a Hiden DECRA chemisorption apparatus.

***Electrochemical measurements*:** Electrochemical measurements were performed in a sealed H-type electrochemical cell separated by a piece of Nafion 117 membrane with a three-electrode system at the room temperature (electrochemical workstation, Vertex.C.EIS, the ivium electrochemical measurement system). The membrane was treated firstly in H_2O_2 (5 wt.%) aqueous solution at 80 °C for 1 h. And then, it treated in 0.5 M H_2SO_4 for 2 h at 80 °C and finally in water for 6 h. The catalyst dropped on carbon paper (1×1 cm^2) was used as the working electrode (0.6 mg cm^{-2}), graphite rod was used as the counter electrode and Ag/AgCl (saturated KCl electrolyte) was used as the reference electrode. All potentials in this study were given versus reversible hydrogen electrode (vs. RHE). The electrochemical activity of electrocatalysts was measured for NO reduction reaction (NORR) in 0.5 M Na_2SO_4. Generally, 4 mg of electrocatalyst was ultrasonically dispersed in 950 μL of isopropanol and 50 μL of Nafion solution (5 wt.%, Du Pont) to form a homogeneous ink followed by dropping 150 μL of the ink onto the surface of carbon paper and dried under room temperature. Before

electrochemical tests, the electrolyte was firstly purged with high-purity He gas (99.999%) with the rate of 50 sccm for 30 min to exclude the air, and the 1 vol % NO in He gas was then fed with the rate of 50 sccm for 30 min to saturate the electrolyte and kept constant during the electrochemical tests. Electrochemical surface area (ECSA) was evaluated through the double-layer capacitance (C_{dl}) using the cyclic voltammetry (CV) measurements at different scan rates including 10, 20, 40, 60, 80 and 100 mV s^{-1}.

***Determination of NH_3*:** The yield of the NH_3 was determined by a spectrophotometry (Shimadzu, UV-2600i) measurement with the indophenol blue method. For indophenol blue method, the concentration-absorbance curves were calibrated using a standard NH_3 solution with a series of concentrations. For electrolyte, after potentiostatic electrolysis, 2 mL of reaction solution was taken into a beaker. Then the indicators of 2 mL of 1 M NaOH solution containing 5 wt.% salicylic acid and 5 wt.% sodium citrate, 1 mL of 0.05 M NaClO solution and 0.2 mL of 1 wt.% sodium nitroferricyanide solution were added into the beaker in sequence. After coloring for 2 h, the absorption spectrum of resulted solution was recorded via an ultraviolet-visible spectrophotometer.

***Determination of N_2H_4*:** The amount of hydrazine in electrolyte was measured by colorimetric method. Typically, 5 mL of the electrolyte solution was taken out and then mixed with 5 mL of the coloring solution (a mixture of 5.99 g para-(dimethylamino) benzaldehyde, 30 mL concentrated HCl and 300 mL ethanol). After 10 min, the absorbance of resulting solution was measured at a wavelength of 455 nm.

***Determination of NH_2OH*:** 100 μL of an aqueous acetate buffer (1.0 M sodium acetate + 1.0 M acetic acid), 100 μL of a 4 mM ammonium ferric sulfate aqueous solution and 100 μL of a 10 mM 1,10-phenanthroline ethanolic solution were sequentially added into 3 mL of the sample. After 30 min, the absorbance of resulting solution was measured at a wavelength of 510 nm.

The NORR performance evaluation in Zn-NO battery: A catalyst-loaded carbon paper (29 BC) electrode (0.6 mg cm^{-2}) was employed as the cathode to perform the NORR in a cathodic electrolyte

(0.5 M Na_2SO_4, 20 mL). A polished Zn plate was set in an anodic electrolyte (1 M KOH, 20 mL), and a Nafion 117 membrane was used to separate the two different electrolytes. Before all electrochemical tests (OCP, polarization curves, and discharge curves), high-purity He gas was introduced for 30 min to eliminate the residual air in the entire Zn-NO device. The catalyst loading was consistent with the three-electrode system. During the battery discharge process, the Zn-NO electrochemistry implements electrochemical NO reduction driven by Zn dissolution. In the charging test, 0.02 M $Zn(CH_3COO)_2$ was introduced as an additive into 1 M KOH aqueous solution. In this process, the oxygen evolution reaction (OER) will replace the NORR.

When the aqueous Zn-NO electrochemical cell discharges, the following reactions are assumed to possibly take place:

Cathode (0.5 M Na_2SO_4 with 1 vol % NO, pH = 7):

$$NO + 5H_2O + 5e^- \rightarrow NH_4OH + 5OH^-$$

$$E_c = E^{\theta}_{NO/NH_4OH} - \frac{RT}{nF}\ln\left(\frac{[OH^-]^5[NH_4OH]}{[NO]}\right) = 1.17$$

Anode [1 M KOH]

$$Zn - 2e^- \rightarrow Zn^{2+}$$

Zn^{2+} will react with OH^- spontaneously:

$$Zn^{2+} + 4OH^- \rightarrow Zn(OH)_4^{2-}$$

When $Zn(OH)_4^{2-}$ is over its solubility:

$$Zn(OH)_4^{2-} \rightarrow ZnO + H_2O + 2OH^-$$

$$E_a = E^{\theta}_{Zn(OH)_4^{2-}/Zn} - \frac{RT}{nF}\ln\left(\frac{[OH^-]^4}{\left[Zn(OH)_4^{2-}\right]}\right) = -1.25\ V$$

$$E_{discharge} = 1.17-(-1.25) = 2.42\ V$$

Where n, F, R, and T are electron transfer number, the Faraday constant (96485 C mol^{-1}), gas constant (8.314 J mol^{-1} K^{-1}), and reaction temperature (298.15 K), respectively. The concentrations of $[OH^-]$

in cathode cell and anode cell are 10^{-7} and 1 mol L^{-1}, respectively. Assuming $[NH_4OH]$ is 10^{-3} mol L^{-1} in cathode cell.

The energy efficiency of Zn-NO/OER batteries in this study is defined as the ratio of the energy released during discharge to the energy consumed during charge, which can be expressed as follows:

$$\text{Energy efficiency (\%)} = \frac{\int_0^t V_{\text{discharge}} * I_{\text{discharge}}\, dt}{\int_0^t V_{\text{charge}} * I_{\text{charge}}\, dt} * 100\ \%$$

Where *V*, *I*, and *t* are the discharge/charge voltage, current density, and operating time, respectively.

Calculation details: All DFT computations in this study were conducted with the Vienna Ab Initio Simulation Package (VASP).[1-2] The core-electron interaction were treated using the projector augmented wave (PAW) method. The exchange–correlation interactions were treated using the Perdew–Burke–Ernzerhof (PBE) generalized gradient approximation (GGA).[3] The structures of RuFeCoNiCuZnO and FeCoNiCuZnO were constructed by stochastic substitution of ZnO, guided by atomic ratios derived from Inductively Coupled Plasma spectroscopy. The 20 Å vacuum layer was introduced to model the catalyst surface, and a two-layer atomic setup was adopted to enhance computational efficiency-the bottom layer was fixed at its optimized position, while the top layer and any adsorbates were fully relaxed.

The Gibbs free energy change (ΔG) was evaluated as:

$$\Delta G = \Delta E + \Delta ZPE - T\Delta S$$

Here, ΔE represents the electronic energy difference from self-consistent ground-state calculations, T = 298.15 K, and ΔS is the entropy change. Entropies for gas-phase species were sourced from the NIST database, with zero-point energy (ΔZPE) and entropy corrections incorporated into the adsorption energies.

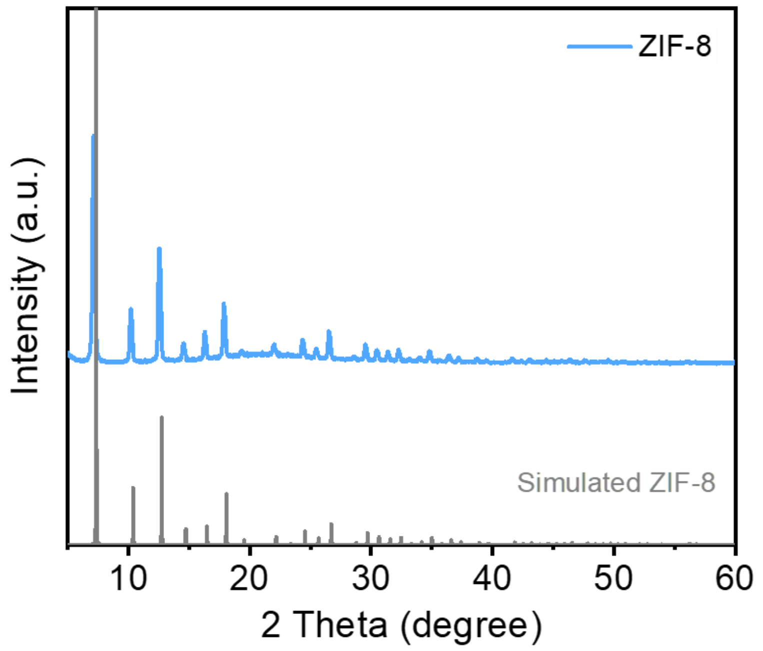

Figure S1. XRD pattern of the ZIF-8.

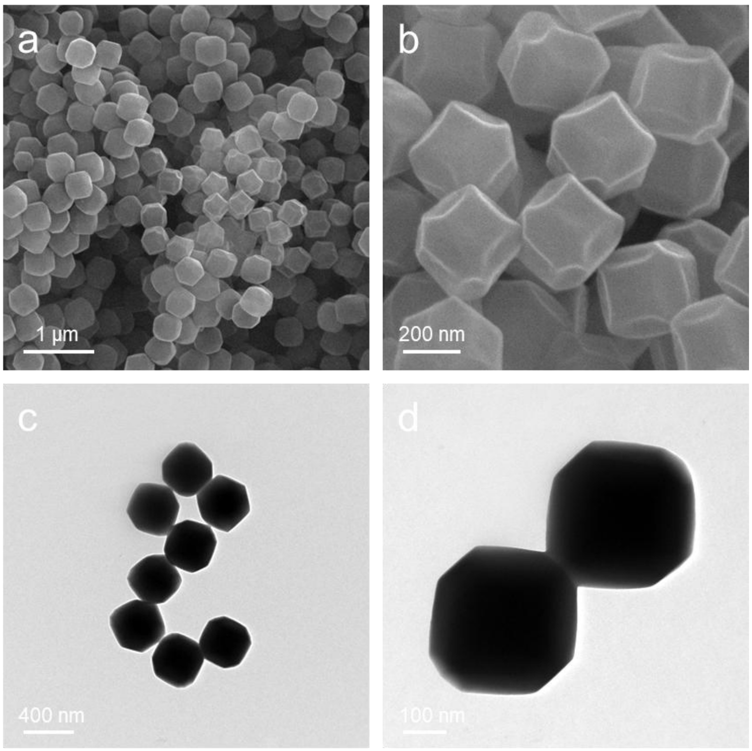

Figure S2. Low-magnification FESEM and TEM images of ZIF-8 (a and c). High-magnification FESEM and TEM images of ZIF-8 (b and d).

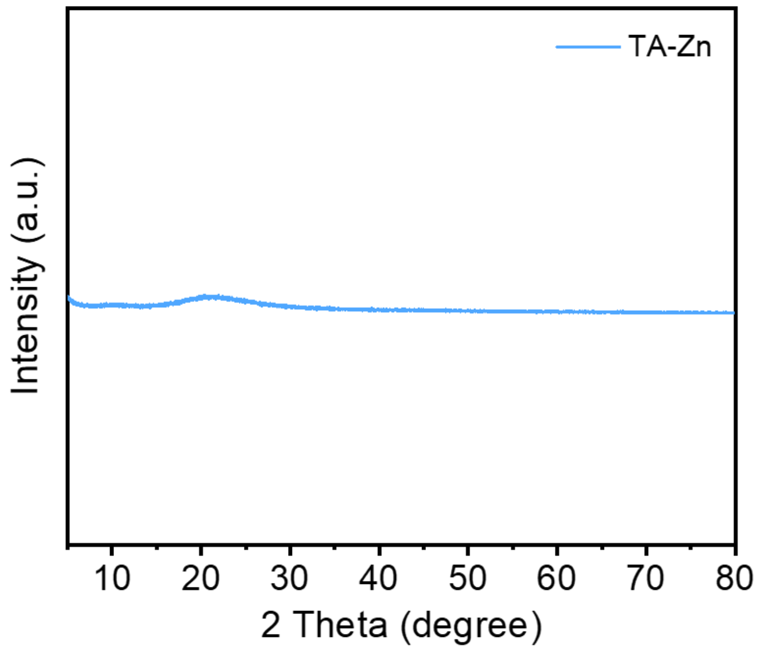

Figure S3. XRD pattern of the TA-Zn.

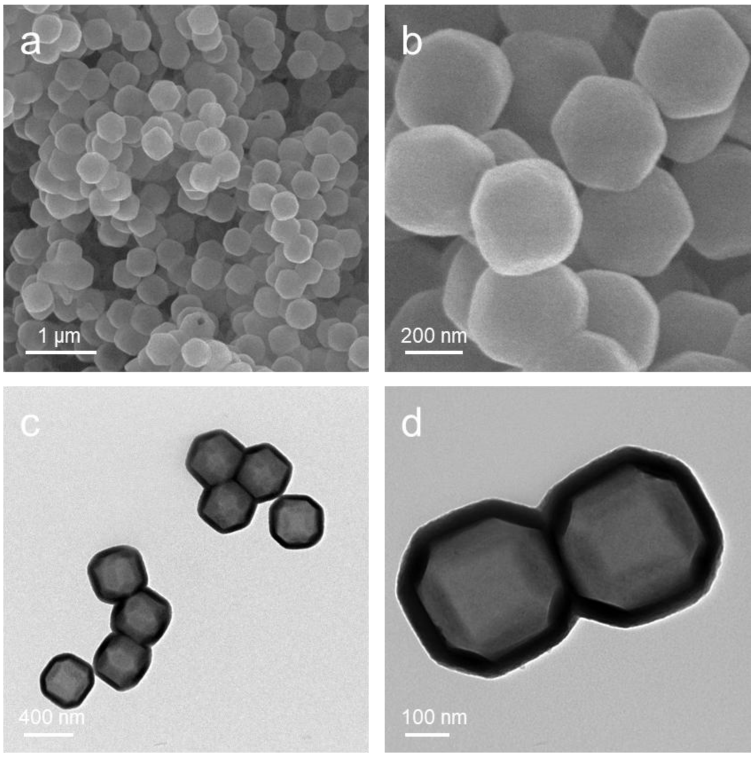

Figure S4. Low-magnification FESEM and TEM images of TA-Zn (a and c). High-magnification FESEM and TEM images of TA-Zn (b and d).

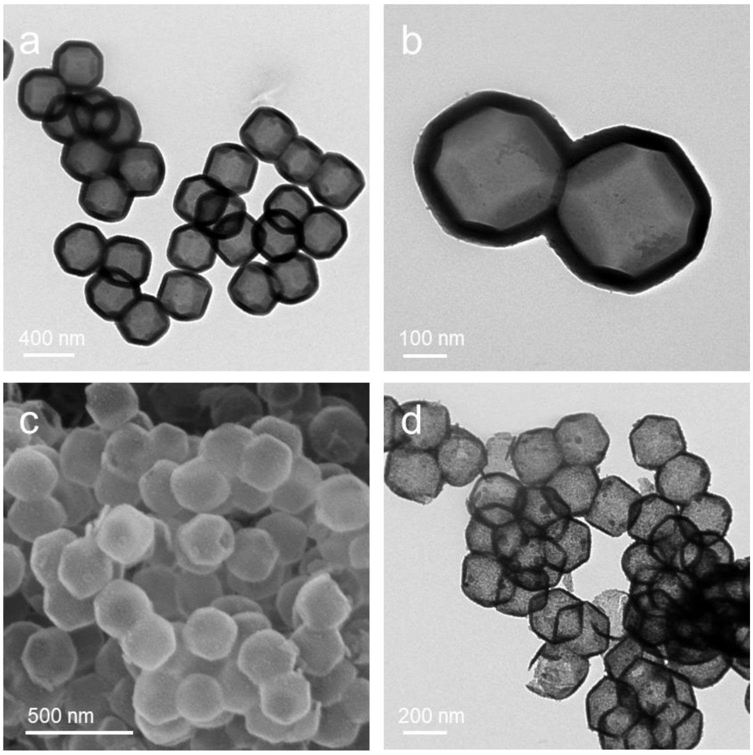

Figure S5. Low-magnification and high-magnification TEM images of TA-FeCoNiCuZn (a and b). Low-magnification FESEM and TEM images of FeCoNiCuZnO (c and d).

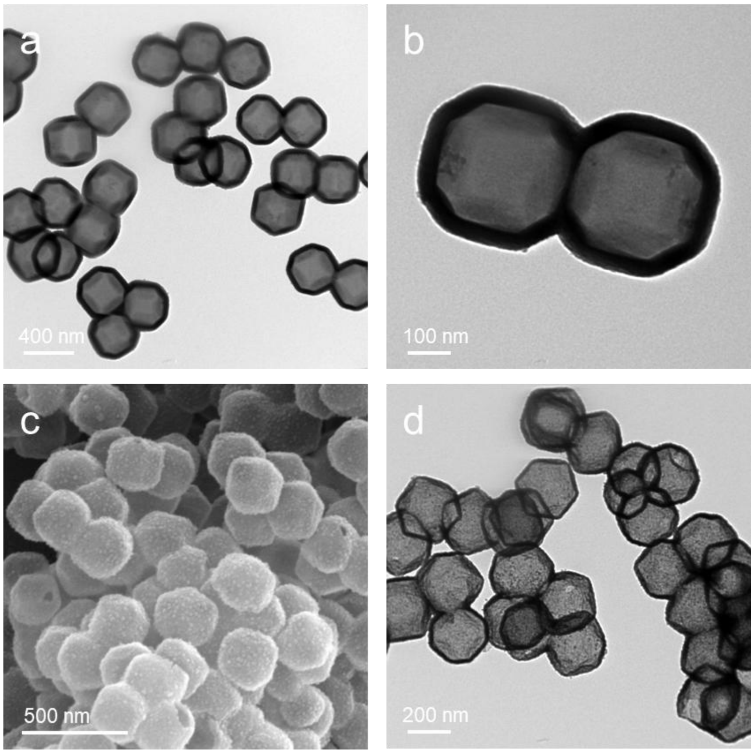

Figure S6. Low-magnification and high-magnification TEM images of TA-RuFeCoNiCuZn (a and b). Low-magnification FESEM and TEM images of RuFeCoNiCuZnO (c and d).

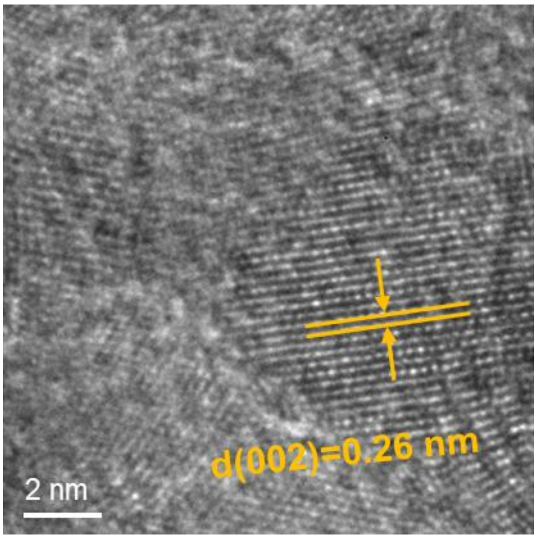

Figure S7. HRTEM image of FeCoNiCuZnO.

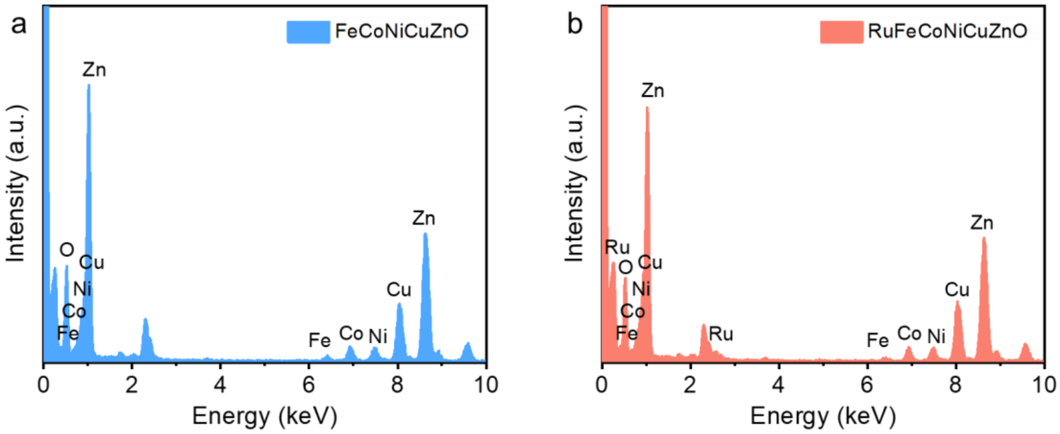

Figure S8. (a) EDS spectrum of the FeCoNiCuZnO (Mo mesh). (b) EDS spectrum of the RuFeCoNiCuZnO (Mo mesh).

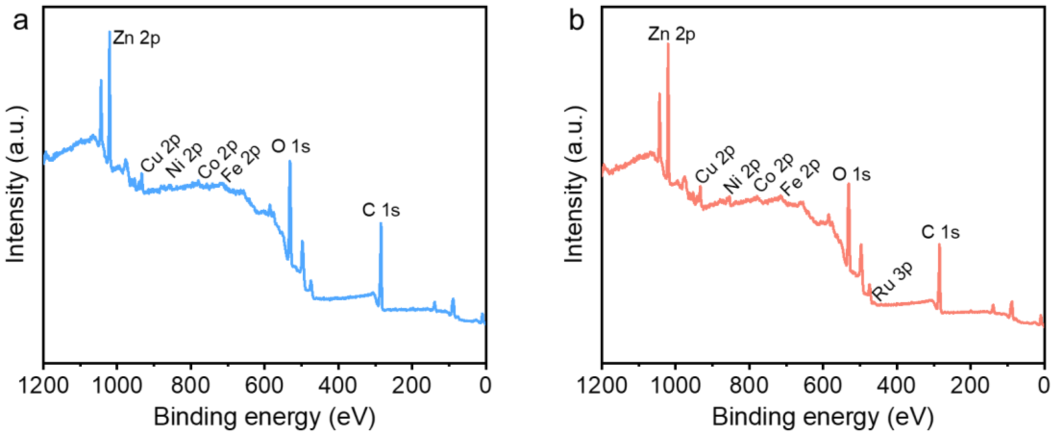

Figure S9. (a) XPS full spectra of the FeCoNiCuZnO. (b) XPS full spectra of the RuFeCoNiCuZnO.

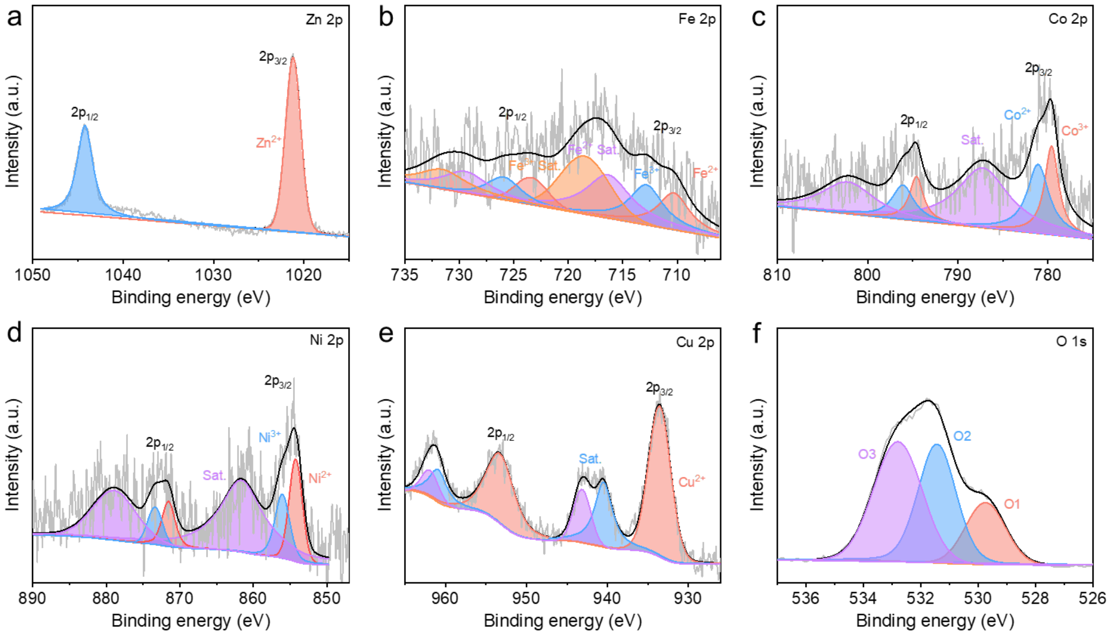

Figure S10. High-resolution XPS spectrum of (a) Zn 2p, (b) Fe 2p, (c) Co 2p, (d) Ni 2p, (e) Cu 2p, and (f) O 1s for FeCoNiCuZnO.

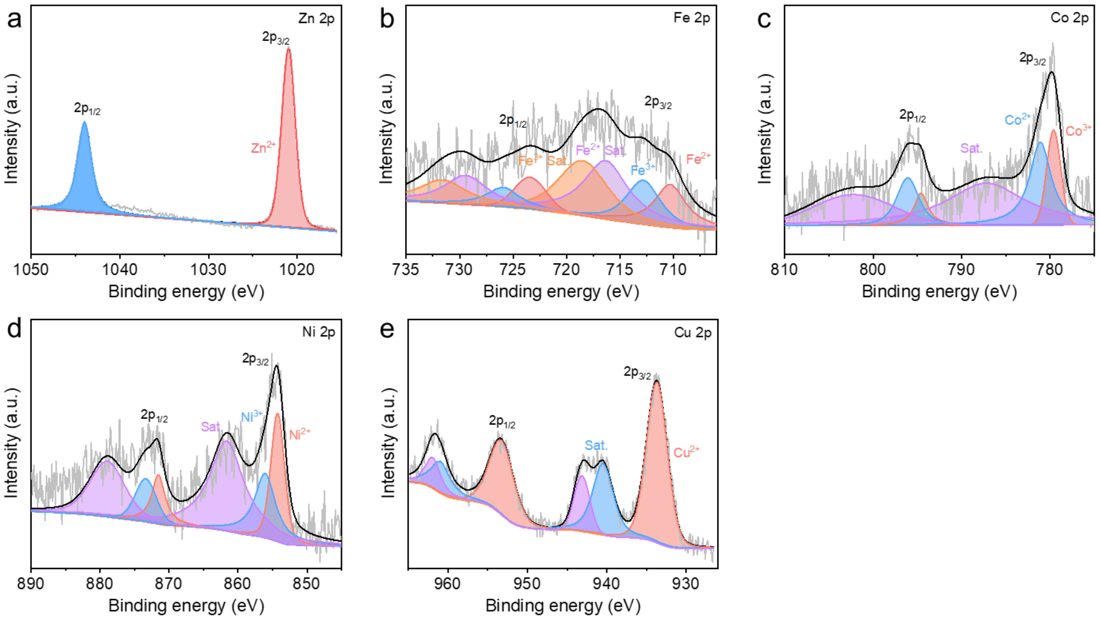

Figure S11. High-resolution XPS spectrum of (a) Zn 2p, (b) Fe 2p, (c) Co 2p, (d) Ni 2p, and (e) Cu 2p for RuFeCoNiCuZnO.

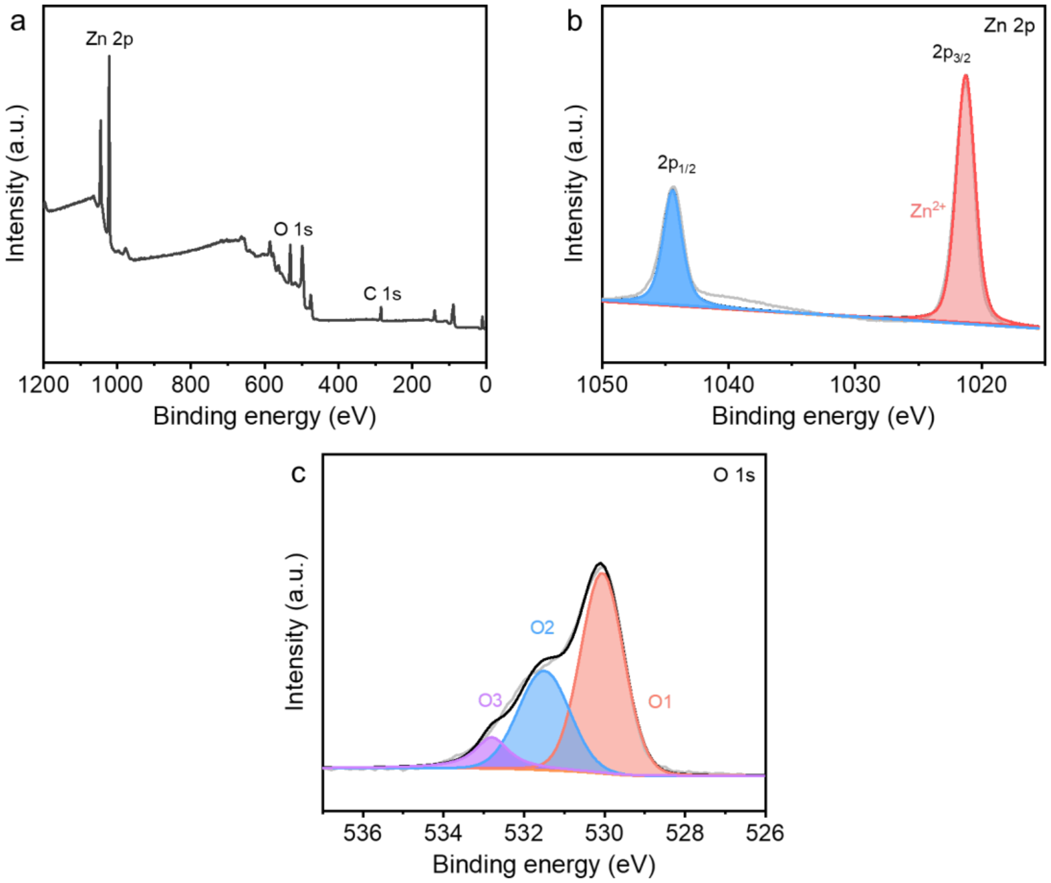

Figure S12. (a) XPS full spectra of the ZnO. High-resolution XPS spectrum of (b) Zn 2p and (c) O 1s for ZnO.

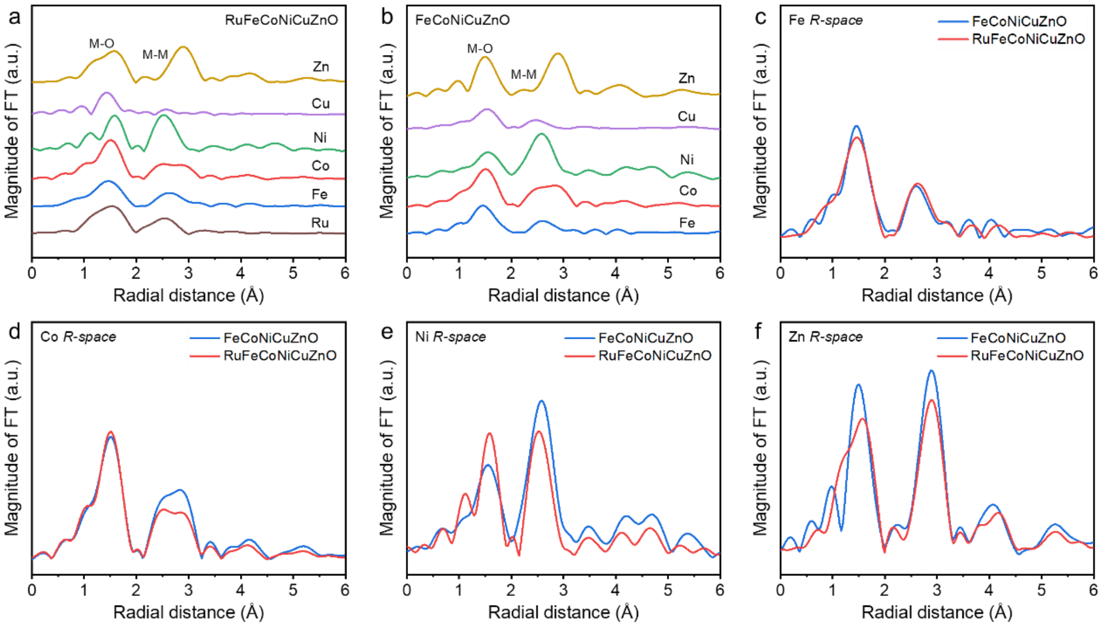

Figure S13. (a) The corresponding Fourier transform EXAFS spectra of the RuFeCoNiCuZnO at the corresponding metal K-edges. (b) The corresponding Fourier transform EXAFS spectra of the FeCoNiCuZnO at the corresponding metal K-edges. (c) The corresponding Fourier transform EXAFS spectra of the FeCoNiCuZnO and RuFeCoNiCuZnO at the Fe K-edge. (d) The corresponding Fourier transform EXAFS spectra of the FeCoNiCuZnO and RuFeCoNiCuZnO at the Co K-edge. (e) The corresponding Fourier transform EXAFS spectra of the FeCoNiCuZnO and RuFeCoNiCuZnO at the Ni K-edge. (f) The corresponding Fourier transform EXAFS spectra of the FeCoNiCuZnO and RuFeCoNiCuZnO at the Zn K-edge.

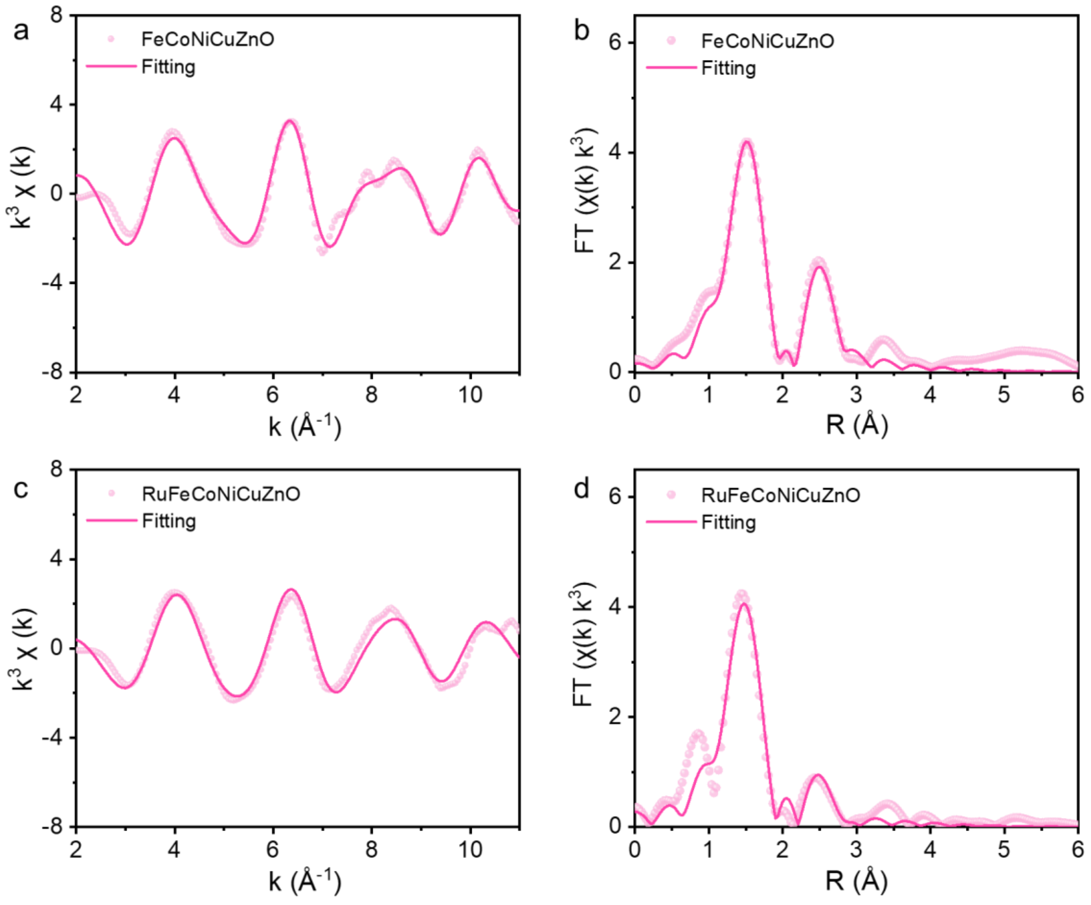

Figure S14. Cu K-edge EXAFS fitting results for (a and b) FeCoNiCuZnO and (c and d) RuFeCoNiCuZnO.

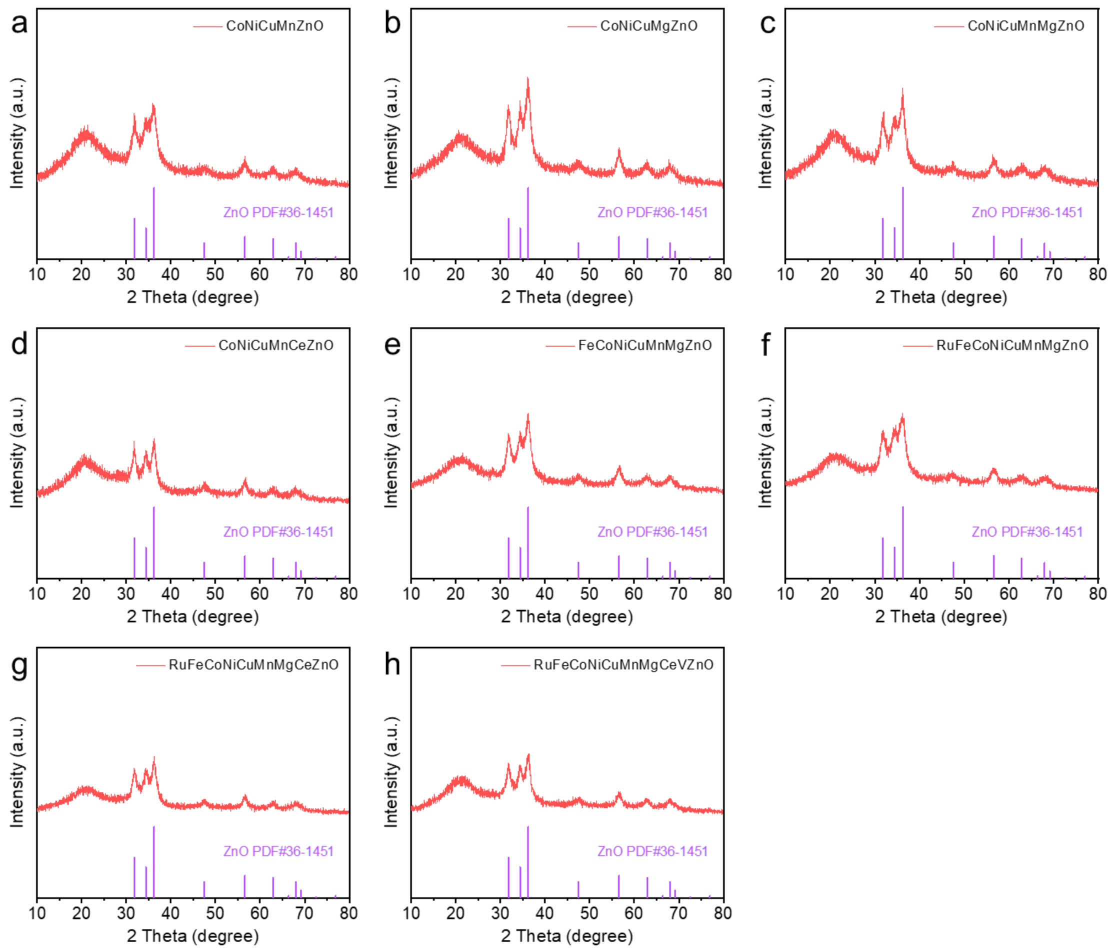

Figure S15. XRD patterns of the (a) CoNiCuMnZnO, (b) CoNiCuMgZnO, (c) CoNiCuMnMgZnO, (d)CoNiCuMnCeZnO, (e) FeCoNiCuMnMgZnO, (f) RuFeCoNiCuMnMgZnO, (g) RuFeCoNiCuMnMgCeZnO, and (h) RuFeCoNiCuMnMgCeVZnO.

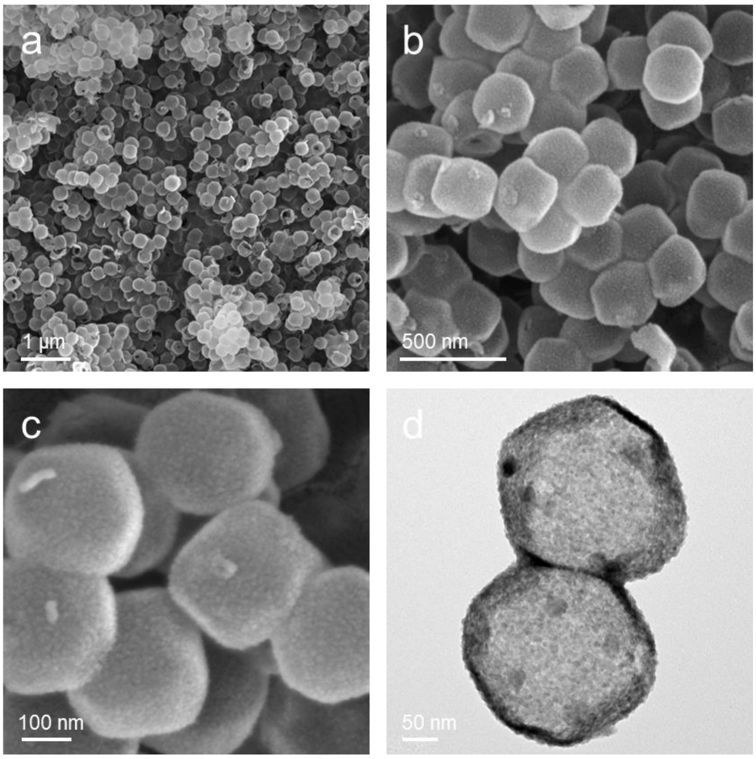

Figure S16. Low-magnification and high-magnification FESEM images of CoNiCuMnZnO (a, b, and c). (d) High-magnification TEM image of CoNiCuMnZnO.

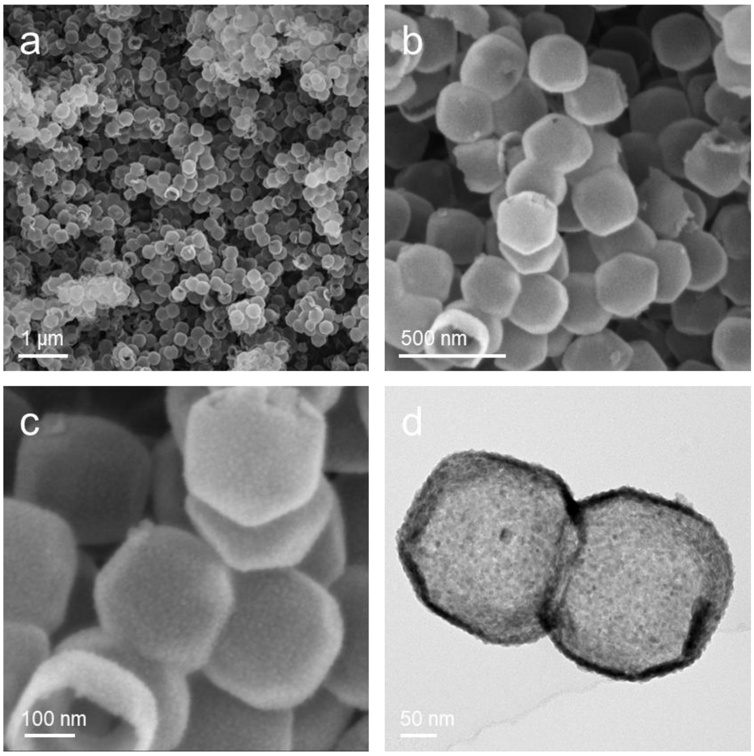

Figure S17. Low-magnification and high-magnification FESEM images of CoNiCuMgZnO (a, b, and c). (d) High-magnification TEM image of CoNiCuMgZnO.

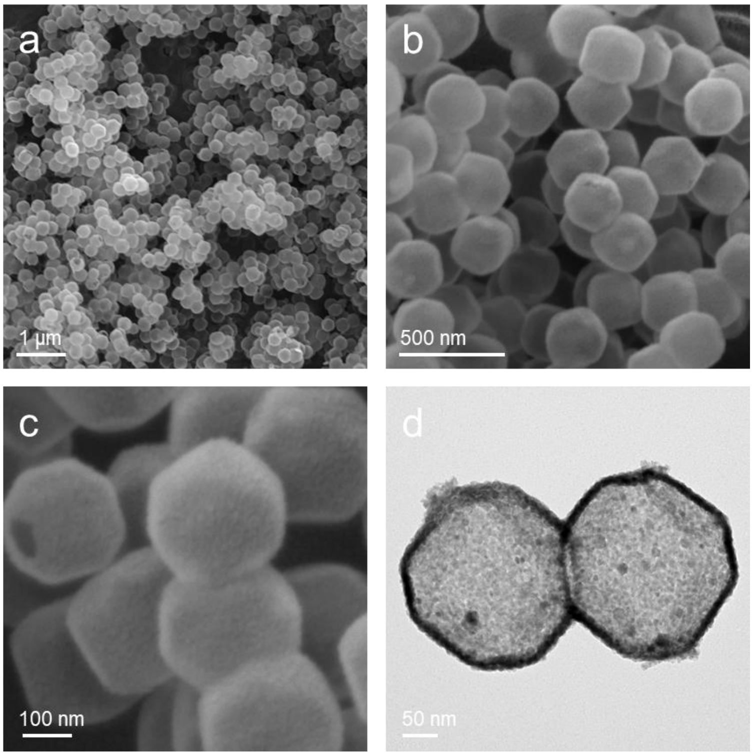

Figure S18. Low-magnification and high-magnification FESEM images of CoNiCuMnMgZnO (a, b, and c). (d) High-magnification TEM image of CoNiCuMnMgZnO.

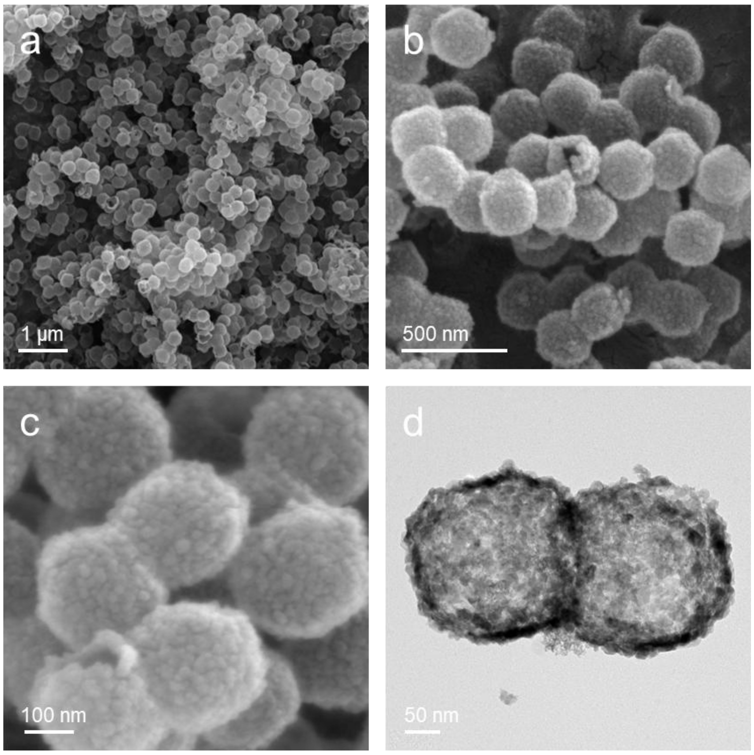

Figure S19. Low-magnification and high-magnification FESEM images of CoNiCuMnCeZnO (a, b, and c). (d) High-magnification TEM image of CoNiCuMnCeZnO.

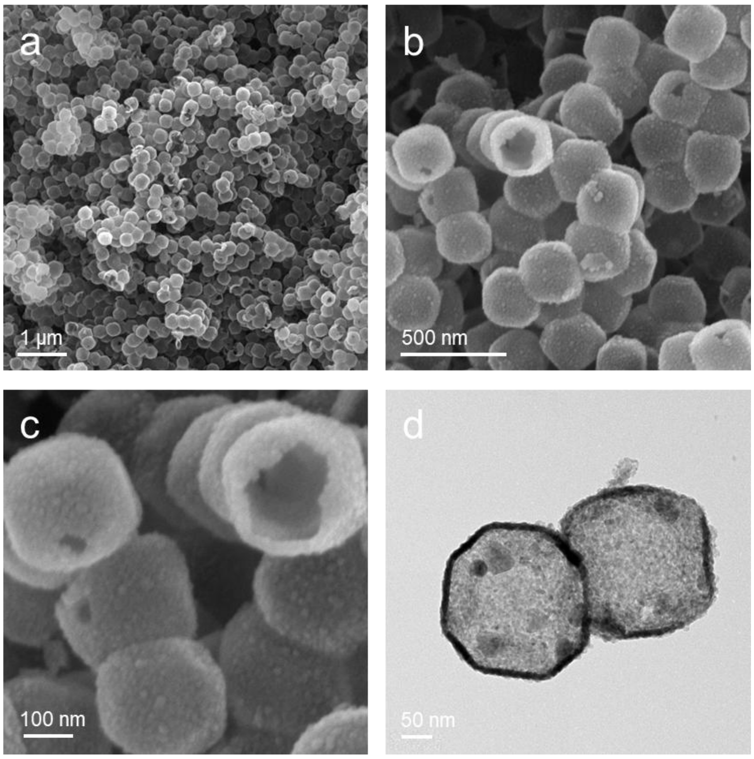

Figure S20. Low-magnification and high-magnification FESEM images of FeCoNiCuMnMgZnO (a, b, and c). (d) High-magnification TEM image of FeCoNiCuMnMgZnO.

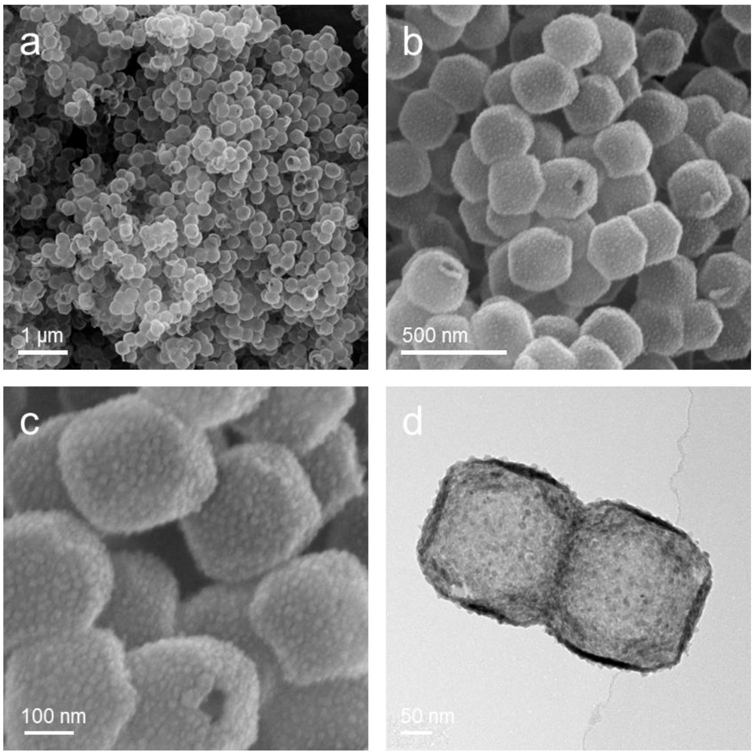

Figure S21. Low-magnification and high-magnification FESEM images of RuFeCoNiCuMnMgZnO (a, b, and c). (d) High-magnification TEM image of RuFeCoNiCuMnMgZnO.

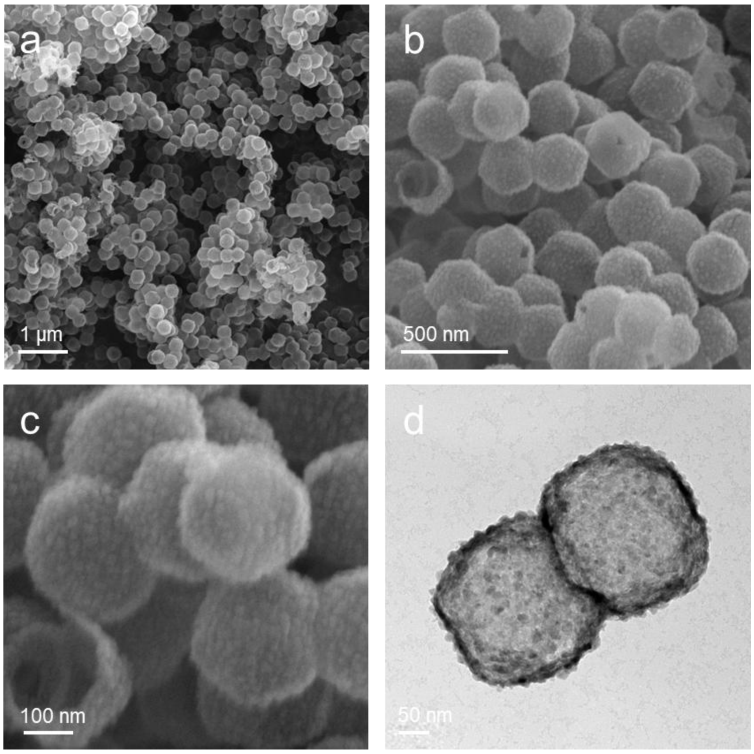

Figure S22. Low-magnification and high-magnification FESEM images of RuFeCoNiCuMnMgCeZnO (a, b, and c). (d) High-magnification TEM image of RuFeCoNiCuMnMgCeZnO.

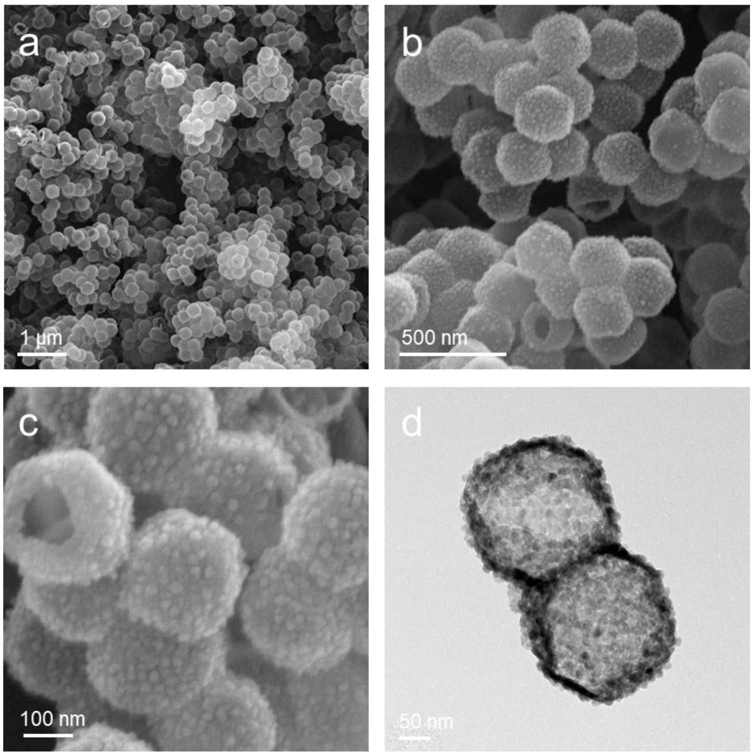

Figure S23. Low-magnification and high-magnification FESEM images of RuFeCoNiCuMnMgCeVZnO (a, b, and c). (d) High-magnification TEM image of RuFeCoNiCuMnMgCeVZnO.

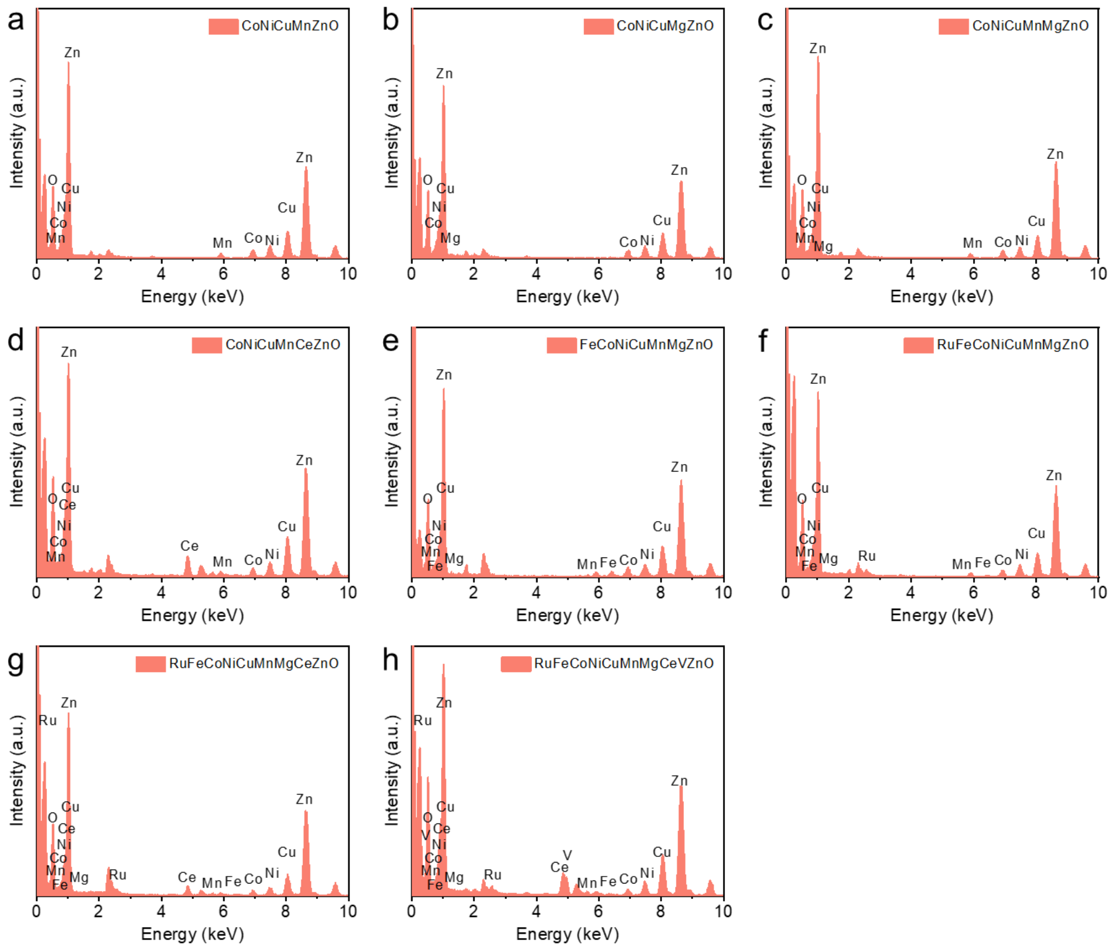

Figure S24. EDS spectrum of the (a) CoNiCuMnZnO, (b) CoNiCuMgZnO, (c) CoNiCuMnMgZnO, (d)CoNiCuMnCeZnO, (e) FeCoNiCuMnMgZnO, (f) RuFeCoNiCuMnMgZnO, (g) RuFeCoNiCuMnMgCeZnO, and (h) RuFeCoNiCuMnMgCeVZnO (Mo mesh).

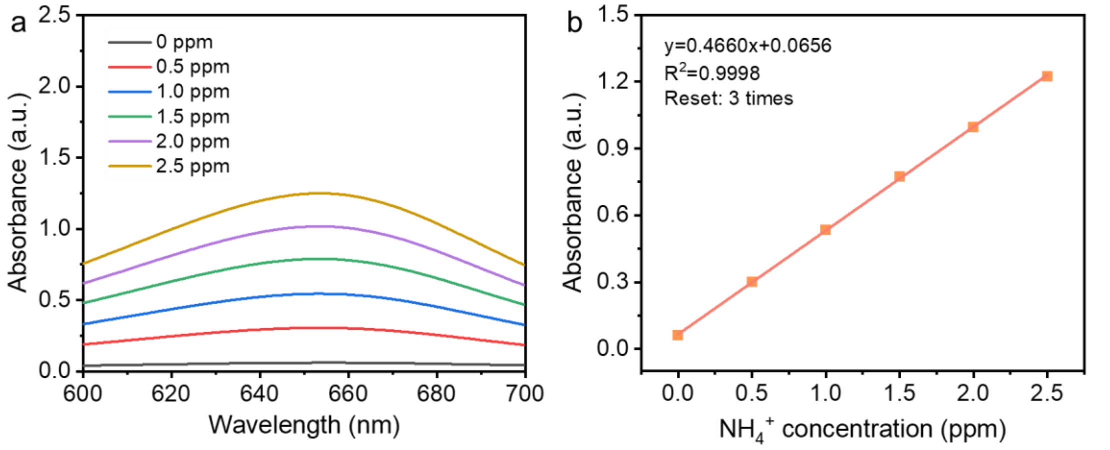

Figure S25. (a) UV-Vis absorption spectra of indophenol assays with NH_4^+ after incubated for 2 h at room temperature. (b) Linear fitting of the corresponding calibration curve for estimation of NH_4^+ concentration.

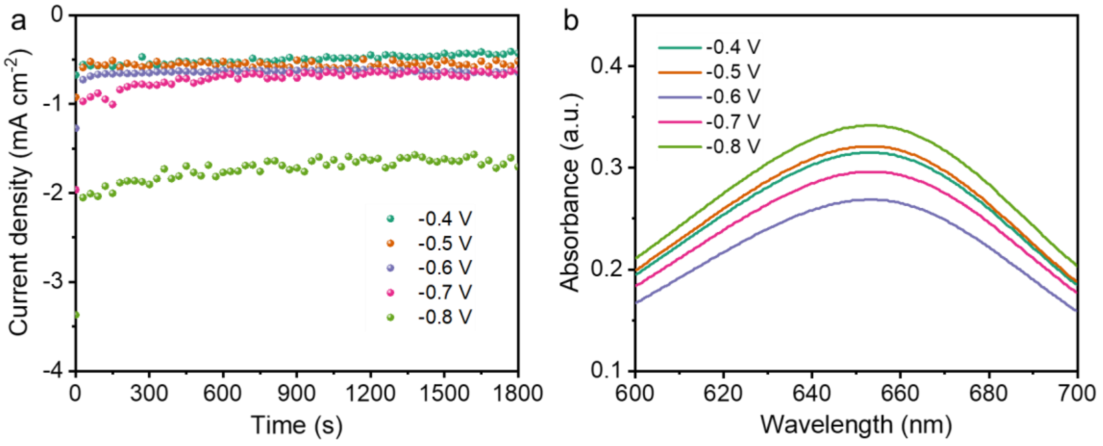

Figure S26. (a) i-t curves for electrocatalytic NH_3 synthesis at various applied potential on RuFeCoNiCuZnO. (b) UV-Vis absorption spectra of corresponding electrolyte.

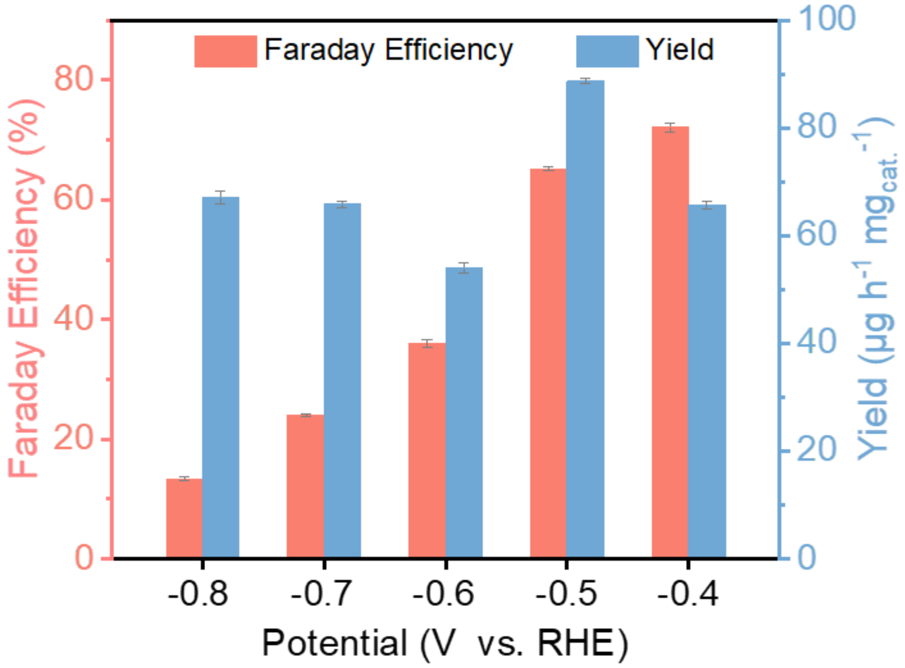

Figure S27. Faraday efficiency and yield rate of NH_3 over the FeCoNiCuZnO at different potentials.

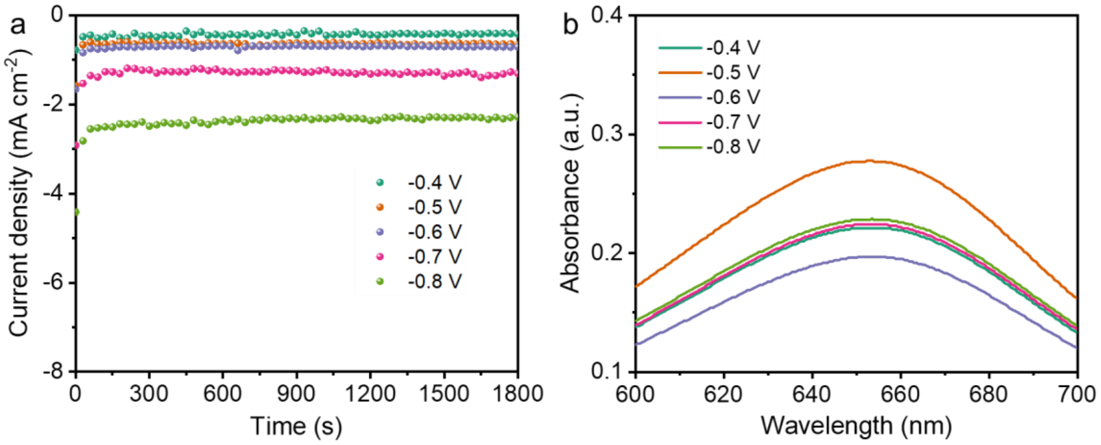

Figure S28. (a) i-t curves for electrocatalytic NH_3 synthesis at various applied potential on FeCoNiCuZnO. (b) UV-Vis absorption spectra of corresponding electrolyte.

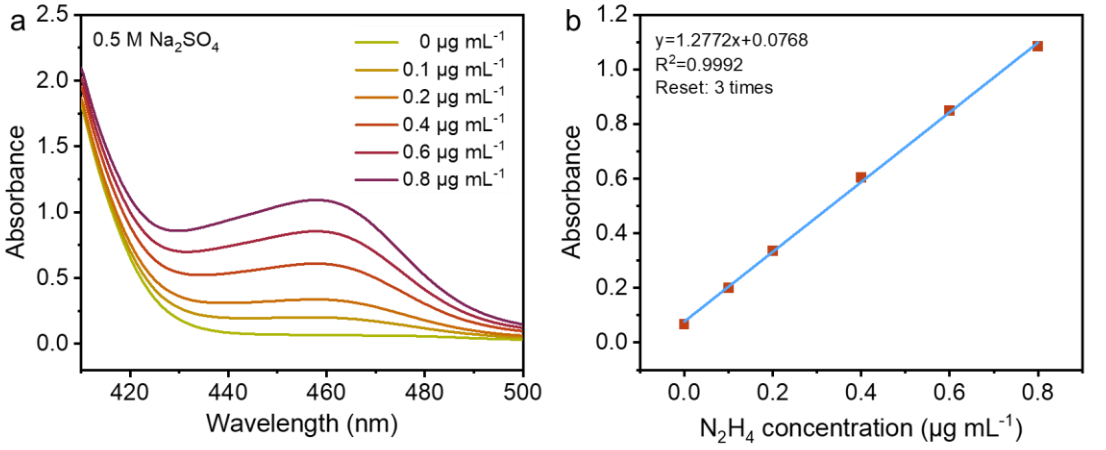

Figure S29. (a) UV-Vis absorption spectra of various N_2H_4 concentrations after incubated for 10 min at room temperature. (b) Linear fitting of the corresponding calibration curve for estimation of N_2H_4 concentration.

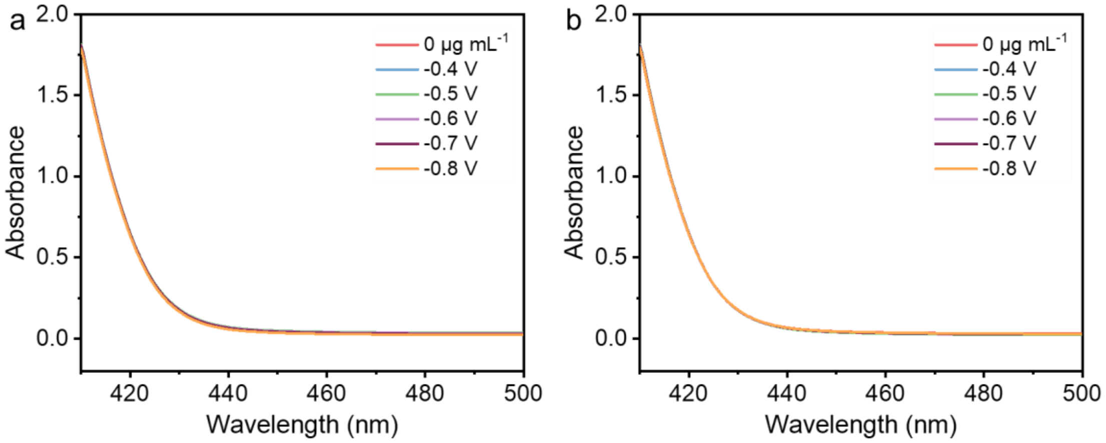

Figure S30. UV-Vis absorption spectra of corresponding electrolyte at various applied potential on (a) FeCoNiCuZnO and (b) RuFeCoNiCuZnO.

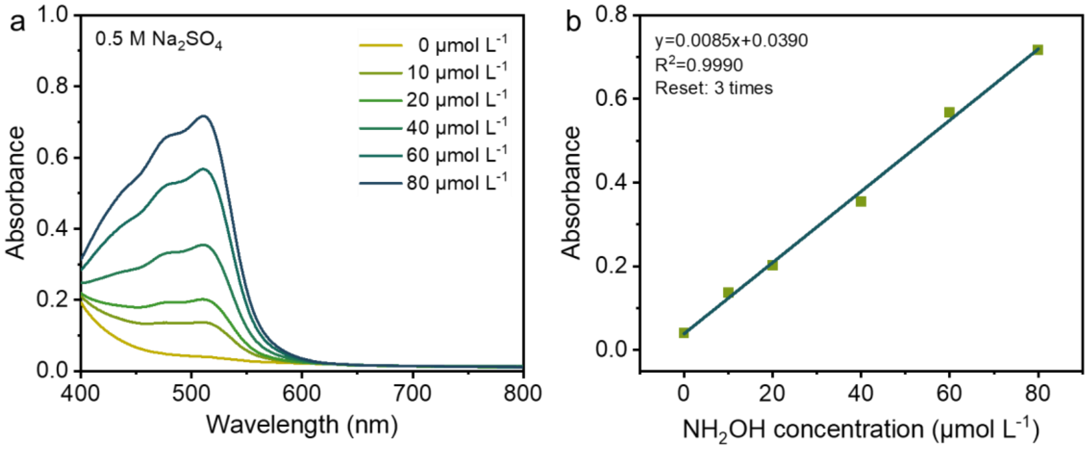

Figure S31. (a) UV-Vis absorption spectra of various NH_2OH concentrations after incubated for 30 min at room temperature. (b) Linear fitting of the corresponding calibration curve for estimation of NH_2OH concentration.

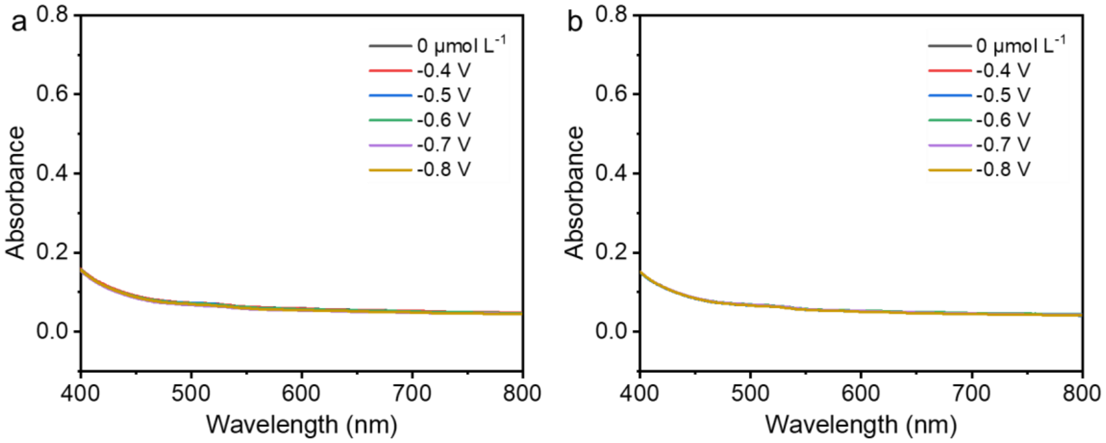

Figure S32. UV-Vis absorption spectra of corresponding electrolyte at various applied potential on (a) FeCoNiCuZnO and (b) RuFeCoNiCuZnO.

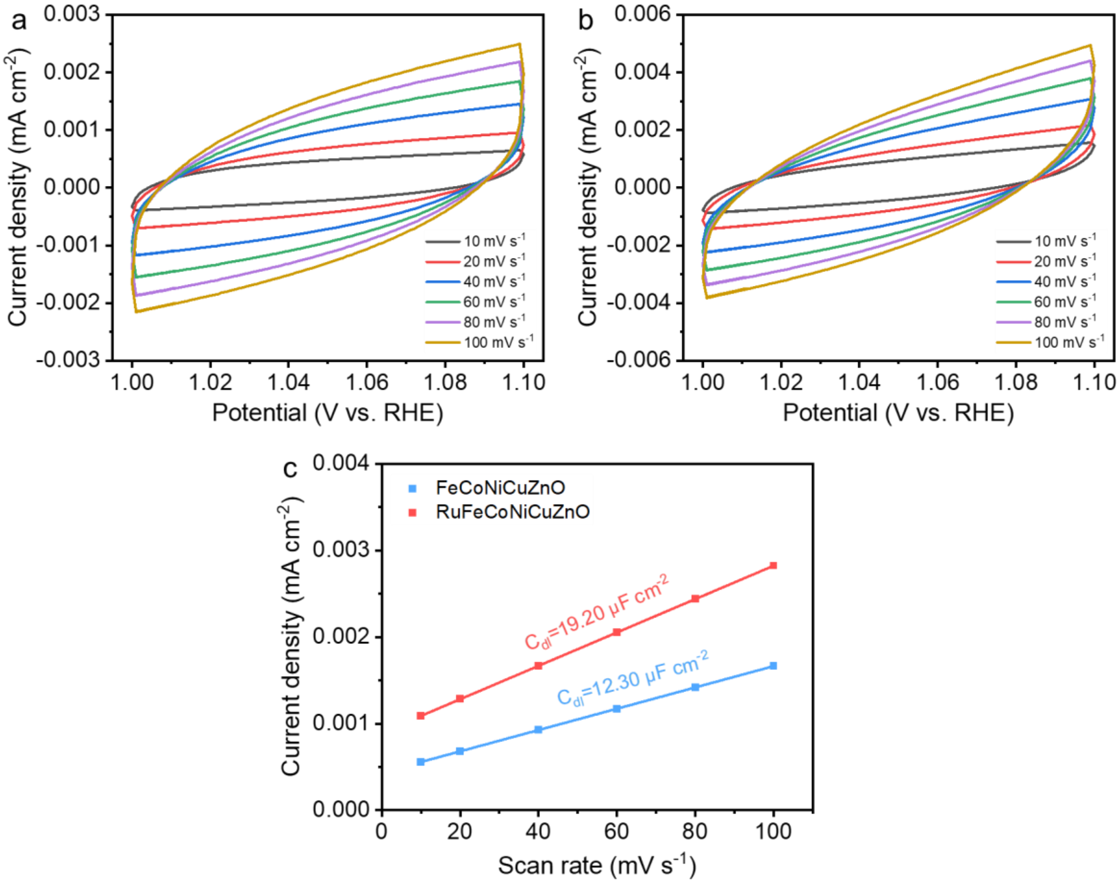

Figure S33. (a) CV curves at different scan rates for FeCoNiCuZnO. (b) CV curves at different scan rates for RuFeCoNiCuZnO. (c) Linear fitting of the capacitive currents versus CV scan rates for FeCoNiCuZnO and RuFeCoNiCuZnO.

The ECSA of catalysts is calculated by the following equation: ECSA = C_{dl} / C_s. Where C_s is the specific capacitance of planar surface with an atomically smooth under identical electrolyte conditions, 40 μF cm^{-2} is used in this work. The ECSA of FeCoNiCuZnO and RuFeCoNiCuZnO is 0.31 and 0.48 cm^2, respectively.

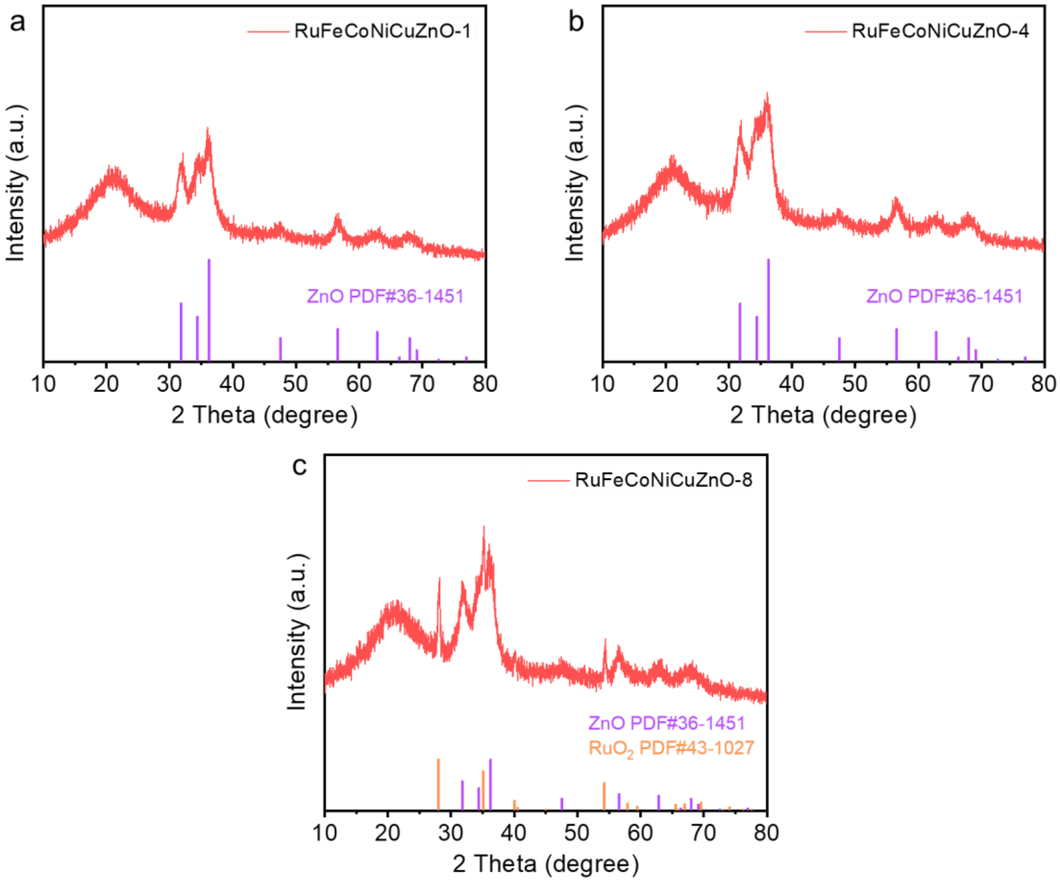

Figure S34. (a) XRD pattern of the RuFeCoNiCuZnO-1. (b) XRD pattern of the RuFeCoNiCuZnO-4. (c) XRD pattern of the RuFeCoNiCuZnO-8.

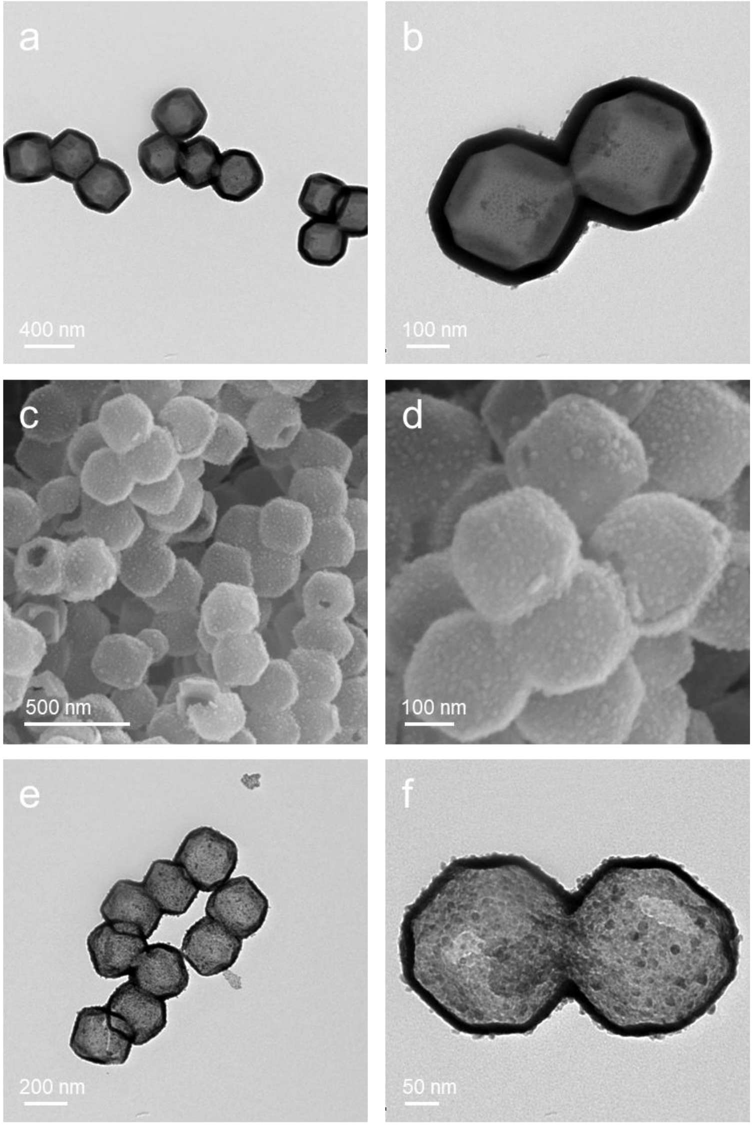

Figure S35. Low-magnification and high-magnification TEM images of TA-RuFeCoNiCuZn-1 (a and b). Low-magnification FESEM and TEM images of RuFeCoNiCuZnO-1 (c and e). High-magnification FESEM and TEM images of RuFeCoNiCuZnO-1 (d and f).

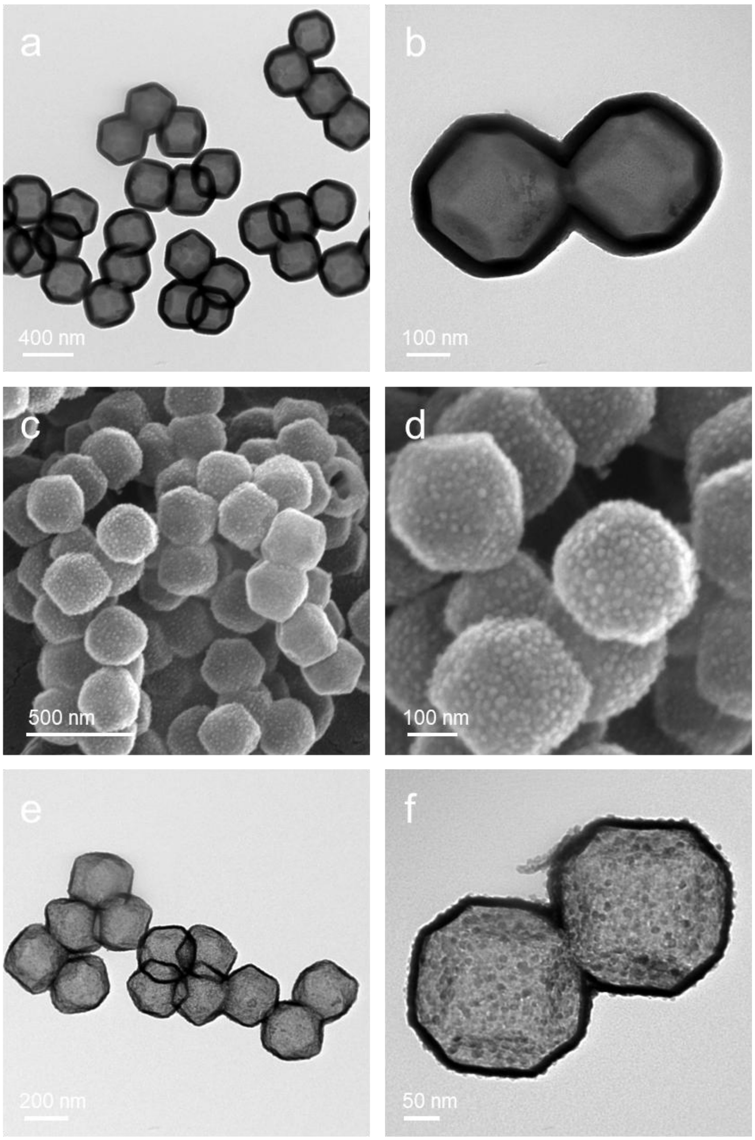

Figure S36. Low-magnification and high-magnification TEM images of TA-RuFeCoNiCuZn-4 (a and b). Low-magnification FESEM and TEM images of RuFeCoNiCuZnO-4 (c and e). High-magnification FESEM and TEM images of RuFeCoNiCuZnO-4 (d and f).

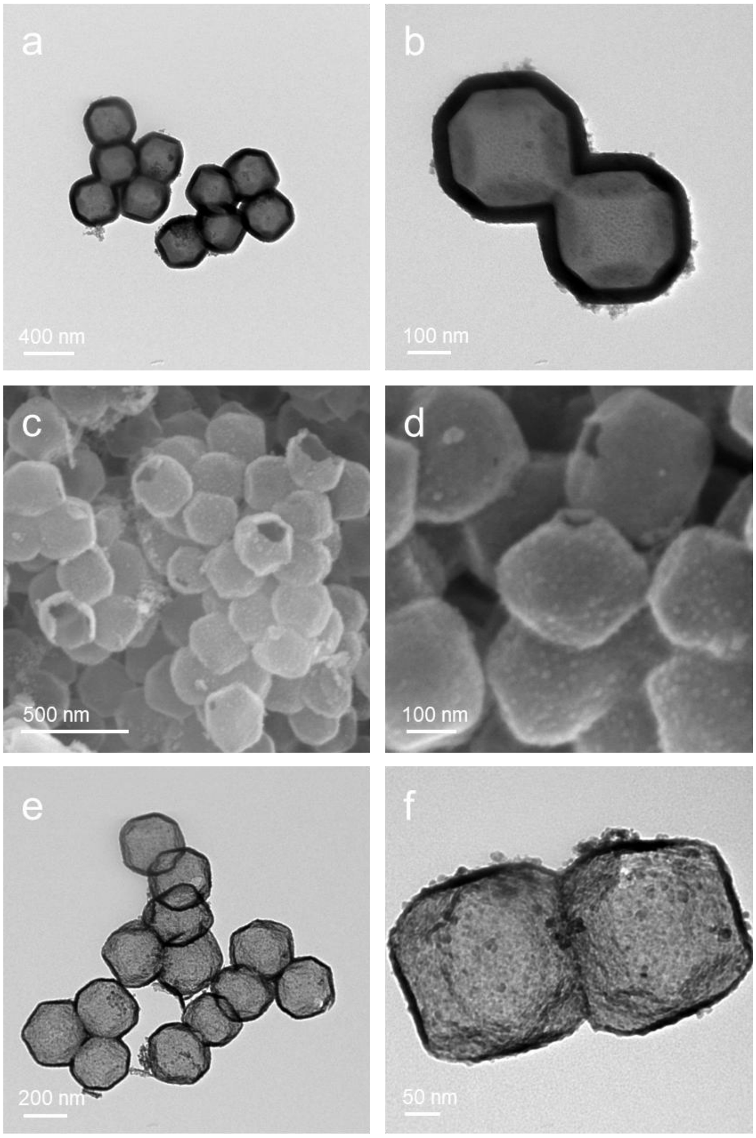

Figure S37. Low-magnification and high-magnification TEM images of TA-RuFeCoNiCuZn-8 (a and b). Low-magnification FESEM and TEM images of RuFeCoNiCuZnO-8 (c and e). High-magnification FESEM and TEM images of RuFeCoNiCuZnO-8 (d and f).

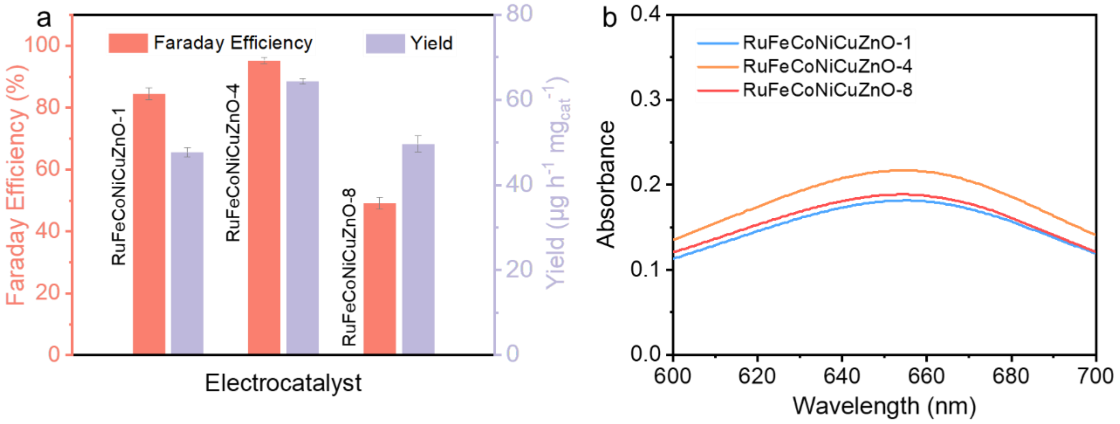

Figure S38. (a) Faraday efficiency and yield rate of NH_3 over the RuFeCoNiCuZnO-1, RuFeCoNiCuZnO-4, and RuFeCoNiCuZnO-8 at -0.4 V vs. RHE. (b) UV-Vis absorption spectra of corresponding electrolytes.

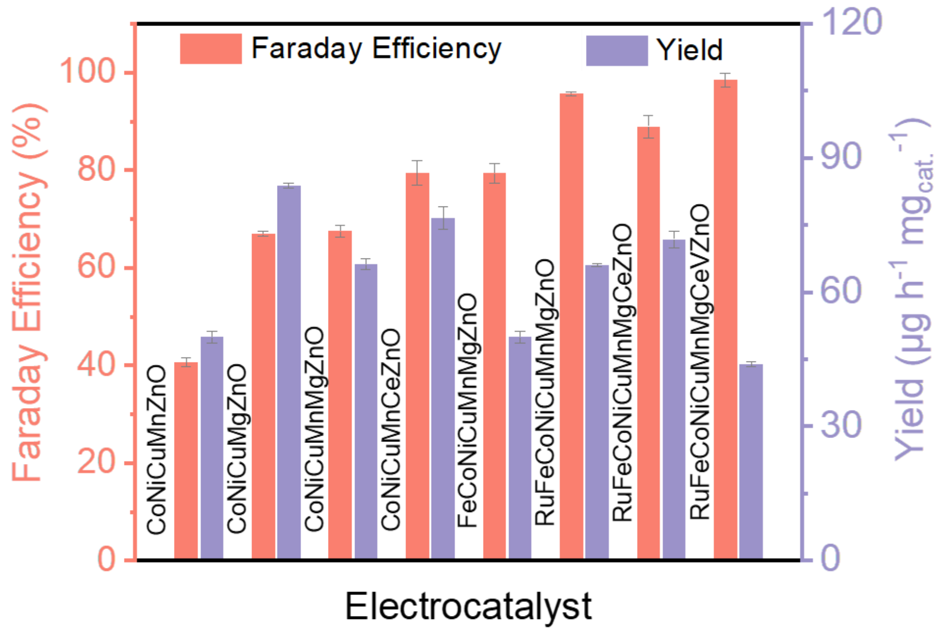

Figure S39. Faraday efficiency and yield rate of NH_3 over the CoNiCuMnZnO, CoNiCuMgZnO, CoNiCuMnMgZnO, CoNiCuMnCeZnO, FeCoNiCuMnMgZnO, RuFeCoNiCuMnMgZnO, RuFeCoNiCuMnMgCeZnO, and RuFeCoNiCuMnMgCeVZnO at -0.4 V vs. RHE.

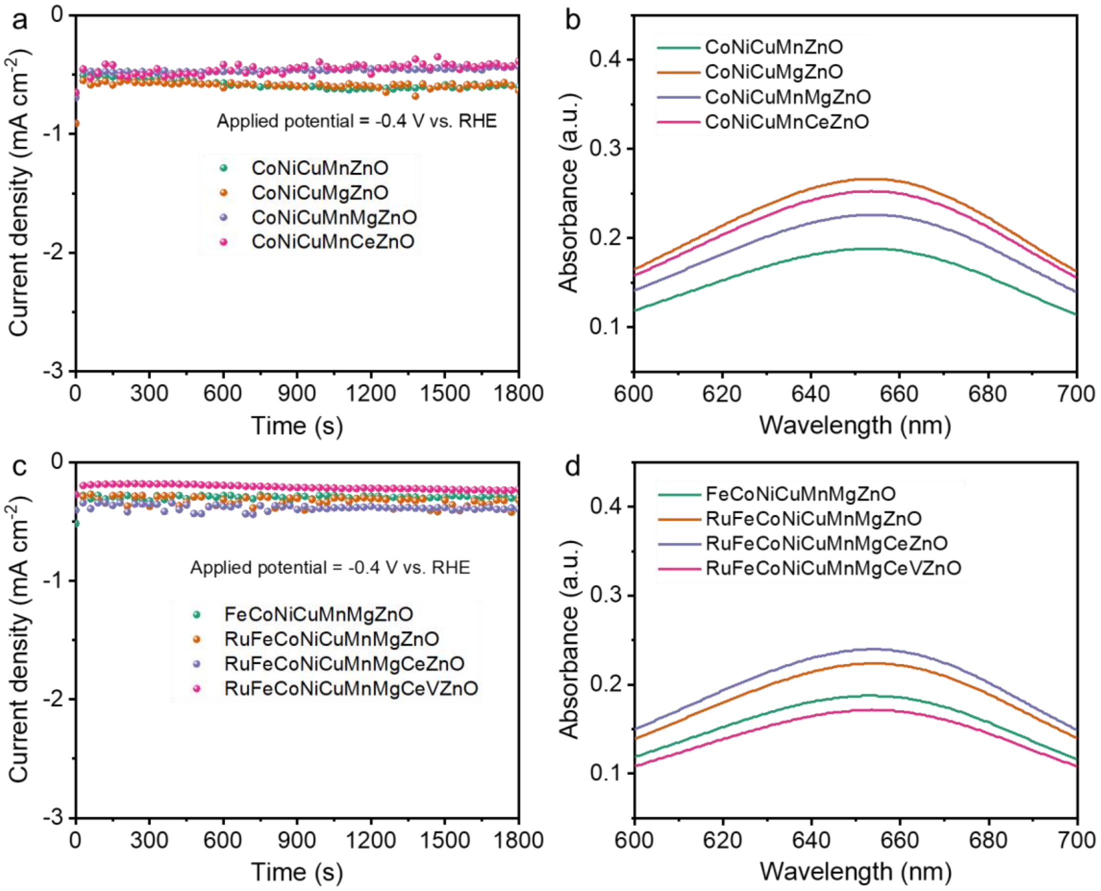

Figure S40. (a) i-t curves for electrocatalytic NH_3 synthesis at -0.4 V vs. RHE on CoNiCuMnZnO, CoNiCuMgZnO, CoNiCuMnMgZnO, and CoNiCuMnCeZnO. (b) UV-Vis absorption spectra of corresponding electrolyte. (c) i-t curves for electrocatalytic NH_3 synthesis at -0.4 V vs. RHE on FeCoNiCuMnMgZnO, RuFeCoNiCuMnMgZnO, RuFeCoNiCuMnMgCeZnO, and RuFeCoNiCuMnMgCeVZnO. (d) UV-Vis absorption spectra of corresponding electrolyte.

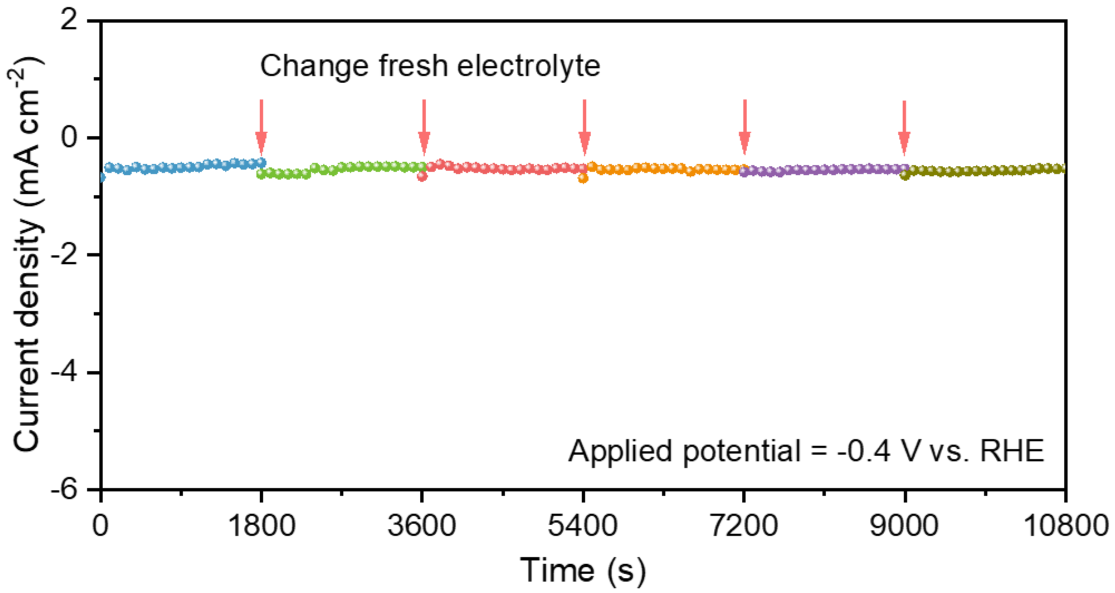

Figure S41. Long-term chronoamperometry on RuFeCoNiCuZnO for NORR at -0.4 V vs. RHE by replacing new electrolyte.

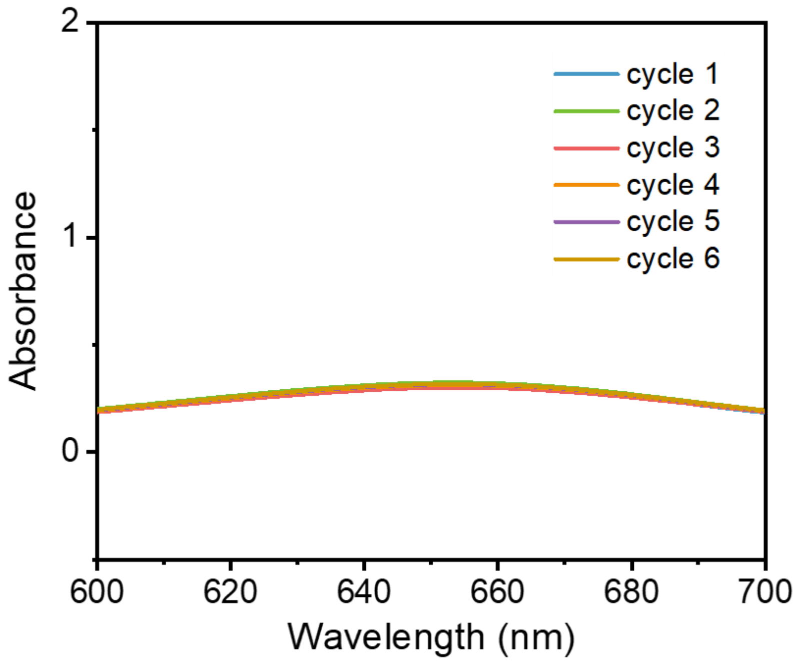

Figure S42. UV-Vis absorption spectra of the electrolyte in six cycles over RuFeCoNiCuZnO for NORR at -0.4 V vs. RHE.

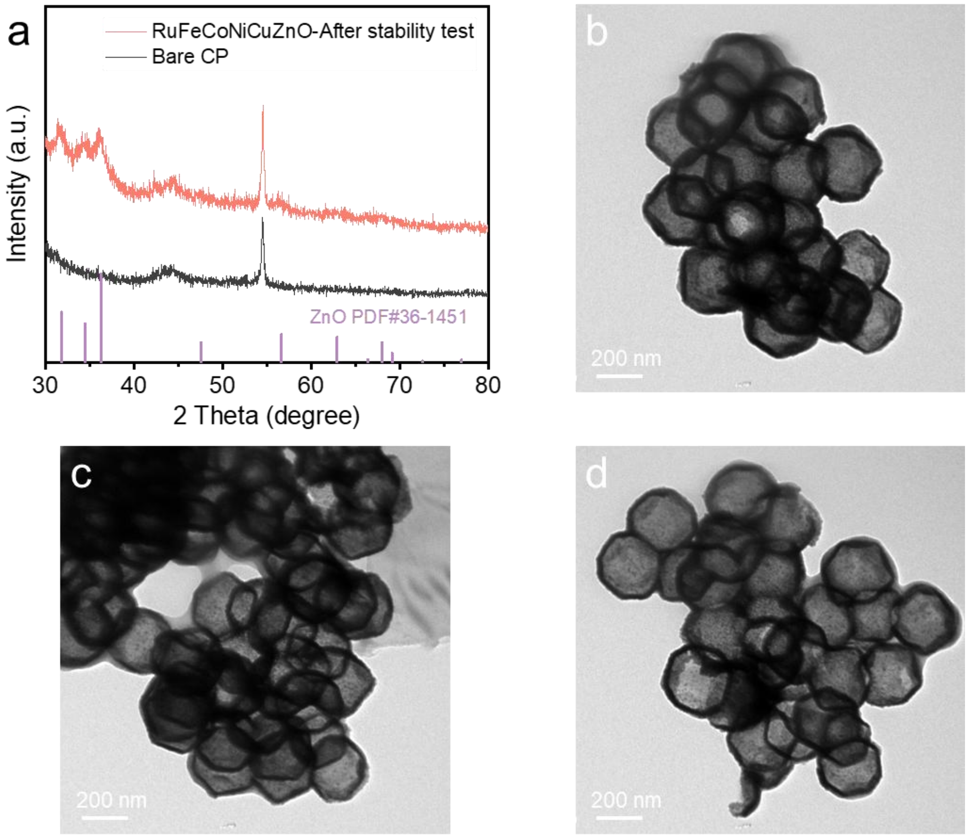

Figure S43. (a) XRD patterns of the RuFeCoNiCuZnO after stability test and bare CP (Note: the peak at around 2 theta = 55 degree originates from the purchased commercial CP). (b, c, and d) TEM images of the RuFeCoNiCuZnO at different positions after stability test (Note: nafion serves as an interfacial binder between adjacent polyhedral units).

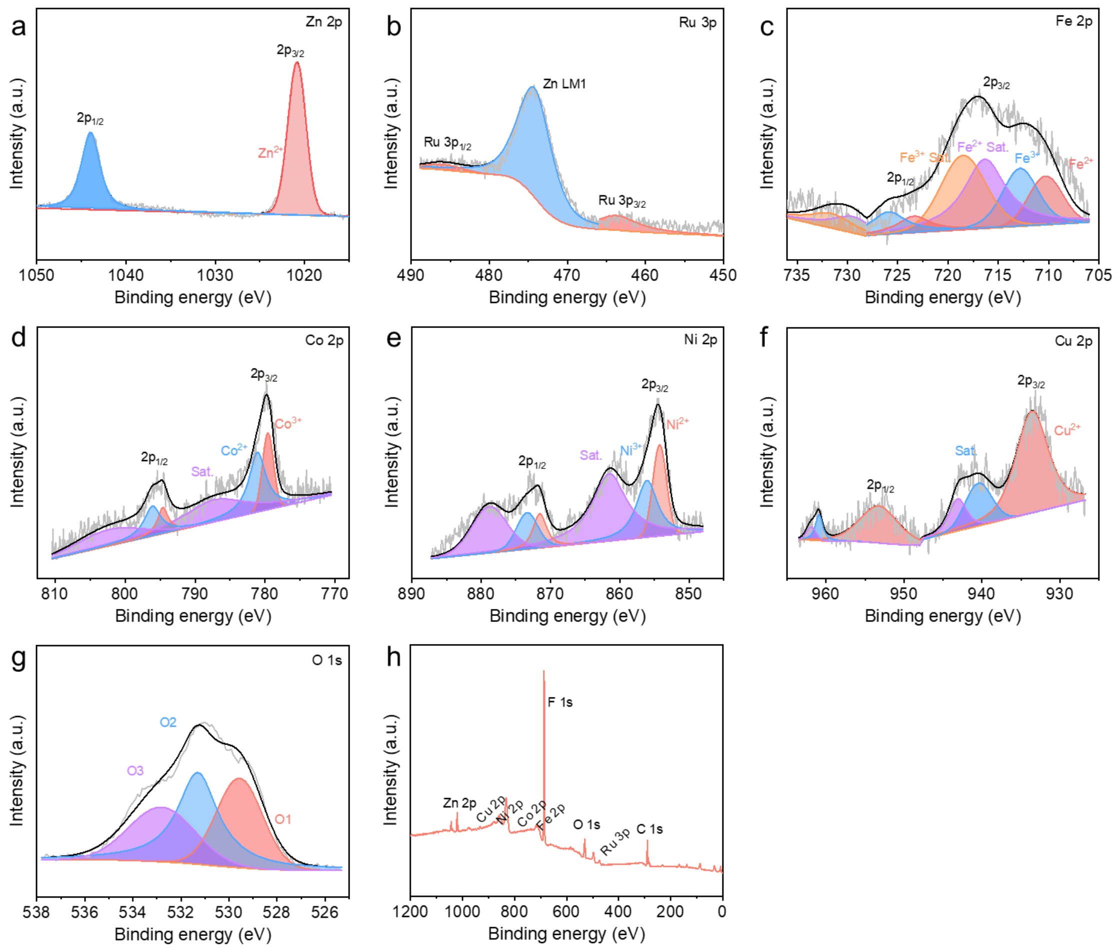

Figure S44. High-resolution XPS spectrum of (a) Zn 2p, (b) Ru 3p, (c) Fe 2p, (d) Co 2p, (e) Ni 2p, (f) Cu 2p, and (g) O 1s for RuFeCoNiCuZnO after stability test. (h) XPS full spectra of RuFeCoNiCuZnO after stability test (Note: F 1s signal originates from nafion binder).

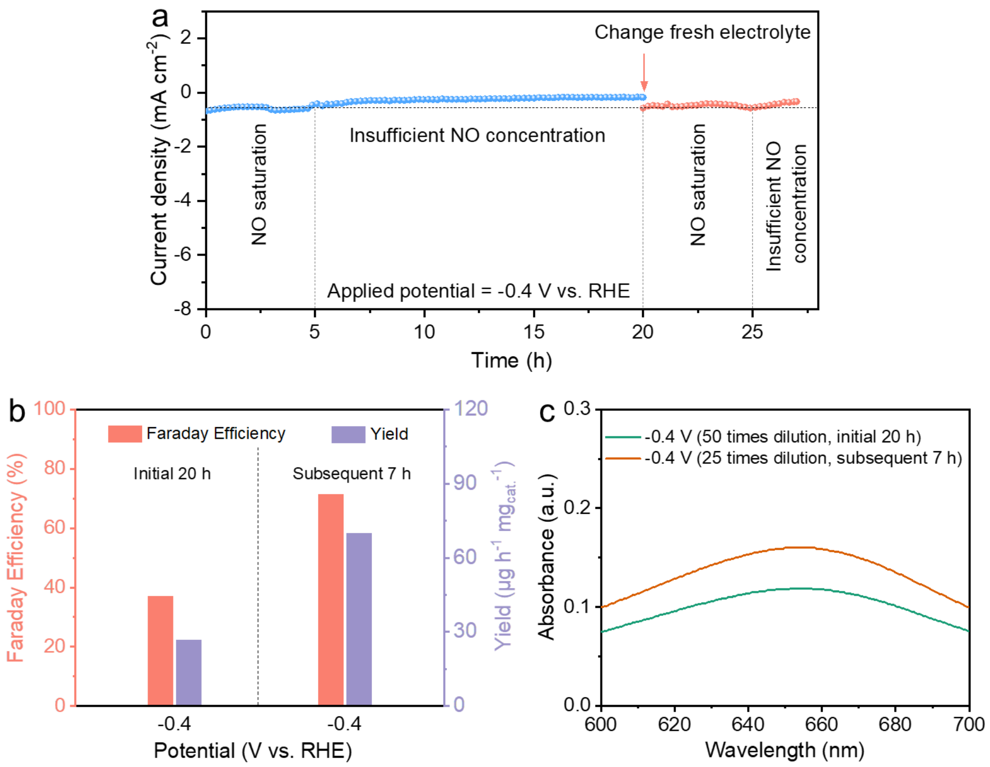

Figure S45. (a) NORR stability testing on RuFeCoNiCuZnO at -0.4 V vs. RHE. (b) Faraday efficiency and yield rate of NH_3 over the RuFeCoNiCuZnO after stability test. (c) UV-Vis absorption spectra of corresponding electrolyte.

During the 27-h stability test, the current density remains relatively stable for the initial 5 h. From the 5th to the 20th h, a gradual decrease is observed until a new steady state is reached. After electrolyte refresh, an additional 7-h test displays a similar pattern: the current density retention exceeds 90% and remains stable for the first 5 h, followed by a subsequent gradual decline. During the initial 5-h period, the electrolyte is saturated with NO, which maintains a stable current density. After this period, the gradual decline in current is attributed to the consumption of dissolved NO in the electrolyte (NO is slightly soluble in water: 0.0056 g/100 mL at 20 °C). When the NO supply and consumption reach a new equilibrium, the current density stabilizes again at a lower steady state. Concurrently, the slight structural degradation of a minority fraction of the catalyst during extended operation serves as another potential factor contributing to the observed decline in current density retention (Figure S46).

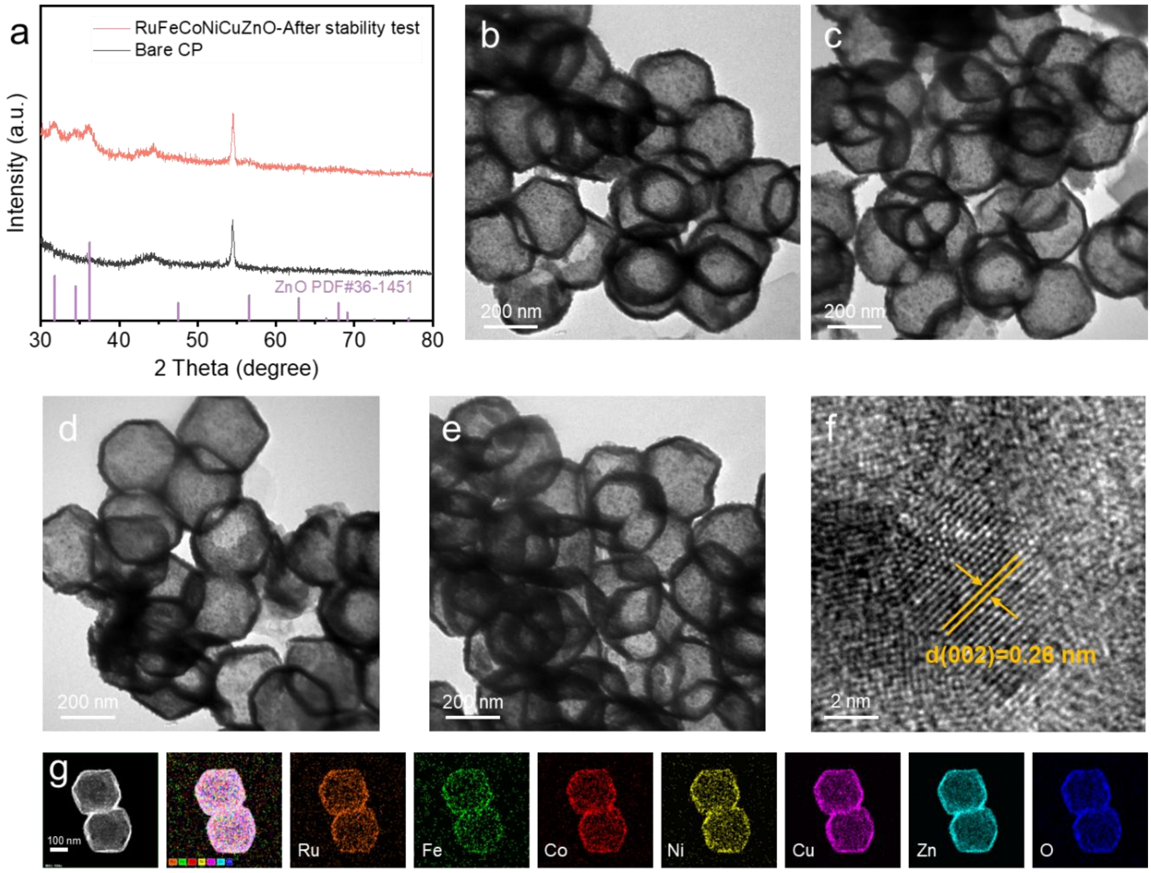

Figure S46. (a) XRD patterns of the RuFeCoNiCuZnO after the 27-h stability test and bare CP (Note: the peak at around 2 theta = 55 degree originates from the purchased commercial CP). (b, c, d, and e) TEM images of the RuFeCoNiCuZnO at different positions after 27 h stability (Note: nafion serves as an interfacial binder between adjacent polyhedral units). (f) HRTEM image of the RuFeCoNiCuZnO after the 27-h stability test. (g) HAADF-STEM image and the corresponding elemental mapping images of RuFeCoNiCuZnO after the 27-h stability test.

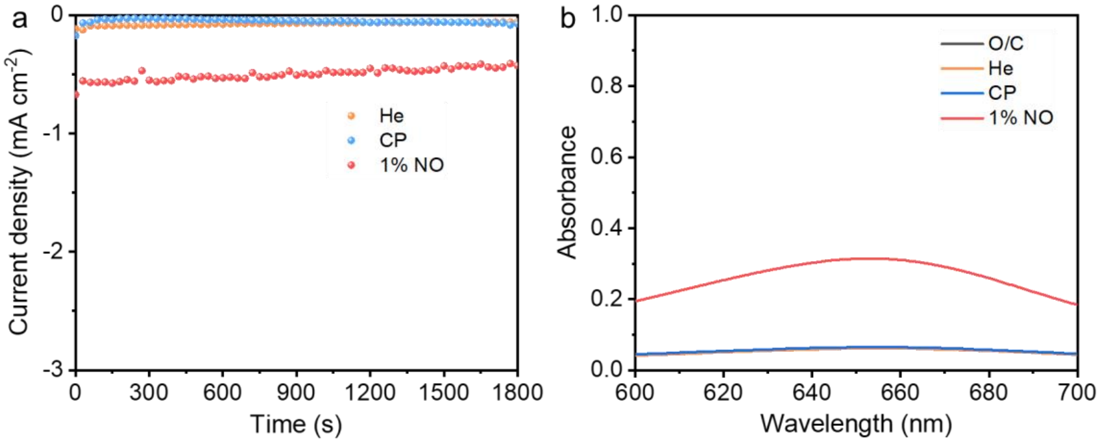

Figure S47. (a) i-t curves under different test conditions for excluding possible interferences from any contaminants. (b) UV-vis absorption spectra of catholytes after electrolysis under different test conditions.

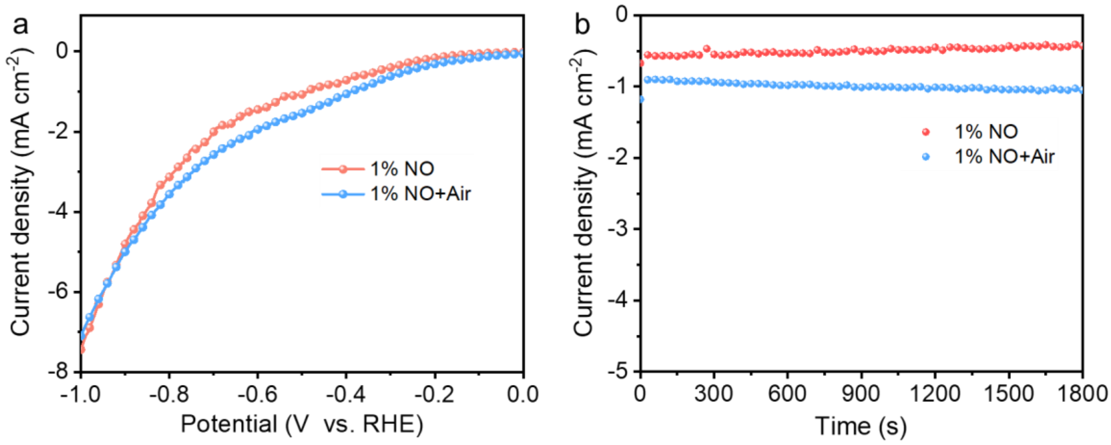

Figure S48. (a) LSV curves of RuFeCoNiCuZnO measured in 1% NO-saturated and 1% NO + Air saturated 0.5 M Na_2SO_4. (b) Corresponding i-t curves at -0.4 V vs. RHE.

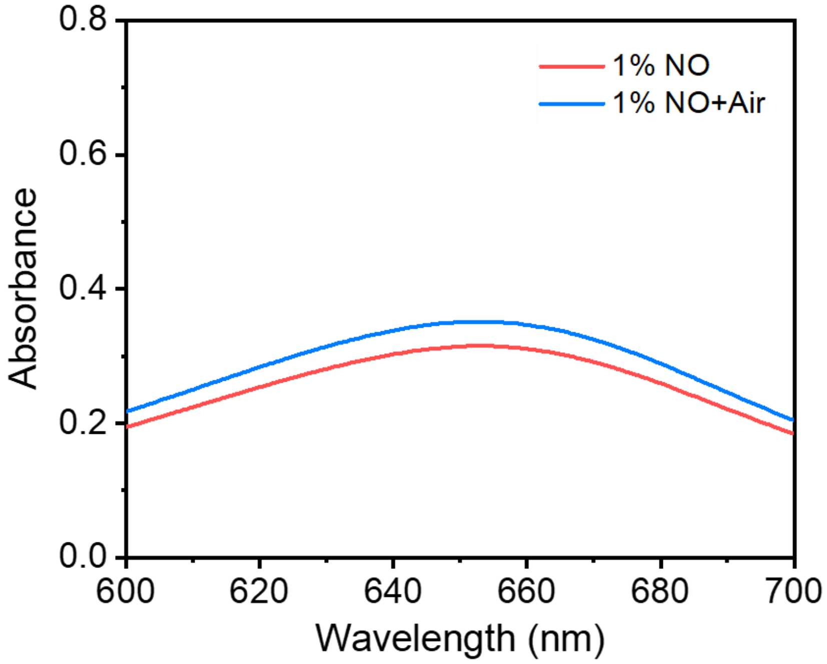

Figure S49. UV-Vis absorption spectra of catholytes for RuFeCoNiCuZnO in a gas-tight (1% NO) and open (1% NO + Air) chambers at -0.4 V vs. RHE.

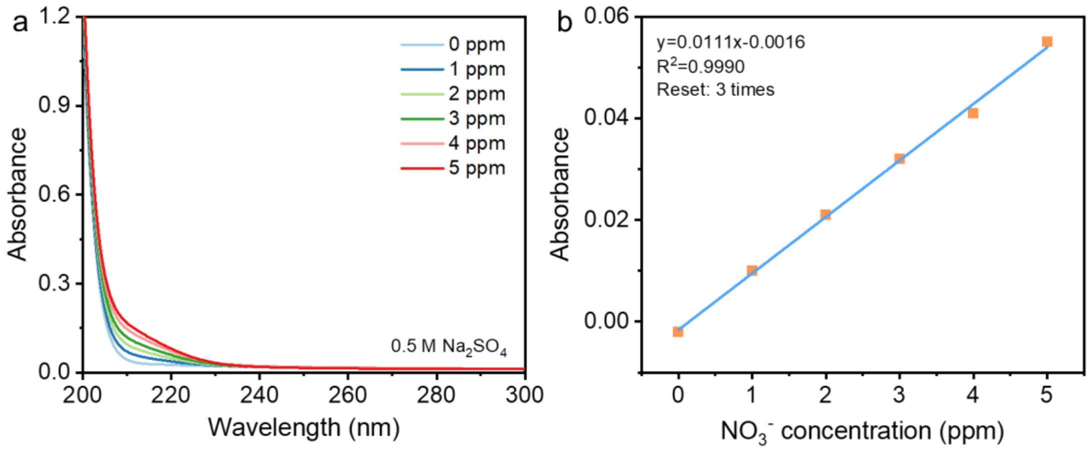

Figure S50. (a) UV-Vis absorption spectra of standard nitrate solution at room temperature. (b) Calibration curve used for estimation of NO_3^- concentrations.

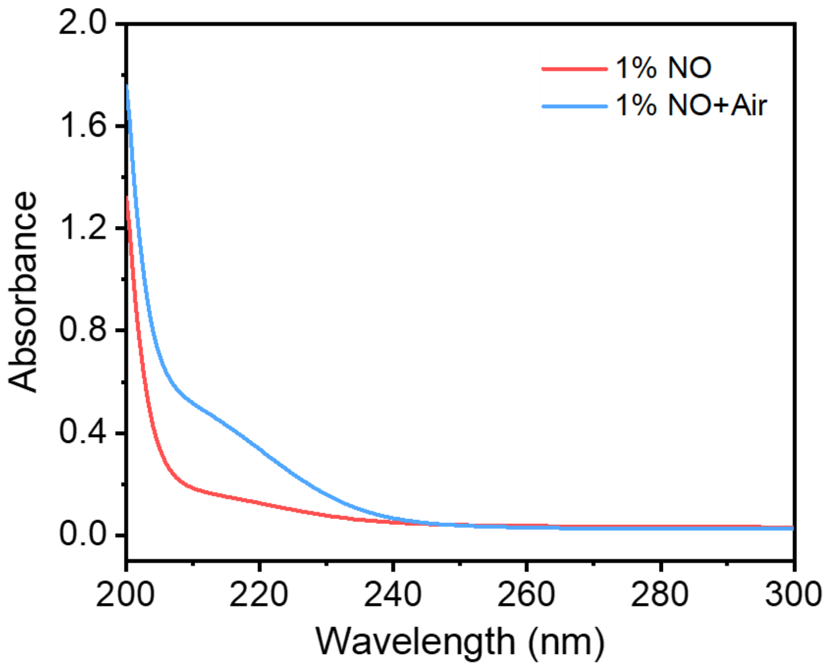

Figure S51. UV-Vis absorption spectra of NO_3^- for RuFeCoNiCuZnO in a gas-tight (1% NO) and open (1% NO + Air) chambers at -0.4 V vs. RHE.

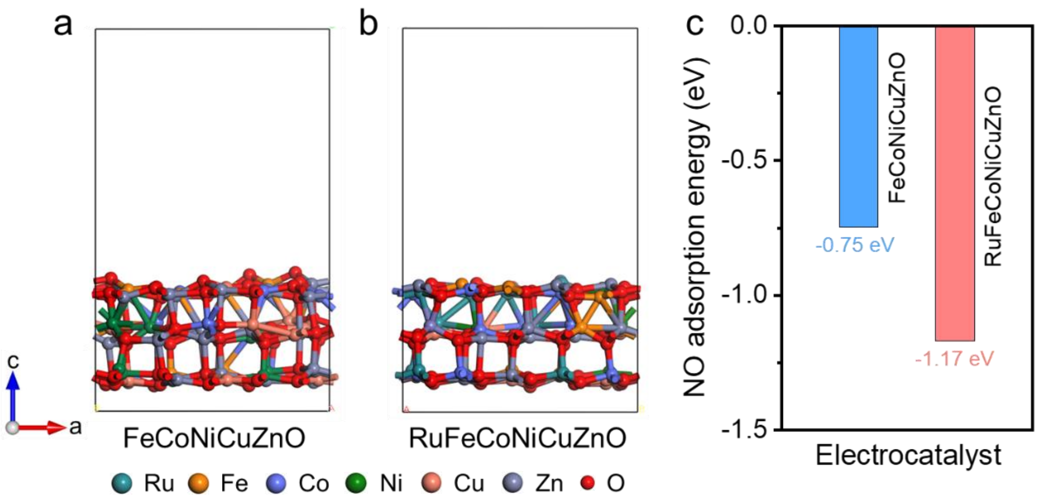

Figure S52. The optimized geometry structures for (a) FeCoNiCuZnO and (b) RuFeCoNiCuZnO. (c) The calculated NO adsorption energy on surface of FeCoNiCuZnO and RuFeCoNiCuZnO.

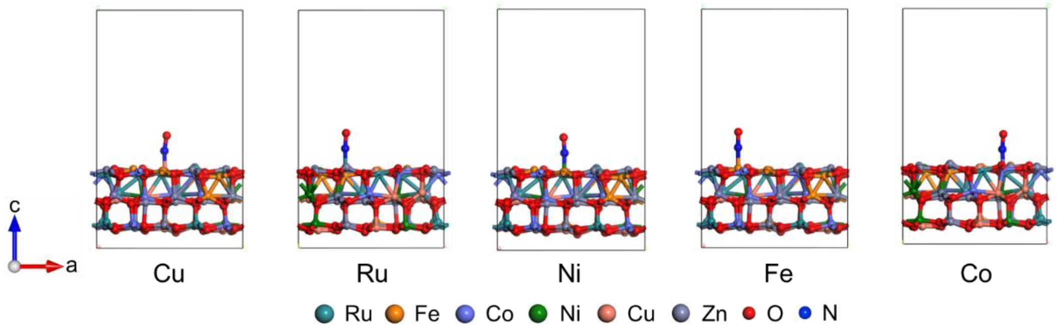

Figure S53. The calculated NO adsorption energies at various metal sites in RuFeCoNiCuZnO.

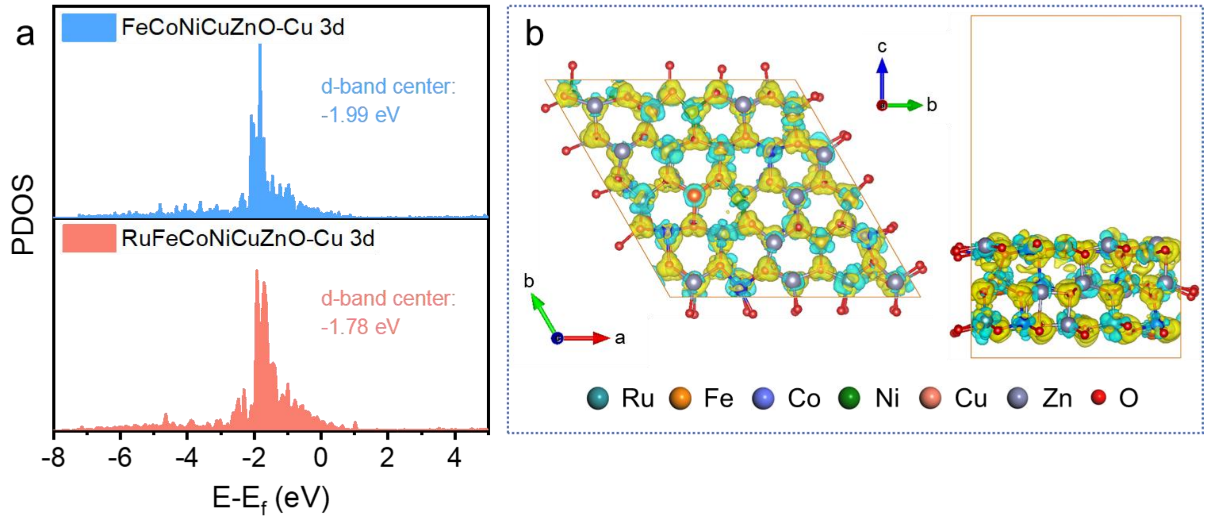

Figure S54. (a) The projected density of states (PDOS) of FeCoNiCuZnO and RuFeCoNiCuZnO. (b) The charge density difference of RuFeCoNiCuZnO. The accumulation and depletion of the electron density are marked in yellow and cyan, respectively.

The charge density difference analysis reveals a significant charge redistribution between Ru and O atoms upon Ru incorporation. This redistribution thereby modulates the electronic structure of adjacent Cu sites. The PDOS analysis provides more explicit insight into the altered electronic states of Cu.

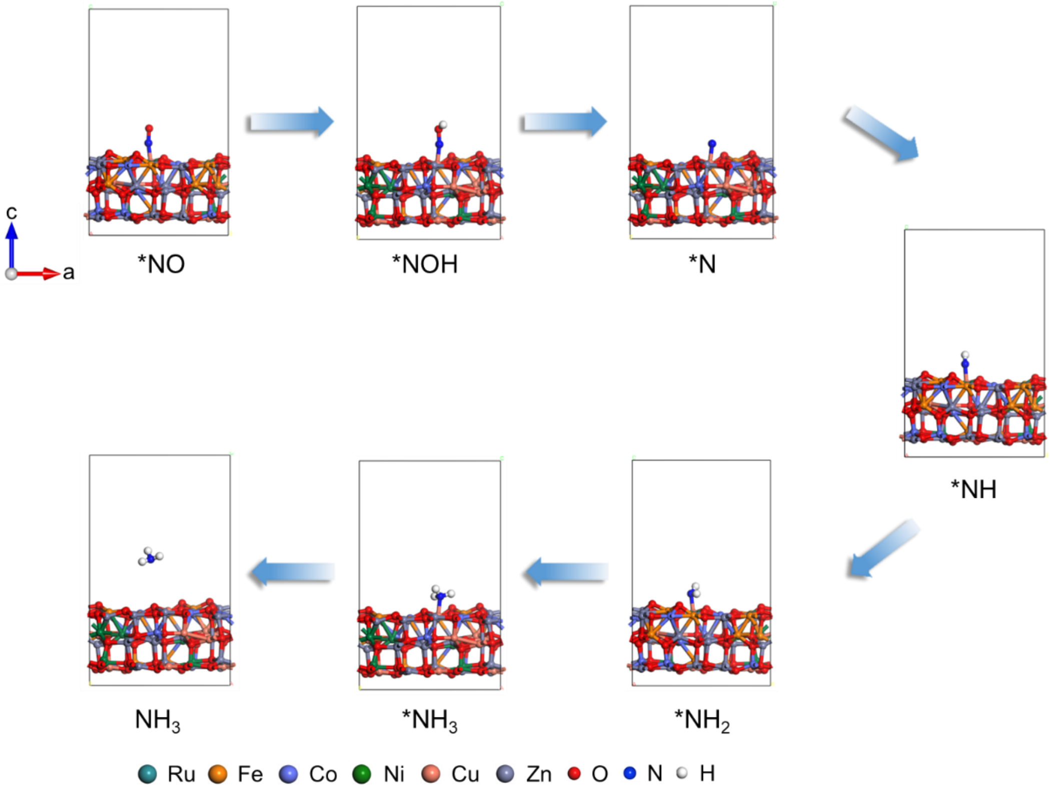

Figure S55. The optimized geometry structures of NORR intermediates over FeCoNiCuZnO.

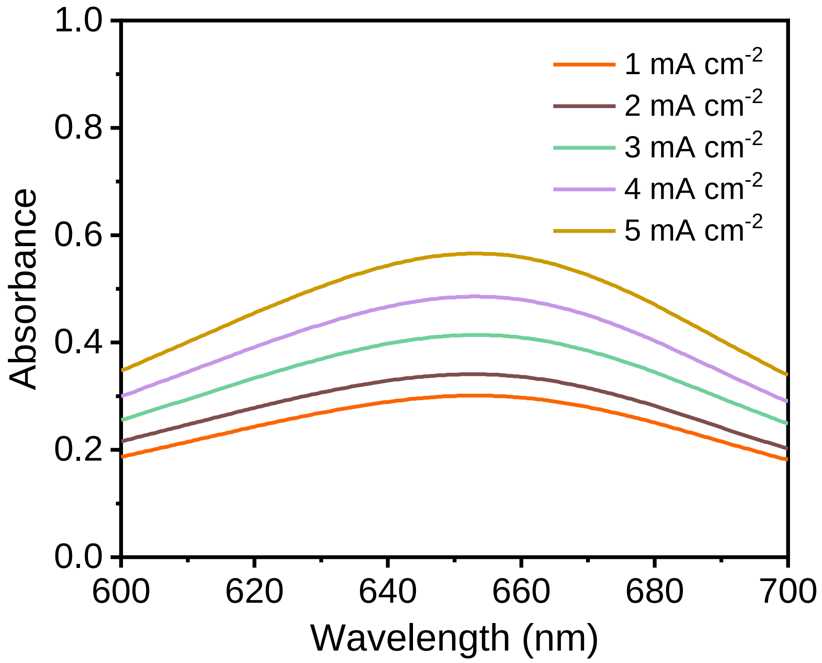

Figure S56. Corresponding UV-Vis absorption spectra of the electrolytes after the Zn-NO battery test.

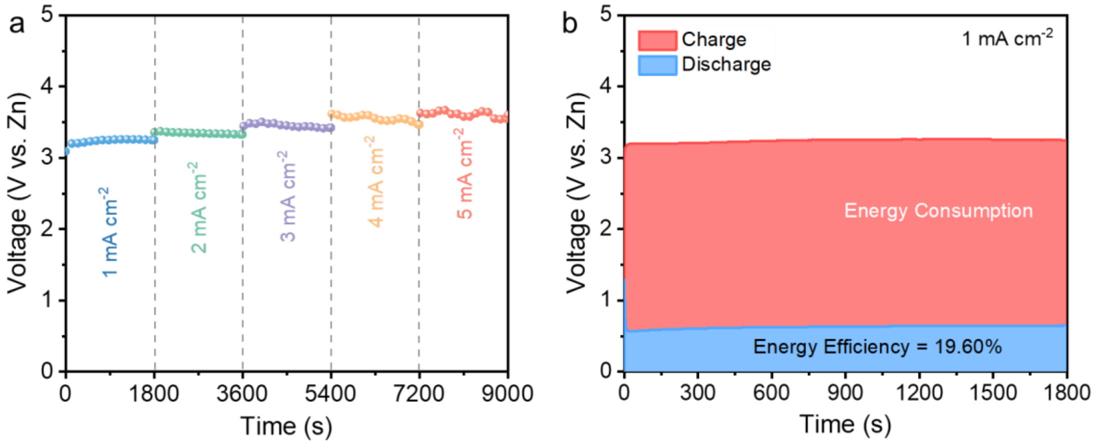

Figure S57. (a) The charge voltage profiles at different current densities. (b) The calculated energy efficiency of the Zn-NO/OER battery based on RuFeCoNiCuZnO.

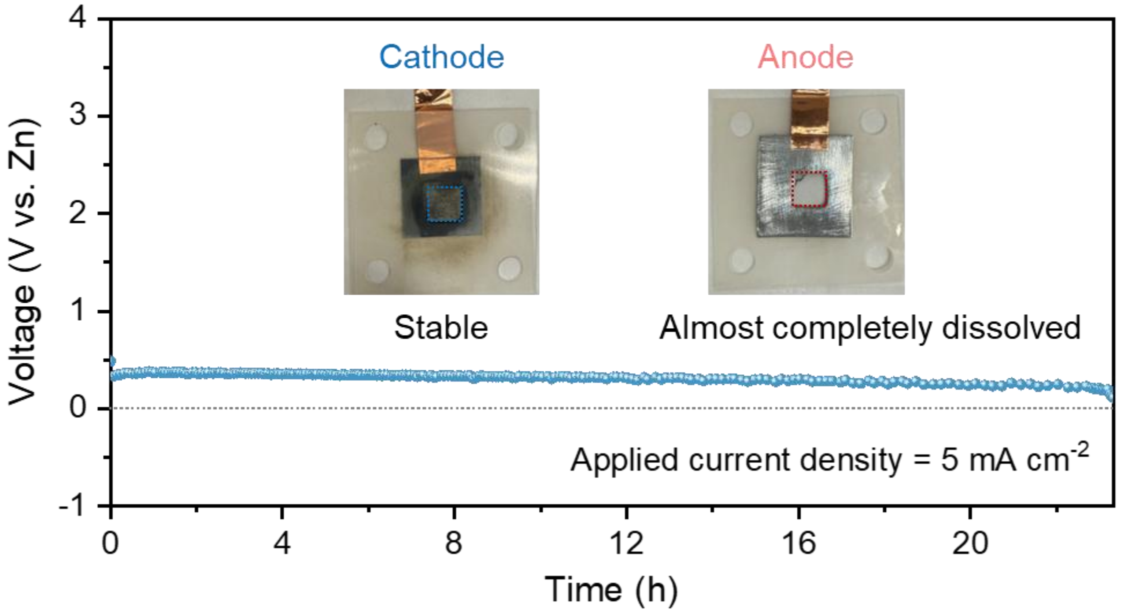

Figure S58. The voltage stability test during discharge at a current density of 5 mA cm^{-2}.

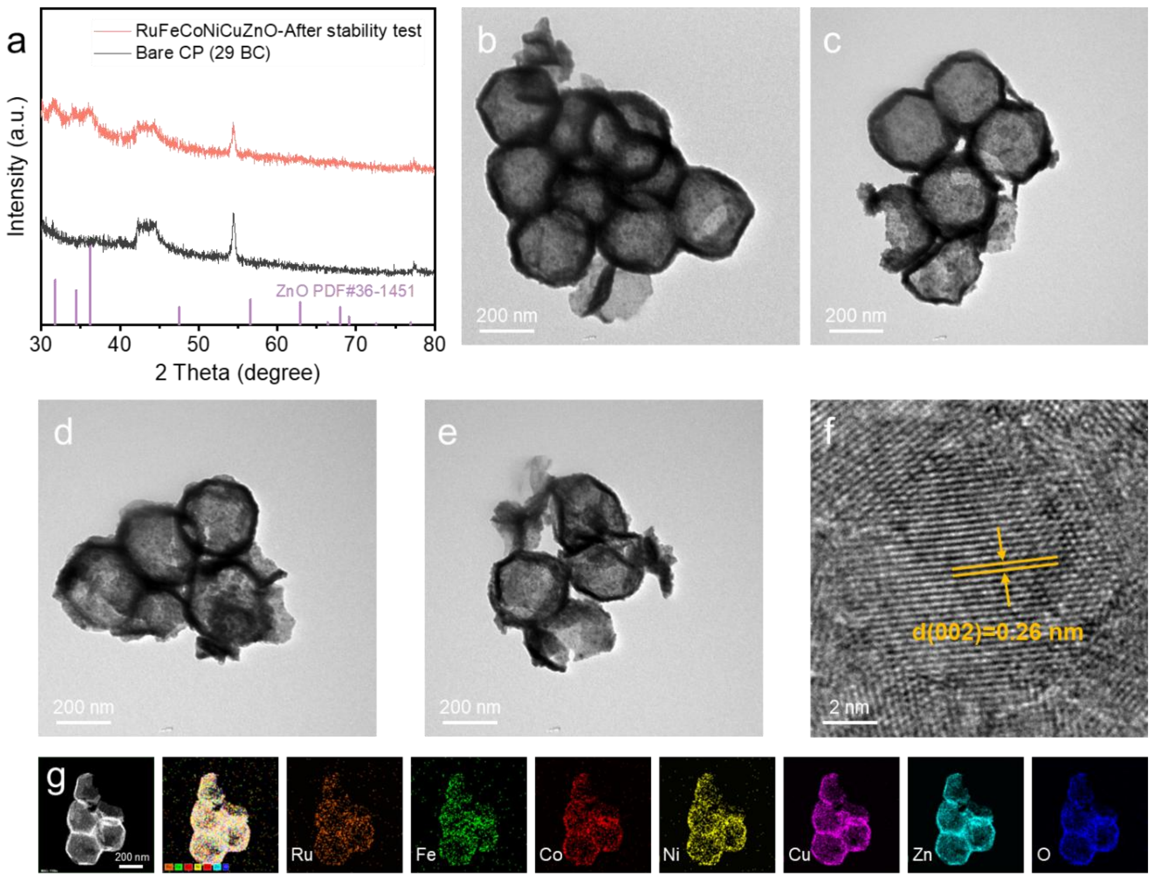

Figure S59. (a) XRD patterns of the RuFeCoNiCuZnO after 23 h stability and bare CP (29 BC) (Note: the peaks at around 2 theta = 43, 55, and 77 degree originate from the purchased commercial CP, 29 BC). (b, c, d, and e) TEM images of the RuFeCoNiCuZnO at different positions after the 23-h stability test (Note: nafion serves as an interfacial binder between adjacent polyhedral units). (f) HRTEM image of the RuFeCoNiCuZnO after the 23-h stability test. (g) HAADF-STEM image and the corresponding elemental mapping images of RuFeCoNiCuZnO after the 23-h stability test.

Table S1. The actual metal content in FeCoNiCuZnO determined by ICP-OES.

Element	Mass (wt.%)	Atom (%)
Fe	0.66	0.39
Co	2.79	1.56
Ni	2.75	1.54
Cu	6.89	3.54
Zn	55.19	27.76

Table S2. The actual metal content in RuFeCoNiCuZnO determined by ICP-OES.

Element	Mass (wt.%)	Atom (%)
Ru	2.0	0.65
Fe	0.31	0.18
Co	2.75	1.52
Ni	3.10	1.72
Cu	6.63	3.38
Zn	52.79	26.36

Table S3. Fitting parameters of Cu K-edge EXAFS curves for FeCoNiCuZnO and RuFeCoNiCuZnO.

Sample	Path	R (Å)	CN	$\Delta\sigma^2$ (Å^2)	ΔE_0 (eV)	R factor
FeCoNiCuZnO	Cu-O	1.97	2.61	0.00535	-1.845	0.84%
	Cu-M	2.92	2.74	0.00845		
RuFeCoNiCuZnO	Cu-O	1.89	2.39	0.00548	-4.752	1.28%
	Cu-M	2.90	1.12	0.01644		

Table S4. Comparison of NH_3 yield and Faraday efficiency (FE) of RuFeCoNiCuZnO with some reported NORR catalysts.

Catalyst	C_{NO}	Electrolyte (Na_2SO_4/ Li_2SO_4)	NH_3 Yield	FE (%)	Potential (V vs. RHE)	Ref.
**RuFeCoNi CuZnO**	**1 vol %**	**0.5 M**	**104.03 μg h^{-1} mg_{cat}^{-1}**	**99.08**	**–0.4 V**	**This work**
Cu-Co_3O_4 NBs	1 vol %	0.5 M	54.28 μg h^{-1} mg_{cat}^{-1}	94.13	–0.4 V	4
Cu-Co_3O_4/CuO NBs			59.10 μg h^{-1} mg_{cat}^{-1}	93.19		
hcp-RuCo	1 vol %	0.25 M	77.76μg h^{-1} mg_{cat}^{-1}	99.2	–0.1 V	5
Au/C	1 mM dissolved NO	0.5 M	49.57 ug h^{-1} cm^{-2}	/	–0.3V	6
Ru-LCN	1 vol %	0.5 M	382.67 ug h^{-1} cm^{-2}	65.96	–0.2 V	7
Cu@Co	1 vol %	0.1 M	627.20 ug h^{-1} cm^{-2}	76.54	–0.5 V	8
Vo-TiO_{2-x}	1 vol %	1 M KOH	632.9 μg h^{-1} mg_{cat}^{-1}	76.4	–0.5 V	9
FeOCl-V_{Cl}	1 vol %	0.1 M HCl+50 mM SB	455.4 μg h^{-1} mg_{cat}^{-1}	91.06	–0.5 V	10
Ni_2P/CP	10 vol %	0.1 M HCl	406.4 μg h^{-1} mg_{cat}^{-1}	76.9	–0.2 V	11
MnO_{2-x} NA/TM	10 vol %	0.2 M	168.30 ug h^{-1} cm^{-2}	82.8	–0.7 V	12
$Ru_{0.05}Cu_{0.95}$	20 vol %	0.5 M	300.56 ug h^{-1} cm^{-2}	64.9	−0.5 V	13
HCNF/CP	10 vol %	0.2 M	379.95 ug h^{-1} cm^{-2}	88.33	–0.6 V	14
Fe_2O_3/CP	10 vol %	0.1 M Na_2SO_4 + 0.5 mM Fe^{2+}EDTA	1326.32 ug h^{-1} cm^{-2}	86.73	–0.4 V	15
NiO nanosheet array	10 vol %	0.1 M Na_2SO_4 + 0.5 mM Fe^{2+}EDTA	2130.10 ug h^{-1} cm^{-2}	90	–0.6 V	16

Table S5. Comparison of power density of the present RuFeCoNiCuZnO-based Zn-NO battery with some reported Zn-NO/N_2 batteries.

Catalyst	Battery systems	Power density	Ref.
**RuFeCoNiCuZnO**	**metal-NO**	**1.18 mW cm^{-2}**	**This work**
Cu-2	metal-N_2	0.0101 mW cm^{-2}	[17]
VN@NSC-900	metal-N_2	0.01642 mW cm^{-2}	[18]
Fe 1.0 HTNs	metal-N_2	0.02765 mW cm^{-2}	[19]
NbS_2	metal-N_2	0.31 mW cm^{-2}	[20]
CoPi/HSNPC	metal-N_2	0.31 mW cm^{-2}	[21]
CoPi/NPCS	metal-N_2	0.49 mW cm^{-2}	[22]
CoP/TM	metal-NO	0.496 mW cm^{-2}	[23]
MoC/P	metal-NO	0.77 mW cm^{-2}	[24]
TiO_{2-x}/TP	metal-NO	0.84 mW cm^{-2}	[25]
c-VN/CF	metal-NO	0.85 mW cm^{-2}	[26]
NiO/TM	metal-NO	0.88 mW cm^{-2}	[16]
MoS_2/GF	metal-NO	1.04 mW cm^{-2}	[27]

Table S6. The advantages of Zn-NO battery compared to conventional electrolytic NO-to-ammonia conversion processes.

Comparison content	Conventional electrolysis	Zn-NO battery	Advantages
Energy input	External power source required	No additional power supply required	Self-powered
Product value	NH_3	Electricity + NH_3	Simultaneous export of energy and chemical
Environmental adaptability	High concentration of NO and stable power supply are required	Tolerant to NO fluctuations (such as industrial flue gas)	Suitable for exhaust gas treatment
System complexity	Electrolyzer required	Integrated battery structure	Suitable for portable/distributed applications

Supplementary References

1. Kohn, W.; Sham, L. J., Self-Consistent Equations Including Exchange and Correlation Effects. *Phys. Rev.* **1965,** *140* (4A), A1133-A1138.

2. Kresse, G.; Hafner, J., Ab Initio Molecular Dynamics for Open-Shell Transition Metals. *Phys. Rev. B* **1993,** *48* (17), 13115-13118.

3. Perdew, J. P.; Burke, K.; Ernzerhof, M., Generalized Gradient Approximation Made Simple. *Phys. Rev. Lett.* **1996,** *77* (18), 3865-3868.

4. Wang, D.; Fan, G.; Pei, Z.; Luan, D.; Gu, X.; Lou, X. W., Efficient Cu—Co Dual-Sites in Cobalt Oxide Nanoboxes for Electrocatalytic Reduction of Low-Concentration NO to NH_3. *Adv. Mater.* **2025,** *37* (34), 2504497.

5. Wang, D.; Fan, G.; Luan, D.; Guo, Y.; Gu, X.; Lou, X. W., Ru-Incorporation-Induced Phase Transition in Co Nanoparticles for Low-Concentration Nitric Oxide Electroreduction to Ammonia at Low Potential. *Adv. Mater.* **2024,** *36* (50), 2408580.

6. Choi, J.; Du, H.-L.; Nguyen, C. K.; Suryanto, B. H. R.; Simonov, A. N.; MacFarlane, D. R., Electroreduction of Nitrates, Nitrites, and Gaseous Nitrogen Oxides: A Potential Source of Ammonia in Dinitrogen Reduction Studies. *ACS Energy Lett.* **2020,** *5* (6), 2095-2097.

7. Li, Y.; Cheng, C.; Han, S.; Huang, Y.; Du, X.; Zhang, B.; Yu, Y., Electrocatalytic Reduction of Low-Concentration Nitric Oxide into Ammonia over Ru Nanosheets. *ACS Energy Lett.* **2022,** *7* (3), 1187-1194.

8. Wu, Z.; Liu, Y.; Wang, D.; Zhang, Y.; Gu, K.; He, Z.; Liu, L.; Liu, H.; Fan, J.; Chen, C.; Wang, S., Cu@Co with Dilatation Strain for High-Performance Electrocatalytic Reduction of Low-Concentration Nitric Oxide. *Adv. Mater.* **2024,** *36* (11), 2309470.

9. Guo, X.; Wu, T.; Li, H.; Chai, L.; Liu, M., Enhancing Low-Concentration Electroreduction of NO to NH_3 Via Potential-Controlled Active Site-Intermediate Interactions. *Angew. Chem. Int. Ed.* **2025,** *64* (8), e202420346.

10. Guo, X.; Wang, P.; Wu, T.; Wang, Z.; Li, J.; Liu, K.; Fu, J.; Liu, M.; Wu, J.; Lin, Z.; Chai, L.; Bian, Z.; Li, H.; Liu, M., Aqueous Electroreduction of Nitric Oxide to Ammonia at Low Concentration Via Vacancy Engineered FeOCl. *Angew. Chem. Int. Ed.* **2024,** *63* (6), e202318792.

11. Mou, T.; Liang, J.; Ma, Z.; Zhang, L.; Lin, Y.; Li, T.; Liu, Q.; Luo, Y.; Liu, Y.; Gao, S.; Zhao, H.; Asiri, A. M.; Ma, D.; Sun, X., High-Efficiency Electrohydrogenation of Nitric Oxide to Ammonia on a Ni_2P Nanoarray under Ambient Conditions. *J. Mater. Chem. A* **2021,** *9* (43), 24268-24275.

12. Li, Z.; Ma, Z.; Liang, J.; Ren, Y.; Li, T.; Xu, S.; Liu, Q.; Li, N.; Tang, B.; Liu, Y.; Gao, S.; Alshehri, A. A.; Ma, D.; Luo, Y.; Wu, Q.; Sun, X., MnO_2 Nanoarray with Oxygen Vacancies: An Efficient Catalyst for NO Electroreduction to NH_3 at Ambient Conditions. *Mater. Today Phys.* **2022,** *22*, 100586.

13. Shi, J.; Wang, C.; Yang, R.; Chen, F.; Meng, N.; Yu, Y.; Zhang, B., Promoting Nitric Oxide Electroreduction to Ammonia over Electron-Rich Cu Modulated by Ru Doping. *Sci. China Chem.* **2021,** *64* (9), 1493-1497.

14. Ouyang, L.; Zhou, Q.; Liang, J.; Zhang, L.; Yue, L.; Li, Z.; Li, J.; Luo, Y.; Liu, Q.; Li, N.; Tang, B.; Ali Alshehri, A.; Gong, F.; Sun, X., High-Efficiency NO Electroreduction to NH_3 over Honeycomb Carbon Nanofiber at Ambient Conditions. *J. Colloid. Inter. Sci.* **2022,** *616*, 261-267.

15. Liang, J.; Chen, H.; Mou, T.; Zhang, L.; Lin, Y.; Yue, L.; Luo, Y.; Liu, Q.; Li, N.; Alshehri, A. A.; Shakir, I.; Agboola, P. O.; Wang, Y.; Tang, B.; Ma, D.; Sun, X., Coupling Denitrification and Ammonia Synthesis Via Selective Electrochemical Reduction of Nitric Oxide over Fe_2O_3 Nanorods. *J. Mater. Chem. A* **2022,** *10* (12), 6454-6462.

16. Liu, P.; Liang, J.; Wang, J.; Zhang, L.; Li, J.; Yue, L.; Ren, Y.; Li, T.; Luo, Y.; Li, N.; Tang, B.; Liu, Q.; Asiri, A. M.; Kong, Q.; Sun, X., High-Performance NH_3 Production Via NO Electroreduction over a NiO Nanosheet Array. *Chem. Commun.* **2021,** *57* (99), 13562-13565.

17. Du, C.; Gao, Y.; Wang, J.; Chen, W., Achieving 59% Faradaic Efficiency of the N_2 Electroreduction Reaction in an Aqueous Zn–N_2 Battery by Facilely Regulating the Surface Mass Transport on Metallic Copper. *Chem. Commun.* **2019,** *55* (85), 12801-12804.

18. Lv, X.-W.; Liu, Y.; Wang, Y.-S.; Liu, X.-L.; Yuan, Z.-Y., Encapsulating Vanadium Nitride Nanodots into N,S-Codoped Graphitized Carbon for Synergistic Electrocatalytic Nitrogen Reduction and Aqueous Zn-N_2 Battery. *Appl. Catal. B* **2021,** *280*, 119434.

19. Lv, X.-W.; Liu, X.-L.; Gao, L.-J.; Liu, Y.-P.; Yuan, Z.-Y., Iron-Doped Titanium Dioxide Hollow Nanospheres for Efficient Nitrogen Fixation and Zn–N_2 Aqueous Batteries. *J. Mater. Chem. A* **2021,** *9* (7), 4026-4035.

20. Wang, H.; Si, J.; Zhang, T.; Li, Y.; Yang, B.; Li, Z.; Chen, J.; Wen, Z.; Yuan, C.; Lei, L.; Hou, Y., Exfoliated Metallic Niobium Disulfate Nanosheets for Enhanced Electrochemical Ammonia Synthesis and Zn-N2 Battery. *Appl. Catal. B* **2020,** *270*, 118892.

21. Ren, J.-T.; Chen, L.; Liu, Y.; Yuan, Z.-Y., Hollow Cobalt Phosphate Microspheres for Sustainable Electrochemical Ammonia Production through Rechargeable Zn–N_2 Batteries. *J. Mater. Chem. A* **2021,** *9* (18), 11370-11380.

22. Ren, J.-T.; Chen, L.; Wang, H.-Y.; Yuan, Z.-Y., Aqueous Rechargeable Zn–N_2 Battery Assembled by Bifunctional Cobalt Phosphate Nanocrystals-Loaded Carbon Nanosheets for Simultaneous NH_3 Production and Power Generation. *ACS Appl. Mater. Inter.* **2021,** *13* (10), 12106-12117.

23. Liang, J.; Hu, W.-F.; Song, B.; Mou, T.; Zhang, L.; Luo, Y.; Liu, Q.; Alshehri, A. A.; Hamdy, M. S.; Yang, L.-M.; Sun, X., Efficient Nitric Oxide Electroreduction toward Ambient Ammonia Synthesis Catalyzed by a CoP Nanoarray. *Inorg. Chem. Front.* **2022,** *9* (7), 1366-1372.

24. Meng, G.; Jin, M.; Wei, T.; Liu, Q.; Zhang, S.; Peng, X.; Luo, J.; Liu, X., MoC Nanocrystals Confined in N-Doped Carbon Nanosheets toward Highly Selective Electrocatalytic Nitric Oxide Reduction to Ammonia. *Nano Res.* **2022,** *15* (10), 8890-8896.

25. Li, Z.; Zhou, Q.; Liang, J.; Zhang, L.; Fan, X.; Zhao, D.; Cai, Z.; Li, J.; Zheng, D.; He, X.; Luo, Y.; Wang, Y.; Ying, B.; Yan, H.; Sun, S.; Zhang, J.; Alshehri, A. A.; Gong, F.; Zheng, Y.; Sun, X., Defective TiO_{2-x} for High-Performance Electrocatalytic NO Reduction toward Ambient NH_3 Production. *Small* **2023,** *19* (24), 2300291.

26. Qi, D.; Lv, F.; Wei, T.; Jin, M.; Meng, G.; Zhang, S.; Liu, Q.; Liu, W.; Ma, D.; Hamdy, M. S.; Luo, J.; Liu, X., High-Efficiency Electrocatalytic NO Reduction to NH_3 by Nanoporous VN. *Nano Res. Energy* **2022,** *1*, e9120022.

27. Zhang, L.; Liang, J.; Wang, Y.; Mou, T.; Lin, Y.; Yue, L.; Li, T.; Liu, Q.; Luo, Y.; Li, N.; Tang, B.; Liu, Y.; Gao, S.; Alshehri, A. A.; Guo, X.; Ma, D.; Sun, X., High-Performance Electrochemical NO Reduction into NH_3 by MoS_2 Nanosheet. *Angew. Chem. Int. Ed.* **2021,** *60* (48), 25263-25268.
